# The Role of Digital Biomarkers in Physiological Signal-Based Depression Assessment: Systematic Review and Meta-Analysis

**DOI:** 10.2196/76432

**Published:** 2026-04-02

**Authors:** Hyeongsuk Lee, Seung-Gul Kang, SeonHeui Lee

**Affiliations:** 1 Research Institute of AI and Nursing Science, College of Nursing, Gachon University, 191 Hambangmoe-ro, Yeonsu-gu, Incheon, 21936, Republic of Korea, 82 32-820-4230; 2 Department of Psychiatry, Gil Medical Center, Gachon University College of Medicine, Incheon, Republic of Korea

**Keywords:** digital biomarkers, depression, wearable electronic devices, sleep quality, ambulatory monitoring

## Abstract

**Background:**

Digital biomarkers are increasingly being used to support depression assessment by providing objective, continuous, and real-time physiological and behavioral data. However, most existing studies have focused on individual biomarkers, such as sleep or cardiac parameters, while integrative evaluations that capture the multidimensional nature of depression remain limited.

**Objective:**

This systematic review evaluated digital biomarkers for depression and synthesized evidence on differences between individuals with depression and controls.

**Methods:**

Eligible studies included observational or interventional studies examining digital biomarkers for depression with validated outcome measures. We searched major international and Korean databases, including MEDLINE, PsycINFO, CINAHL, IEEE Xplore, Web of Science, Cochrane Library, KISS, RISS, KMbase, and KoreaMed, from inception to December 28, 2025. Risk of bias was assessed using the Quality Assessment of Diagnostic Accuracy Studies-2 tool and the Scottish Intercollegiate Guidelines Network checklist. Meta-analyses were conducted using random-effects models with the Hartung-Knapp-Sidik-Jonkman method, and other outcomes were narratively summarized.

**Results:**

The search yielded 39,617 records, of which 132 studies involving 57,852 participants met the inclusion criteria. These studies encompassed various digital biomarkers, including sleep, physical activity, cardiac measures, smartphone-derived data, speech, GPS data, and circadian rhythms. A meta-analysis of 22 studies (6947 participants) revealed that individuals with depression had significantly longer sleep onset latency (5 studies; n=292; +4.75 min, 95% CI 2.46-7.04; *P*=.005; 95% prediction interval [PI] 0.01-10.27) and time in bed (3 studies; n=236; +31.81 min, 95% CI 18.22-45.39; *P*=.01; 95% PI 2.28-55.16). Physical activity counts were also significantly lower (5 studies; n=462; standardized mean difference −0.71, 95% CI −1.33 to −0.09; *P*=.03; 95% PI −2.18 to 0.71). Although individuals with depression showed a lower sleep efficiency, higher mean heart rate, and lower SD of normal-to-normal intervals, these differences were not statistically significant. Other digital markers yielded inconsistent results. Overall, these findings indicate that no single digital biomarker sufficiently captures depression-related changes. Instead, the results support the superiority of personalized, multimodal approaches. However, the generalizability of these findings is limited by the lack of standardized data collection protocols and high clinical heterogeneity across studies, as reflected in wide PIs.

**Conclusions:**

Certain digital biomarkers, particularly sleep onset latency and physical activity counts, showed consistent average differences between the depression and control groups. However, wide PIs indicate substantial variability across settings, suggesting that no single marker is sufficient for reliable detection. This study advances the field by providing a comprehensive meta-analysis of multidimensional digital biomarkers, establishing a quantitative foundation for objective depression screening and monitoring. These findings support the use of personalized, multimodal digital phenotyping approaches and highlight the need for standardized, clinically interpretable frameworks for real-world depression monitoring.

## Introduction

Depression is a significant mental health issue that affects over 300 million people worldwide and is one of the leading causes of disability-adjusted life years [1]. However, the current diagnostic process for depression largely relies on self-reported questionnaires and subjective clinical judgment, raising concerns regarding its accuracy and consistency [2]. These “snapshot” evaluations often fail to capture the dynamic, fluctuating nature of depressive symptoms in real-world settings, leading to delayed interventions and suboptimal treatment outcomes. To bridge this gap, digital biomarkers have emerged as a transformative objective approach, enabling the moment-by-moment quantification of individual-level human phenotypes in situ using data from personal digital devices [3,4].

Digital biomarkers, derived from smartphones, wearables, and ambient sensors, provide continuous, noninvasive, and high-frequency longitudinal data [3,5]. These markers encompass a wide range of clinical dimensions, including sleep patterns, physical activity levels, heart rate variability (HRV), vocal characteristics, and social interaction data. Recent advancements in sensor technology have significantly enhanced the precision of these metrics, offering unprecedented insights into the physiological and behavioral underpinnings of mood disorders [6,7]. These digital biomarkers possess unique properties that indicate their potential to complement or even replace traditional subjective methods of diagnosing depression. However, as the field matures, a critical challenge has surfaced regarding the inconsistency across various research findings [8,9].

While numerous studies have identified potential biomarkers, significant variability in hardware, data collection duration, and analytical pipelines has led to fragmented findings, raising concerns regarding their reproducibility and generalizability [10-12]. Although attempts have been made to conduct systematic reviews focused on digital biomarkers related to depression, these efforts have often been hindered by issues, such as the limited number of related studies or data heterogeneity, making meta-analyses infeasible [4]. Consequently, existing review reports are confined to specific biomarkers (eg, HRV or sleep data) or merely categorize and summarize findings without deeper integration [13,14]. Given that depression is a systemic disorder characterized by complex interactions between biological rhythms and behavioral shifts, isolated metrics are insufficient to capture its full multidimensionality. The lack of comprehensive systematic reviews poses a critical barrier to understanding the clinical utility and practical implementation of digital biomarkers.

There is, therefore, an urgent clinical and scientific need for a comprehensive, multimodal meta-analysis. Such an investigation is essential to distinguish “robust indicators” from “context-specific noise” and to establish the pooled effect sizes necessary for developing reliable diagnostic algorithms. By synthesizing evidence across diverse domains, including sleep, physical activity, cardiac measures, smartphone usage, speech, GPS, and circadian parameters, this systematic review aims to provide a comprehensive evaluation of the digital biomarker landscape. Specifically, this systematic review performs meta-analyses to quantify group differences between individuals with depression and controls without depression, thereby strengthening the evidence base for personalized, preemptive depression management in the digital health era.

## Methods

### Study Design and Registration

The protocol was prospectively registered in the PROSPERO database (CRD42024518136) and prepared following the PRISMA (Preferred Reporting Items for Systematic Reviews and Meta-Analyses) protocol checklist (Checklist 1).

### Ethical Considerations

This systematic review was exempted by the Institutional Review Board (IRB) of Gachon University (IRB number: 1044396‐202403 HR-052-01).

### Eligibility Criteria

The inclusion criteria were as follows: (1) inclusion of participants with depression, (2) use of digital biomarkers to assess depression severity, and (3) reporting diagnostic concordance with validated assessment tools. The exclusion criteria were as follows: (1) non-English or non-Korean articles, (2) duplication, (3) inaccessible full text, and (4) reviews or qualitative studies.

### Information Sources and Search Strategy

This systematic review involved a search of major academic databases, including the Cochrane Library (Wiley), MEDLINE (Ovid), PsycINFO (Ovid), CINAHL (EBSCOhost), IEEE Xplore (IEEE), Web of Science (Clarivate), and Korean academic databases, such as KISS, RISS, KMbase, and KoreaMed. The final search of all sources was conducted on December 28, 2025. Trial registries, gray literature, and author contacts were omitted as the primary search provided sufficient data. Additionally, we manually screened reference lists and removed duplicates using EndNote 20.

The search strategy combined Medical Subject Headings (MeSH) and free-text keywords related to depression and digital biomarkers. These terms were adapted for each database to maximize search sensitivity. The key terms included “depressi*,” “MDD,” “wearable,” “application,” “smartwatch,” “biomarker*,” “sleep*,” “speech,” “behavioral parameter*,” “electroencephalogram,” and “electrocardiogram.” The search process followed the PRISMA Search Strategy (PRISMA-S) extension [15]. The full search strategy, including specific search strings, limits applied, and the number of records retrieved per database, is provided in Multimedia Appendix 1.

### Selection Process

Two independent reviewers screened the titles and abstracts after removing duplicates. Potentially relevant studies and manually identified records underwent full-text assessment. Any disagreements were resolved through consensus with a third reviewer.

### Data Collection Process and Data Extraction

Data were extracted by 2 independent reviewers using standardized forms. Extracted characteristics included author, year, country, study design, number of participants, age, sex, biomarker measurement device, measurement period, depression indicators, and analytical methods.

### Study Risk of Bias Assessment

Quality was assessed using the Quality Assessment of Diagnostic Accuracy Studies-2 (QUADAS-2) tool to evaluate diagnostic accuracy and the Scottish Intercollegiate Guidelines Network (SIGN) tool for case-control studies. All eligible studies were included irrespective of their quality scores.

### Effect Measures and Synthesis Methods

A meta-analysis was performed on quantitatively synthesizable digital biomarkers, and a systematic review was conducted on other biomarkers. All statistical analyses were performed in R software (version 4.5.0; R Foundation for Statistical Computing) using the “meta” and “pimeta” packages. Pooled effects, expressed as mean differences (MDs) or standardized mean differences (SMDs), were estimated using a random-effects model with the Hartung-Knapp-Sidik-Jonkman (HKSJ) method [16,17]. Restricted maximum likelihood was used for τ² estimation. Results have been presented with 95% CIs. To account for the distribution of true effects across different settings, 95% prediction intervals (PIs) were additionally calculated using the parametric bootstrap approach proposed by Nagashima et al [18], which is robust for small study numbers.

Parameters that could not be meta-analyzed due to reporting inconsistencies or insufficient data were narratively synthesized. These included specific sleep measures (eg, sleep fragmentation, rapid eye movement [REM] sleep, REM latency, and slow-wave sleep [SWS]); physical activity (eg, light physical activity [LPA] and energy expenditure); cardiac HRV indices (eg, the root mean square of successive differences [RMSSD], low frequency [LF], high frequency [HF], and LF/HF ratio); and parameters related to speech, GPS, and circadian rhythms.

### Reporting Bias Assessment and Certainty Assessment

Reporting bias was considered qualitatively, as a formal statistical assessment (eg, funnel plot) was infeasible due to fewer than 10 studies per outcome. This was supported by comprehensive database searches and manual screening of reference lists. The certainty of evidence was assessed using the GRADE (Grading of Recommendations, Assessment, Development, and Evaluation) approach, considering risk of bias, inconsistency, imprecision, and indirectness [19].

## Results

### Study Selection

**Figure 1.**
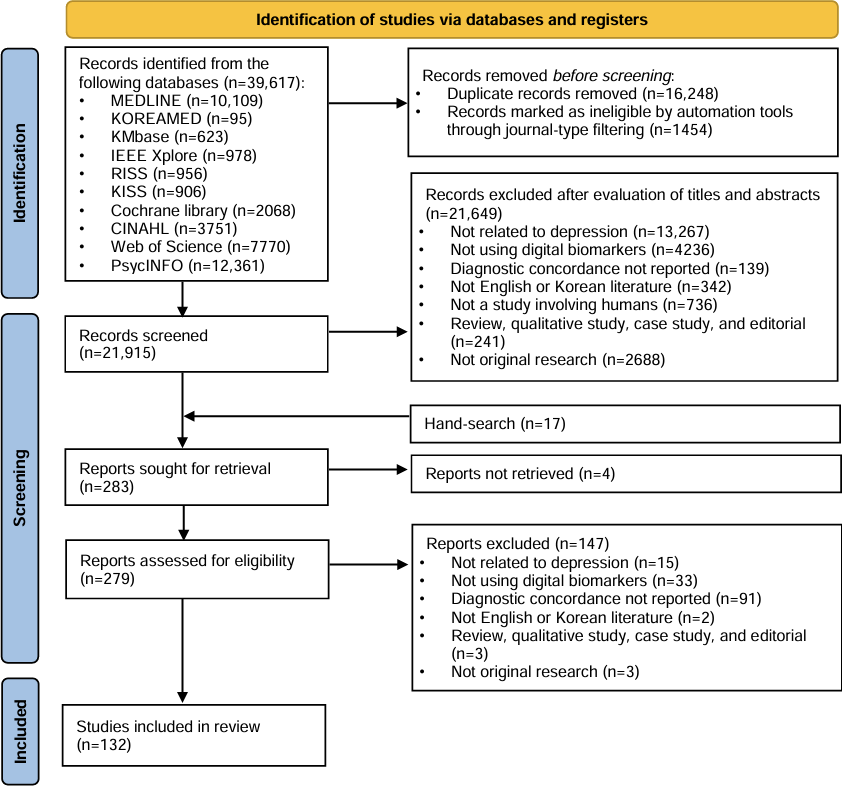
Flow diagram of the study selection process for examining the role of digital biomarkers in depression assessment.

The initial search yielded 39,617 studies. After removing duplicates and excluding records automatically identified as ineligible through journal-type filtering, 21,915 studies remained. Following the screening of titles and abstracts, 21,649 studies were excluded. Before conducting a full-text review, a manual search through reference lists and citation tracking of relevant systematic reviews identified 17 additional studies. Consequently, full-text reviews were conducted on 283 articles, of which 132 studies involving 57,852 participants met the inclusion criteria (Figure 1).

### Study Characteristics

The characteristics of the included studies are summarized in Tables S1 and S2 in Multimedia Appendix 2. The included studies were categorized based on the types of digital biomarkers investigated. A total of 87 studies used single parameters, including sleep parameters (n=23) [20-42], cardiac parameters (n=19) [43-61], physical activity parameters (n=16) [62-77], smartphone-based parameters (n=9) [78-86], speech parameters (n=10) [87-96], circadian rhythm parameters (n=7) [97-103], electroencephalogram parameters (n=2) [104,105], and video-based parameters (n=1) [106]. Furthermore, 45 studies used multiple parameters to assess digital biomarkers [4,107-150]. Of the 132 studies, 63 (47.7%) used wearable devices to continuously measure biomarkers in daily life, typically for more than a week. Study designs encompassed large-scale cohorts and group comparisons (eg, depression vs control). Analytical approaches varied, including regression analyses for associations, evaluations of diagnostic accuracy, and assessments of group differences (Multimedia Appendix 3).

Validated depression assessment tools were used, with the most frequent being the Patient Health Questionnaire (PHQ), followed by the Hamilton Depression Rating Scale (HDRS), Beck Depression Inventory (BDI), Geriatric Depression Scale (GDS), Korean version of the GDS (SGDS-K), and Center for Epidemiologic Studies Depression Scale (CES-D) (Multimedia Appendix 4). Biomarker categories and their detailed features are illustrated in Multimedia Appendix 5 and Table S3 in Multimedia Appendix 2.

### Risk of Bias in Studies

Based on the SIGN assessment, all 73 case-control studies demonstrated acceptable internal validity with clearly defined groups. However, participation rates for each group showed substantial variability, ranging from 20.1% to 100%, and many studies (70/73, 96%) did not report comparisons between participants and nonparticipants. Additionally, potential confounding factors were insufficiently addressed in several studies (Table S4 in Multimedia Appendix 2).

Among diagnostic accuracy studies assessed using QUADAS-2, most studies (58/59, 98%) showed a low risk of bias in the index test, reference standard, and flow and timing domains. In contrast, great concern regarding applicability was identified because study populations often did not align with intended clinical targets, limiting the generalizability of the findings to real-world settings (Table 1).

**Table 1.** Study quality assessment using QUADAS-2^a^.

First author	Year	Risk of bias^b^				Applicability concerns^b^		
		Patient selection	Index test	Reference standard	Flow and timing	Patient selection	Index test	Reference standard
Peng [21]	2023	1	1	1	1	1	1	1
Hasanzadeh [28]	2020	1	1	1	1	1	1	1
Ding [30]	2019	1	1	1	1	1	1	1
Coutts [50]	2020	2	1	1	1	2	1	1
Roh [55]	2014	1	1	1	1	2	1	1
Zhang [57]	2012	2	1	1	1	2	1	1
Espino-Salinas [63]	2022	1	1	1	1	2	1	1
Hsueh [70]	2021	1	1	1	1	1	1	1
Jakobsen [71]	2020	1	1	1	1	1	1	1
Zhao [72]	2019	1	1	1	1	2	1	1
Ku [74]	2018	1	1	1	1	1	1	1
Fadul [78]	2023	1	1	1	1	2	1	1
Auerbach [79]	2022	1	1	1	1	1	1	1
Otte Andersen [80]	2022	1	1	1	1	2	1	1
Opoku Asare [81]	2021	2	2	1	1	1	1	1
Chikersal [82]	2021	2	1	1	1	2	1	1
Zhang [83]	2021	1	1	1	1	1	1	1
Pedrelli [84]	2020	2	1	1	1	2	1	1
Mastoras [85]	2019	1	1	1	1	2	1	1
Saeb [86]	2015	2	1	1	1	2	1	1
Wiseman [87]	2025	1	1	1	1	1	1	1
Kim [90]	2023	1	1	1	1	1	1	1
Zhao [92]	2022	1	1	1	1	1	1	1
Ye [93]	2021	1	1	1	1	1	1	1
Klangpornkun [94]	2021	1	2	1	1	1	2	1
Demiroglu [95]	2020	1	1	1	1	1	1	1
Yamamoto [96]	2020	1	1	1	1	1	1	1
Choi [99]	2021	1	1	1	1	1	1	1
Anik [104]	2024	2	1	1	1	2	1	1
Tian [105]	2025	1	1	1	1	1	1	1
Islam [106]	2024	1	1	1	1	1	1	1
Makhmutova [107]	2022	1	1	1	1	1	1	1
Price [108]	2024	1	1	1	1	1	1	1
Griffiths [110]	2022	1	1	1	1	2	1	1
Rykov [113]	2021	2	1	1	1	2	1	1
Ahmed [119]	2022	2	1	1	1	2	1	1
Choi [120]	2022	2	1	1	1	2	1	1
Mahendran [121]	2019	2	1	1	1	2	1	1
Xu [122]	2019	2	1	1	1	2	1	1
Lu [123]	2018	2	1	1	1	2	1	1
Farhan [124]	2016	1	1	1	1	2	1	1
Karimi [125]	2025	1	1	1	1	1	1	1
Jacobson [126]	2020	2	1	1	1	2	1	1
Wang [127]	2018	1	1	1	1	2	1	1
Zhou [129]	2022	1	1	1	1	1	1	1
Williamson [130]	2019	1	1	1	1	2	1	1
Asare [132]	2022	1	1	1	1	2	1	1
Narziev [133]	2020	1	1	1	1	2	1	1
Kim [134]	2019	1	1	1	1	2	1	1
Di Matteo [137]	2021	1	1	1	1	1	1	1
Sverdlov [138]	2021	1	1	1	1	1	1	1
Minaeva [140]	2020	1	1	1	1	1	1	1
Dai [141]	2022	1	1	1	1	1	1	1
Tazawa [142]	2020	1	1	1	1	1	1	1
Thati [143]	2023	2	1	1	1	2	1	1
Jiang [144]	2024	1	1	1	1	1	1	1
Bai [148]	2021	2	1	1	1	2	1	1
Borelli [149]	2025	1	1	1	1	1	1	1
Chen [150]	2024	1	1	1	1	1	1	1

a QUADAS-2: Quality Assessment of Diagnostic Accuracy Studies-2.

b 1: low risk; 2: high risk.

### Results of Individual Studies and Syntheses

**Figure 2.**
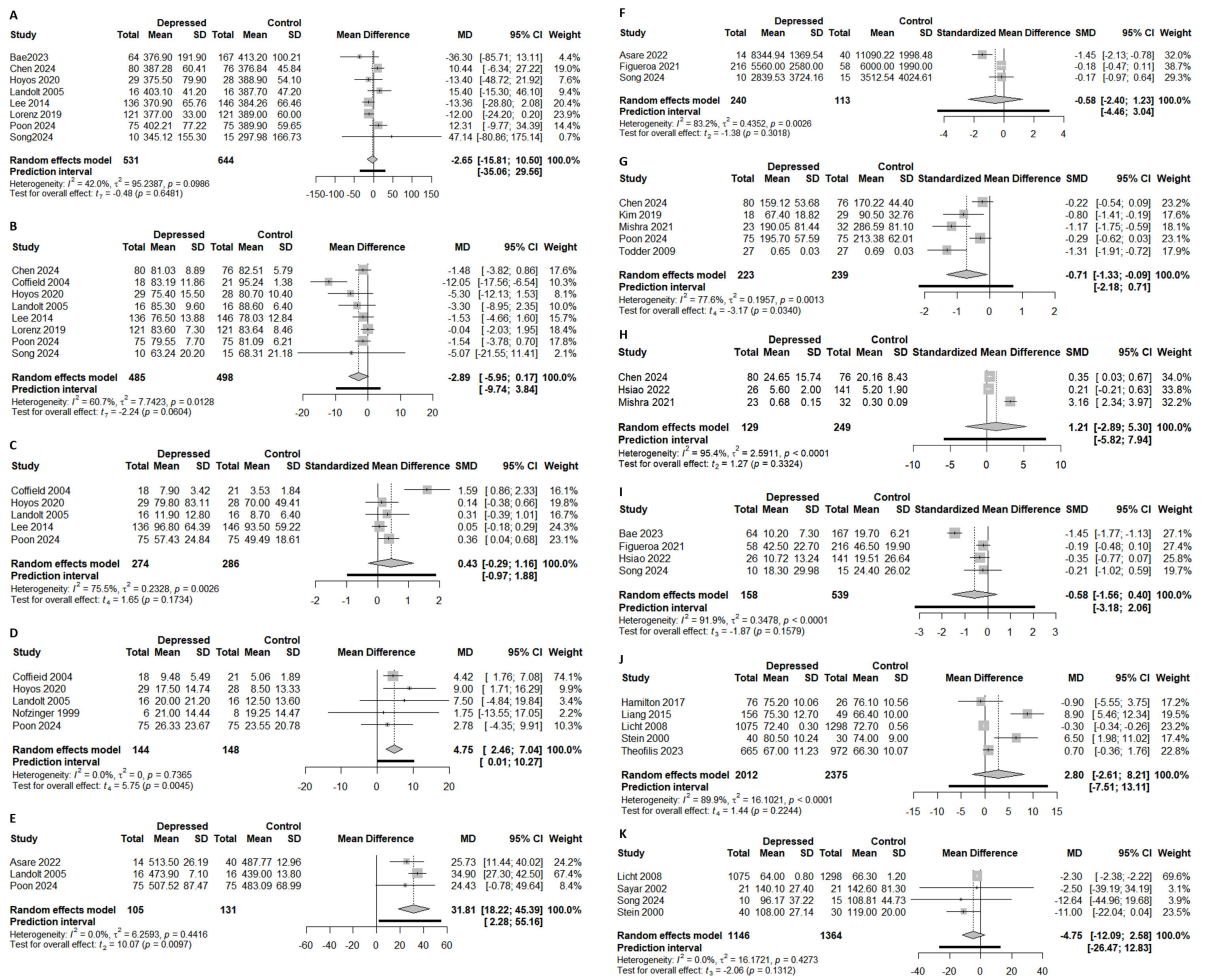
Forest plots of sleep-related parameters (A: total sleep time [20,33,109,115-117,147,150]; B: sleep efficiency [20,33,39,115-117,147,150]; C: wake after sleep onset [20,33,39,115,116]; D: sleep onset latency [20,39,40,115,116]; E: time in bed [20,115,132]), physical activity–related parameters (F: step counts [69,132,147]; G: physical activity counts [20,76,111,134,150]; H: sedentary time [64,111,150]; I: moderate-to-vigorous activity [64,69,109,147]), and cardiac parameters (J: mean heart rate [43,52,54,58,61]; K: SD of normal-to-normal intervals [58,61,114,147]). Pooled estimates have been calculated using a random-effects model with the Hartung-Knapp-Sidik-Jonkman adjustment. The between-study variance (𝜏^2^) has been estimated via the restricted maximum-likelihood method. 95% prediction intervals (PIs) have been derived using the Nagashima parametric bootstrap method.

Meta-analysis results are presented in Figure 2. A detailed overview of the parameters that could not be quantitatively synthesized and their reported associations with depression is provided in Multimedia Appendix 6.

#### Sleep-Related Parameters and Depression

##### Total Sleep Time

Total sleep time (TST) showed no significant difference between the depression and control groups (−2.65 min, 95% CI −15.81 to 10.50; *P*=.65; 95% PI −35.06 to 29.56; Figure 2A). Some studies suggested an association between TST and depressive symptoms [109,112,131,135], with TST identified as the most predictive variable in 21% of patients in a predictive model [131]. Treatment studies also found a negative correlation between reduced depressive symptoms and longer TST [112]. Conversely, other studies [4,20,22,36,41,115,132,136,147,150], including those focusing on older adults and medication-treated patients [26,116], reported no significant differences.

##### Sleep Efficiency

Sleep efficiency (SE) was lower in the depression group but not statistically significant (−2.89%, 95% CI −5.95 to 0.17; *P*=.06; 95% PI −9.74 to 3.84; Figure 2B). Several studies reported an improvement in SE with symptom reduction [35,39] and negative correlations across diverse populations [24,26,38,148]. However, some studies found no significant differences [20,115-117,147,150] or considered SE to be a nonsignificant predictor after adjustment [36,37]. Overall, SE tended to be lower in patients with depression, although the significance varied according to the analytical method.

##### Wake After Sleep Onset* *

Wake after sleep onset (WASO) tended to be longer in the depression group, though the pooled effect was not statistically significant (SMD 0.43, 95% CI −0.29 to 1.16; *P=*.17; 95% PI −0.97 to 1.88; Figure 2C). Previous evidence has been mixed: several studies reported prolonged WASO in the depression group [20,39,132], with 1 showing posttreatment improvement [34], while others found no consistent differences [33,115,116].

##### Sleep Fragmentation

Sleep fragmentation and frequent awakenings were identified as predictors of depression [24,39,110].

##### Sleep Onset Latency

Sleep onset latency (SOL) was significantly longer in the depression group (4.75 min, 95% CI 2.46-7.04, *P*=.005; 95% PI 0.01-10.27; Figure 2D). Most studies supported this trend [35,36,116], although some reported no significant differences in the adjusted models or after symptom resolution [34,39]. Overall, a prolonged SOL was consistently associated with depressive symptoms.

##### Time in Bed

Time in bed (TIB) was significantly longer in the depression group (31.81 min, 95% CI 18.22-45.39; *P*=.01; 95% PI 2.28-55.16; Figure 2E). Most studies corroborated this finding, with linear mixed models consistently associating longer TIB with depressive symptoms [26,115,132,145]. Although the 95% PI remained entirely above zero, its wide range suggests that the extent of TIB prolongation may vary substantially across individuals or settings.

##### REM Sleep and REM Latency

Most studies on REM sleep relied on small hospital-based samples, limiting generalizability. One study found that REM sleep was significantly reduced in treated patients, particularly during the first third of the night [41]. While some research identified REM sleep as a potential predictor of depression [110], others found no group differences [115,116] or explanatory value [26,31]. Findings on REM latency were also inconsistent; 1 study showed that antidepressant-treated patients had increased REM latency [41], whereas 2 studies found no difference between the depression and control groups [33,115]. In older adults, REM latency was reported to be comparable or longer [116]. Longitudinal analyses suggested that SOL may serve as a more reliable marker than REM latency [26].

##### Non–Rapid Eye Movement Sleep and SWS

Evidence on non–rapid eye movement (NREM) and SWS remains limited. While 1 study found a significant association between NREM sleep and depression [26], 2 studies reported no such relationship [41,116]. Similarly, no studies to date have identified a significant association between SWS and depression [33,41,115,116].

##### Sleep Onset, Midpoint, and Offset

Findings regarding sleep timing (onset, midpoint, and offset) varied. Several studies, particularly those using actigraphy, reported delayed timing in individuals with depression [23,112,113,116,150]. For instance, a study of hospitalized patients showed a weak negative correlation between sleep onset time and depression (*r*=−0.381), indicating a delay before discharge [112]. Other research in working adults and middle-aged women found later sleep midpoints and offsets associated with higher depression scores [23,113,116]. However, these results remain inconsistent across measurement methods and study populations [26,116,127,136,148].

### Physical Activity Parameters and Depression

#### Step Counts

The meta-analysis showed no significant difference in daily step counts between the depression and control groups (SMD −0.58, 95% CI −2.40 to 1.23; *P*=.30; 95% PI −4.46 to 3.04; Figure 2F), although the point estimate suggested fewer steps for the depression group. Longitudinal studies indicated that higher step counts were associated with reduced depression severity, with counts tending to increase during recovery [65,142]. In older adults, more daily steps were associated with a lower risk of future depressive symptoms [63]. However, some studies reported a limited predictive value of step counts [145,147].

#### Physical Activity Counts

Physical activity counts were lower in the depression group (SMD −0.71, 95% CI −1.33 to −0.09; *P*=.03; 95% PI −2.18 to 0.71; Figure 2G). This finding is consistent with actigraphy-based studies reporting reduced activity levels in individuals with depression [4,38,63,71,149,150]. Strong negative correlations with symptom severity were reported, particularly in hospitalized patients [112]. Longitudinal analyses showed that activity counts decreased during depressive episodes and increased with symptom improvement, supporting their relevance as state-dependent markers [4]. Notably, daytime activity levels were sensitive to clinical improvement, whereas nighttime activity measures did not reflect symptom changes [76]. Despite these promising results, the wide PI encompassing zero underscores the need for standardized approaches to account for potential inconsistencies in future observations.

#### Sedentary Time

Sedentary time showed no significant difference between the depression and control groups (SMD 1.21, 95% CI −2.89 to 5.30; *P*=0.33; 95% PI −5.82 to 7.94; Figure 2H). Findings regarding sedentary time were inconsistent. While some studies linked increased sedentary time to depression [66], others found no association after adjusting for moderate-to-vigorous physical activity (MVPA) or overall activity levels [74,135,149]. Longitudinal and intervention-based analyses further suggested that sedentary time alone is a relatively weak and nonspecific marker of depressive symptoms compared with mobility-related indicators such as activity counts or homestay [136]. This limitation may be particularly evident in older populations, where prolonged sedentary time may occur regardless of the depressive status [64,111].

#### MVPA Findings

The meta-analysis showed no significant difference in MVPA between the depression and control groups (SMD −0.58, 95% CI −1.56 to 0.40; *P*=.16; 95% PI −3.18 to 2.06; Figure 2I). However, individual studies suggested a possible link, with higher MVPA associated with reduced depression severity [68], particularly in older adults [109]. Notably, 1 study observed significant reductions in MVPA on weekends among individuals with depression [69].

#### LPA Findings

The results of LPA were mixed. Some studies reported that a greater time spent in LPA was associated with fewer depressive symptoms [68,74], whereas others found no significant association [110,111,123,147,149]. Overall, LPA may be more effective when interpreted alongside MVPA or step data.

#### Energy Expenditure

Energy expenditure, measured in kilocalories or metabolic equivalents of tasks (METs), has shown mixed findings. One study reported significantly lower energy expenditure in patients with depression than in controls [132], and a longitudinal study found increases in energy expenditure with symptom improvement [142]. Conversely, another study observed no group differences [117]. Further studies are required to assess its value as a biomarker.

### Cardiac Parameters and Depression

#### Time Domain

##### Mean Heart Rate

The meta-analysis showed a higher mean heart rate (mHR) during depression, although it was not statistically significant (2.80 beats per min, 95% CI −2.61 to 8.21; *P*=.22; 95% PI −7.51 to 13.11; Figure 2J). Notably, elevated nighttime mHR was linked to depression severity, whereas daytime mHR showed no consistent association [44,113].

##### SD of Normal-to-Normal Intervals

The SD of normal-to-normal intervals (SDNN) was lower in the depression group, but the meta-analysis result was not significant (−4.75 ms, 95% CI −12.09 to 2.58; *P*=.13; 95% PI −26.47 to 12.83; Figure 2K). While some studies reported significance [54,61,125], others did not [49,56]. Recent wearable electrocardiogram (ECG) research suggests that the SDNN tends to be reduced in individuals at risk of depression, reflecting autonomic dysregulation; however, its standalone discriminative power may be limited compared with other short-term HRV indices [125].

##### RMSSD Findings

The RMSSD tended to be lower in the depression group but was not consistently significant across studies [49,56,61,114]. Although some research on first-episode patients reported a significant association [54], others found no correlation between depression scores and the RMSSD [45,47,57,145]. Recent digital phenotyping studies using wearable ECG or multimodal sensing reported reduced RMSSD-related features in high-risk individuals, though their utility was often context-dependent or enhanced when combined with other features [125,147,149].

##### Proportion of Normal-to-Normal Intervals

Most studies showed no significant group differences in the proportion of normal-to-normal intervals (pNN) [43,54,112], although this has been highlighted in diagnostic models [53,55]. Wearable ECG-based screening studies similarly reported that pNN-related features contributed modestly to multivariate models but showed limited standalone discriminative ability for depression [125].

##### Mean RR Interval

The findings for the mean RR interval (RRI) were mixed. Two studies reported shorter RRIs in patients with severe depression [45,54], while 2 other studies found no relationship [56,59]. The mean RRI tended to be shorter in patients with depression, suggesting an autonomic imbalance; however, the small number of available studies (2/4, 50%) limits the generalizability of the findings [45,54], highlighting the need for further large-scale research.

##### SD of Heart Rate

Studies reported mixed findings regarding the SD of heart rate (HR). While some studies found that a reduced SD of HR was associated with greater depression severity [44], others showed an opposite association or a nonsignificant association [113,131].

### Frequency Domain

#### HF Power

HF power is generally lower in individuals with depression [43,45,49,53,56,59,61] and may predict future depressive symptoms [49,51,147]. However, some studies reported nonsignificant associations in multivariate models [54,57,61]. A recent wearable ECG study suggested that while HF-related parasympathetic indices contribute to depression classification models, they may lose independent significance when integrated with other HRV features [125].

#### LF Power

LF power has shown inconsistent results. While several studies reported lower LF in depression [45,49,51,59,61], others found higher values or no significant associations [54,56,57]. Wearable ECG-based studies indicated that LF-related features contribute variably to depression classification models, reflecting substantial heterogeneity and limited standalone interpretability [125].

#### LF/HF Ratio

Some studies reported a higher LF/HF ratio in the depression group [45,47,53,54], whereas others found no significant associations after adjustment [56,57,61]. Digital phenotyping evidence suggests that while the LF/HF ratio may reflect altered autonomic balance, its discriminative performance remains inconsistent across populations and analytic models [125,147]. Overall, the LF/HF ratio may show an increasing trend with depression but requires further validation as a reliable biomarker.

#### Very Low Frequency and Ultra-Low Frequency

Very low frequency (VLF) power was generally lower in individuals with depression [51,57,59,61] and was identified as a key feature in diagnostic models [57], although some studies reported nonsignificant associations after adjustment [49]. Ultra-low frequency (ULF) power also tended to decrease with greater depression severity [51,61]; however, 1 study found no significant difference between the depression and control groups [59]. This limited evidence warrants further investigation.

#### Total Power

Total power findings were inconsistent across studies. While 1 study reported significant effects on depression severity [54], other studies found no such differences [49,56,61]. However, its utility as a standalone biomarker is not well-supported.

### Smartphone Parameters and Depression

#### Phone Usage Frequency

Findings regarding usage frequency were mixed. One study reported higher use in individuals with depression, particularly students [86], while other studies found no such association [80,145]. Multimodal studies suggested that raw usage volume may not differ between groups; instead, temporal patterns, screen time, and communication regularity appear more relevant [4,81,138,149]. Furthermore, physical activity and sleep features often outperformed usage frequency in predictive models [84,133,137].

#### Phone Usage Duration

Results regarding phone usage duration were conflicting. While some studies observed longer usage in individuals with depression [86,122,127], others reported a shorter duration [132] or no differences [145]. Its predictive value appeared more pronounced in younger populations [122] but generally remained inferior to physiological indicators, such as HRV or physical activity [148,149]. These demographic variations and the relative inconsistency across findings limit its potential as a reliable biomarker.

#### Phone Calls

The frequency of phone calls was lower in some individuals with depression [138], though this effect was modest and highly dependent on age and social context [4,149]. Younger users preferred text-based communication over traditional calls [82,84,133]. Consequently, call frequency was insufficient as a biomarker but may complement indicators, such as activity or sleep [4,131,145,149].

#### Light Exposure

Low light exposure was shown to be linked to relapse [135] or depression in older adults [134], yet other studies reported no effects [133,137,142]. While potentially relevant for specific subgroups, this parameter requires further validation.

#### Number of Bluetooth-Connected Devices

Used as a proxy for social contact, this parameter was shown to correlate negatively with depression [146] and was integrated into predictive models [82,122]. While useful for assessing social activity, broader application requires more robust evidence.

#### Typing Patterns

Typing behavior analyzed via machine learning showed high accuracy in predicting depression [78,86], though further validation is needed to establish its reliability as a biomarker.

### Speech Parameters and Depression

Speech parameters were categorized into speech flow and voice acoustic parameters.

#### Speech Flow Parameters

##### Speech Rate

Slower speech rates are consistently associated with greater depression severity [88,96], with recent evidence confirming them as robust indicators of major depressive disorder (MDD) that correlate with objective executive dysfunction [87,144]. Models incorporating speech rate and duration outperformed acoustic-only models [95], though further validation across diverse populations is needed.

##### Speech Duration

Shorter speaking times were linked to higher depression severity [95,127,137,139,146]. Recent automated assessments and smartphone-derived data identified reduced active speaking time as a primary predictor of depression [137,144,150], establishing speech duration as a potential digital biomarker.

##### Pause Time

While some associations between depression and pause duration were nonsignificant [129], the majority of studies reinforced that increased pausing effectively captures psychomotor retardation and contributes to high-accuracy multimodal detection [87,88,96,144]. These findings underscore the potential of pause-related features as a reliable objective marker.

### Voice Acoustic Parameters

#### Mel-Frequency Cepstral Coefficients

Mel-frequency cepstral coefficients were significantly associated with the severity of depression in most studies [91-93,129]. Recent multimodal analyses further confirmed their effectiveness when integrated with facial and cardiovascular patterns [144], although a study found text-based features more predictive than mel-frequency cepstral coefficients [95].

#### Fundamental Frequency

Findings on fundamental frequency were mixed. Some studies reported significant differences or enhanced effectiveness through multimodal integration [92,93,129,144], whereas others found no significant associations [89,91], necessitating further clarification of its role.

#### Jitter

Jitter was significantly higher in some individuals with depression [89], but other studies did not identify it as a significant variable [95,129]. Recent evidence suggests that it provides discriminative value when integrated into multimodal frameworks [144], warranting further investigation.

#### Shimmer

Shimmer showed significant associations with depression in some studies [89,129], while others found no such link [95]. Its predictive power was notably enhanced within multimodal frameworks, contributing to more robust detection than when used alone [144].

### GPS Parameters and Depression

#### Total Distance

Most studies found a negative correlation between depression and total distance traveled [4,79,123,132,139,141,146]. Although not always statistically significant [86,124,145], reduced mobility was a promising digital biomarker, particularly when integrated into multimodal detection models [149].

#### Location Variance

Lower location variance was consistently associated with higher depression severity [4,82,86,124,132,145]. While 1 study noted inconsistencies depending on smartphone types [123], location variance remains a key feature in high-accuracy multimodal models for detecting depressive symptoms [149].

#### Time Spent at Home

A significant positive correlation exists between depression severity and time spent at home [4,86,123,124,127,132,149]. Notably, early changes in “homestay” were identified as a critical predictor of symptom improvement [136], demonstrating strong potential for longitudinal depression monitoring.

#### Location Entropy and Normalized Location Entropy

Reduced location and normalized entropy were generally associated with higher depression scores [4,86,123,124,132,149], reflecting less diverse movements. However, some studies found no significant associations [127,145].

#### Number of Locations Visited

Fewer visited locations correlated negatively with depression in most studies [4,123,127,132,137,149], though 1 study found no significant correlation [86].

#### Time Spent Moving

While associations between time spent moving and depression were inconsistent [86,123,124,145], recent longitudinal data identified early changes in moving time as a key predictor of symptom improvement [136]. These features are considered essential in multimodal frameworks for depression detection [149].

#### Average Moving Speed

Average moving speed was identified as a key feature in a depression prediction model [4,126], although its correlation often varied by device type [123]. While its standalone predictive power may be limited, it remains a crucial component in high-accuracy multimodal frameworks [149].

### Circadian Rhythm Parameters and Depression

#### Interdaily Stability

Most studies found no significant group differences [97,99,100,117,118,150], whereas 2 studies reported lower interdaily stability and greater depression severity [103,113]. While interdaily stability may reflect irregular daily routines, current evidence is limited.

#### Intradaily Variability

Generally, intradaily variability was found to be unrelated to depression [97,99,100,117,118,150], despite 1 study linking higher intradaily variability to greater severity [103]. Its utility as a standalone biomarker remains restricted.

#### Midline Estimating Statistic of Rhythm

Midline estimating statistic of rhythm was lower in individuals with greater depression severity [102,148] and was identified as an important predictor in other models [99,101]. Consistently, recent evidence indicates that patients with MDD exhibit significantly lower midline estimating statistic of rhythm than controls [150], potentially reflecting reduced energy levels, though some studies found no associations [97,140].

#### Amplitude, Acrophase, and Relative Amplitude

Lower amplitude was observed in the depression group in some studies [101,102], while others found no significant differences [97,99,140,148,150]. Regarding acrophase, most studies found no association [97,101,102,113,148], but a recent study noted a significantly later acrophase in MDD, suggesting a delayed circadian phase [150]. Relative amplitude was generally lower in individuals with depression, suggesting flatter activity cycles, although these findings often did not reach statistical significance [97,99,100,117].

#### Pseudo F-Statistic

The pseudo F-statistic (F-pseudo), which measures the circadian rhythm strength, was lower in individuals with depressive symptoms [101,102], suggesting weaker or irregular rhythms. However, other studies reported no significant associations [99,113].

#### Most Active 10-Hour Period and Least Active 5-Hour Period

The most active 10-hour period and the least active 5-hour period showed no significant differences between the depression and control groups across multiple studies [97,99,117]. These parameters currently provide no evidence of reliability as biomarkers for depression.

### Reporting Bias and Certainty of Evidence

The qualitative assessment of reporting bias suggested a low likelihood of missing relevant studies, supported by comprehensive multidatabase searches and manual reference screening. According to the GRADE approach, the certainty of evidence ranged from low to very low across the key digital biomarkers (Table 2). The certainty was low for SOL, TIB, and physical activity counts, whereas it was very low for TST, SE, WASO, and mHR. The overall certainty was mainly downgraded due to inconsistency across studies and imprecision associated with wide CIs or PIs.

**Table 2.** GRADE (Grading of Recommendations, Assessment, Development and Evaluation) summary of findings for key digital biomarkers in individuals with depression.

Certainty assessment	Studies, n	Study design	Risk of bias	Inconsistency	Indirectness	Imprecision	Other considerations	Individuals with depression, n	Controls (no depression), n	Effect, relative (95% CI)	Effect, absolute (95% CI)	Certainty	Importance
Total sleep time	8	Nonrandomized studies	Not serious	Serious^a^	Not serious	Serious^b^	None	531	644	—^c^	MD^d^ 2.65 min fewer (15.81 fewer to 10.5 more)	Very low^a^^,^^b^	Critical
Sleep efficiency	8	Nonrandomized studies	Not serious	Serious^e^	Not serious	Serious^b^	None	485	498	—	MD 2.89% lower (5.95 lower to 0.17 higher)	Very low^b^^,^^e^	Critical
Wake after sleep onset	5	Nonrandomized studies	Not serious	Serious^e^	Not serious	Serious^b^	None	274	286	—	SMD^f^ 0.43 SD more (0.29 fewer to 1.16 more)	Very low^b^^,^^e^	Critical
Sleep onset latency	5	Nonrandomized studies	Not serious	Not serious	Not serious	Not serious	None	144	148	—	MD 4.75 min more (2.46 more to 7.04 more)	Low	Critical
Time in bed	3	Nonrandomized studies	Not serious	Not serious	Not serious	Not serious	None	105	131	—	MD 31.81 min more (18.22 more to 45.39 more)	Low	Critical
Physical activity counts	5	Nonrandomized studies	Not serious	Not serious	Not serious	Not serious	None	223	239	—	SMD 0.71 SD lower (1.33 lower to 0.09 lower)	Low	Critical
Mean heart rate	5	Nonrandomized studies	Not serious	Serious^e^	Not serious	Serious^b^	None	2012	2375	—	MD 2.8 bpm higher (2.61 lower to 8.21 higher)	Very low^b^^,^^e^	Critical

a Downgraded for serious inconsistency due to conflicting findings across studies and a wide prediction interval.

b Downgraded for serious imprecision due to a wide CI crossing the null effect.

c Not applicable.

d MD: mean difference.

e Downgraded for serious inconsistency due to moderate heterogeneity and variable results across studies.

f SMD: standardized mean difference.

## Discussion

### Principal Findings

This systematic review synthesized digital biomarkers for depression across diverse domains—sleep, physical activity, cardiac parameters, smartphone usage, speech, GPS data, and circadian rhythms—to identify more consistent indicators across multiple digital signals. Our meta-analysis identified prolonged SOL, increased TIB, and reduced activity counts as the most consistent behavioral features associated with depression. These findings support the hypothesis that digital phenotyping can capture objective manifestations of depression, particularly sleep initiation difficulties and reduced energy expenditure, which are often difficult to quantify through traditional self-reports.

A key contribution of this systematic review is the application of quantitative meta-analyses in a field where such synthesis was previously considered methodologically challenging [10,11]. Unlike prior meta-analyses that focused on single domains or summarized findings narratively because of methodological heterogeneity [13,14], this review systematically evaluates digital biomarkers across diverse domains. Consistent with earlier reports, the results indicate that depression is not characterized by a single physiological signature but rather by a constellation of behavioral and biological changes [6,149].

Furthermore, unlike recent scoping reviews on general mental illness [151], our analysis is specifically tailored to MDD. Importantly, the interpretation of these findings must consider the substantial heterogeneity observed across studies. The CIs reflect the average effect across the included studies, whereas the PIs indicate the range of effects that may occur in future settings. For several outcomes, wide PIs crossing the null value suggest that effect sizes may vary considerably depending on the study population, device type, measurement protocol, and analytical approach. These findings indicate that group-level average effects may not generalize consistently across contexts, supporting the use of personalized, longitudinal multibiomarker models for effective monitoring and intervention.

### Key Digital Biomarkers

Several biomarkers emerged as important indicators of depression across multiple domains. In the sleep domain, individuals with depression showed significantly longer SOL and increased TIB compared to controls without depression. These findings suggest that the sleep-wake cycle in MDD is characterized more by structural fragmentation than by simple reductions in sleep duration. Specifically, increased TIB likely reflects hallmark symptoms such as psychomotor retardation and lethargy. While polysomnography remains the gold standard [152], these results demonstrate that wearable devices can capture such patterns in ecologically valid, naturalistic settings.

The absence of significant differences in TST, SE, and WASO, which are parameters frequently associated with depression in previous reviews [4], highlights the clinical heterogeneity of MDD [153,154]. Depression may manifest as either insomnia or hypersomnia, depending on the subtype, potentially neutralizing average effects in pooled analyses [155]. The wide PIs further indicate substantial heterogeneity, likely driven by differences in study populations, medication use, and device-specific scoring. These findings underscore that absolute sleep quantity alone is an insufficient marker, and the relationship between sleep and depression is shaped by complex biopsychosocial factors [153,154]. Consequently, temporal and qualitative features, such as SOL and TIB, may serve as more clinically informative indicators than total sleep volume [9,156].

With respect to sleep architecture, evidence regarding REM-related parameters remains inconclusive. While some studies suggest that shortened REM latency may precede depression [157], results for REM duration and frequency are inconsistent [26,31,41,115,116]. Age-related changes [33] and reliance on single-night laboratory measurements further limit generalizability. These findings highlight the need for longitudinal, real-world assessments to clarify the relationship between sleep architecture and depression.

Physical activity counts emerged as a sensitive state-dependent marker, showing significant reductions in depression groups. Compared with simple step counts, activity counts better capture movement intensity and frequency [65,142,158], reflecting the energy deficits associated with MDD. Although daily step counts were not significantly different in pooled analyses, longitudinal evidence suggests that they may be sensitive to individual recovery trajectories [65,142]. Several studies also reported more pronounced reductions during daytime hours and on weekends, emphasizing the importance of temporal patterns rather than simple daily averages [158,159]. In contrast, MVPA, sedentary time, and LPA showed inconsistent results. This lack of robust significance likely reflects high heterogeneity and the limited specificity of these markers when used in isolation.

GPS-derived parameters, including total distance traveled, location entropy, time spent at home, and number of locations visited, capture behavioral and environmental changes associated with depression. Mobility patterns, such as reduced diversity in visited locations (entropy) and increased homestay durations, have emerged as promising indicators of depressive symptoms [160]. Recent longitudinal evidence suggests that early changes in homestay and mobility patterns may predict symptom improvement [136], highlighting the value of GPS-derived features in multimodal monitoring frameworks [149].

Cardiac parameters showed less consistent results. Individuals with depression tended to exhibit higher nocturnal mHR and lower HRV, although these differences were not statistically significant in pooled analyses. These trends may reflect physiological hyperarousal and autonomic dysregulation [161,162], but their effects appear to vary across populations and study conditions. Accordingly, these markers may be more informative when interpreted within multimodal models that account for age, medication use, and comorbid conditions. Despite this overall inconsistency, short-term HRV indices and frequency-domain features (eg, HF and VLF) remain meaningful components in diagnostic and multimodal frameworks, particularly as sensitive indicators of autonomic imbalance in first-episode depression or high-risk individuals [54,125,149].

Speech parameters, including slower speech rates and longer pauses, consistently distinguished individuals with MDD from controls in several studies [87,144]. These acoustic features likely reflect psychomotor retardation.

### Implications for Personalized Digital Phenotyping

Digital biomarkers offer several advantages over traditional self-reported measures by providing objective, continuous data that reduce recall bias and capture early physiological and behavioral changes [163,164]. Passive monitoring enables large-scale, low-burden data collection, supporting early detection and personalized, data-driven interventions [5,8,165].

However, the substantial variability observed across individuals highlights the need to interpret these signals within a personalized framework, as traditional group-based models may be inadequate in many contexts [166]. The predictive power of biomarkers varies with age. TIB, daily step counts, and MVPA are more predictive in older adults [11,109,167], whereas smartphone usage patterns show stronger associations in younger individuals [11,122]. High-resolution temporal data, such as nighttime HRV or weekend reductions in MVPA, may provide additional insights into symptom dynamics, underscoring the importance of temporal patterns in digital measurements [69]. Monitoring sleep-related biomarkers in relation to changes in individual symptoms may further improve the prediction and tracking of depressive episodes [6]. Together, these findings suggest that digital monitoring strategies should focus on detecting deviations from an individual’s baseline rather than relying on universal thresholds.

### Methodological Limitations of the Included Studies and Evidence Certainty

Despite identifying several robust biomarkers, parameters, such as LPA, sedentary time, and specific cardiac measures, showed inconsistent results. This inconsistency, together with the wide PIs observed across studies, likely reflects not only diverse device technologies and measurement protocols, but also the symptomatic variability across depression subtypes [155,168]. While wearable devices offer objective physiological data, they do not directly capture emotional states [169]. Furthermore, the predominance of cross-sectional designs limits our ability to determine the temporal or causal relationships between these digital signals and depressive symptoms [170].

Risk-of-bias assessments also indicated several methodological concerns. Although participation rates were somewhat low in certain studies [24,34,59,62,128], they were generally acceptable across the majority of the included research [4,25,27,31,32,35,38,42,45,49,52-54,56,60,67,68,76,89,91,101,111,112,114,115,117,131,145]. However, comparisons between participants and nonparticipants were often not reported, raising the possibility of selection bias. For example, individuals with more severe depressive symptoms, lower motivation, or limited familiarity with wearable technologies may have been less likely to participate or remain in the studies. In addition, adjustment for confounding variables was limited in several studies. For diagnostic accuracy studies, most domains showed low risk of bias, but concerns regarding applicability were common because study populations did not always reflect real-world clinical settings.

Consistent with these methodological limitations, the GRADE assessment indicated low to very low certainty of evidence for the key digital biomarkers. This was primarily due to inconsistency across studies and imprecision associated with wide CIs and PIs. These findings suggest that the pooled estimates should be interpreted cautiously and that further well-designed, standardized studies are needed to strengthen the evidence base.

### Limitations of This Review

This systematic review has several limitations. First, substantial heterogeneity across studies limited the precision and generalizability of the pooled estimates, reflecting differences in devices, study populations, monitoring periods, and analytic methods. Second, the overall certainty of evidence was low to very low according to the GRADE framework, which reduces confidence in the pooled estimates. Third, a formal statistical assessment of reporting bias was not feasible because fewer than 10 studies were available for each meta-analysis. Finally, many studies included nonclinical or convenience samples, which may limit the generalizability of the findings to real-world clinical populations.

### Future Directions for Digital Biomarker Research

Future research should move beyond the identification of individual markers toward the development of integrated, clinically actionable digital phenotyping systems. The consistent associations observed for indicators, such as SOL, TIB, and activity counts, suggest that certain behavioral signals may serve as foundational components of continuous mental health monitoring. The next phase of research should therefore focus on embedding these markers within longitudinal, multimodal frameworks that support personalized clinical decision-making and improve the precision of depression monitoring [149,150,155], with the potential to inform more proactive intervention strategies in real-world settings.

Achieving this transition will require greater methodological standardization across devices, measurement protocols, and analytic pipelines. Reducing reliance on proprietary, nontransparent algorithms and promoting device-agnostic, reproducible approaches will be essential for ensuring clinical validity and interoperability across health care systems. Integration of digital biomarker data into electronic health records may further enable real-time, context-aware decision support while minimizing additional cognitive burden on clinicians.

More broadly, digital biomarkers may support earlier detection and more adaptive treatment strategies by providing high-frequency, objective data on symptom trajectories [136,164]. At the population level, scalable signals such as physical activity and mobility patterns may also facilitate screening and risk stratification in settings with limited access to mental health professionals. Future work should therefore prioritize longitudinal, multimodal designs; representative clinical populations; standardized measurement protocols; and transparent reporting of analytic methods.

### Conclusion

This systematic review provides a comprehensive synthesis of multimodal digital biomarkers for depression. Unlike previous reviews that focused on single signals or technical feasibility, this systematic review advances the field by establishing a standardized framework for objective clinical decision-making through a rigorous meta-analysis. The findings indicate that while certain markers, particularly SOL and physical activity counts, show consistent average differences, their effects vary substantially across settings, as reflected by wide PIs. These results suggest that depression cannot be reliably characterized by a single digital biomarker. Instead, a multimodal, personalized approach that integrates physiological, behavioral, and contextual signals is likely to be more effective for real-world applications. More broadly, this systematic review demonstrates that quantitative synthesis in digital phenotyping is feasible despite substantial heterogeneity and that meaningful signals can be identified when methodological rigor and transparent reporting are applied. Establishing standardized, clinically interpretable digital biomarker frameworks will be essential for advancing objective, continuous, and personalized assessments of depression in routine care.

## Supplementary material

10.2196/76432 Multimedia Appendix 1 Search terms and strategy.

10.2196/76432 Multimedia Appendix 2 Detailed study characteristics and quality assessment of the included studies.

10.2196/76432 Multimedia Appendix 3 Participants and analytical methods in the studies.

10.2196/76432 Multimedia Appendix 4 Depression measurement tools included in the studies.

10.2196/76432 Multimedia Appendix 5 Digital biomarkers included in the studies.

10.2196/76432 Multimedia Appendix 6 Digital biomarkers not included in the meta-analysis.

10.2196/76432 Checklist 1 PRISMA checklist.

## Abbreviations

BDI: Beck Depression Inventory
CES-D: Center for Epidemiologic Studies Depression Scale
ECG: electrocardiogram
GDS: Geriatric Depression Scale
GRADE: Grading of Recommendations, Assessment, Development, and Evaluation
HDRS: Hamilton Depression Rating Scale
HF: high frequency
HKSJ: Hartung-Knapp-Sidik-Jonkman
HR: heart rate
HRV: heart rate variability
IDAS: Inventory of Depression and Anxiety Symptoms
IDS-SR: Inventory of Depressive Symptomatology
IRB: Institutional Review Board
LF: low frequency
LPA: light physical activity
MD: mean difference
MDD: major depressive disorder
MET: metabolic equivalent of task
mHR: mean heart rate
MVPA: moderate-to-vigorous physical activity
NREM: non–rapid eye movement
PHQ: Patient Health Questionnaire
pNN: proportion of normal-to-normal intervals
PRISMA: Preferred Reporting Items for Systematic Reviews and Meta-Analyses
QUADAS-2: Quality Assessment of Diagnostic Accuracy Studies-2
REM: rapid eye movement
RMSSD: root mean square of successive differences
RRI: RR interval
SDNN: SD of normal-to-normal intervals
SE: sleep efficiency
SGDS-K: shortened Korean version of the Geriatric Depression Scale
SIGN: Scottish Intercollegiate Guidelines Network
SMD: standardized mean difference
SOL: sleep onset latency
SWS: slow-wave sleep
TIB: time in bed
TST: total sleep time
ULF: ultra-low frequency
VLF: very low frequency
WASO: wake after sleep onset
