# Supplementary material for: The Role of Digital Biomarkers in Physiological Signal-Based Depression Assessment: Systematic Review and Meta-Analysis
Source: J Med Internet Res. 2026 Apr 2;28:e76432. doi: 10.2196/76432 (PMC13046098; doi:10.2196/76432)
Supplement: Multimedia Appendix 1 [file jmir-v28-e76432-s001.docx]

**Multimedia Appendix 1. Search terms and strategy.**

**Table S1. Search Terms and Databases for the Current Systematic Review on Digital Biomarkers for Depression Assessment**

| **Language** | **Search Terms** | | **Databases** |
| --- | --- | --- | --- |
| Korean | **P**atients | 우울 | KISS, RISS,  KMbase,  KoreaMed |
|  | **I**ntervention | 웨어러블, 기기, 애플리케이션, 어플리케이션, 어플, 앱, 스마트폰, 스마트 워치, 스마트 밴드, 모바일, 평가, 측정, 디지털 |  |
|  |  | 바이오마커, 혈압, 심박변이도, 심박수 변동성, 심박 변동성, 심박수, 심장박동, 심장박출지수, 일주기 리듬, 빛 노출, 휴대폰 사용, 휴대폰 사용량, 스크린 타임, 수면, 깨어난 시간, 삶의 규칙성, 음성, 목소리, 대화, 말하기, 움직임, 활동, 평균 활동량, 평균 소모 에너지, 스포츠, 운동, 걷기, 걸음, 앉기, 뇌파, 뇌전도, 심전도 |  |
| English | **P**atients | Depressi*, MDD | Cochrane Library, OVID-MEDLINE,  PsycINFO, CINAHL,  IEEE Xplore,  Web of Science |
|  | **I**ntervention | Phenotyp*, Pheno-typ*, Wearable, mobile application, App, Apps, Smart Phone*, Smartphone*, Smart Watch*, Android, iPhone, Mobile-phone, Quantitative measure*, Quantitative assessment*, Physiological marker*, Sensor*, Sensing, Digital, Passive, sens*, GPS, global positioning systems, Accelerome*, Accelerometer*, Handheld computer*, Wi-Fi, Actigraph*, Technolog*, Wristband*, Wrist band*, Wrist-worn, Wrist worn, fitbit, Track*, Pedometer* |  |
|  |  | Biomarker*, Blood Pressure, BP, heart rate*, HRV, RHR, light*, light exposure, Phone Usage, Screen Time, Sleep*, REM, Rapid eye movement, regularity of life, circadian rhythm*, acrophase, Awake, Speech, Pitch variability, Voice, Audio, Temperature, Movement*, Motion, Ambulat*, activity, activities, Sport*, Behavioral parameter*, Daily life behavior, Daily-life behavior, Exercise*, Walking, Stepping, Sitting, Sedentary, Supine position, EEG, Electroencephalogram, ECG, electrocardiogram, electrodermal, Geographic location* |  |

**Table S2. Search strategy for MEDLINE (via Ovid)**

| **Date searched:** December 28, 2025 | | |
| --- | --- | --- |
| **Total records retrieved:** 10,109 | | |
| **Search Query** | | |
| **P** | 1 | Depressi*.ab,ti. |
|  | 2 | MDD.ab,ti. |
| **I (method)** | 3 | Phenotyp*.ab,ti. |
|  | 4 | Pheno-typ*.ab,ti. |
|  | 5 | Wearable.ab,ti. |
|  | 6 | mobile application.ab,ti. |
|  | 7 | App.ab,ti. |
|  | 8 | Apps.ab,ti. |
|  | 9 | Smart Phone*.ab,ti. |
|  | 10 | Smartphone.ab,ti. |
|  | 11 | Smart Watch*.ab,ti. |
|  | 12 | Android.ab,ti. |
|  | 13 | iPhone.ab,ti. |
|  | 14 | Mobile phone.ab,ti. |
|  | 15 | Mobile-phone.ab,ti. |
|  | 16 | Quantitative measure.ab,ti. |
|  | 17 | Quantitative assessment*.ab,ti. |
|  | 18 | Objective measure*.ab,ti. |
|  | 19 | Objective assessment*.ab,ti. |
|  | 20 | Physiological marker*.ab,ti. |
|  | 21 | Sensor*.ab,ti. |
|  | 22 | Sensing.ab,ti. |
|  | 23 | Digital.ab,ti. |
|  | 24 | Passive sens*.ab,ti. |
|  | 25 | GPS.ab,ti. |
|  | 26 | global positioning systems.ab,ti. |
|  | 27 | Accelerometer*.ab,ti. |
|  | 28 | Handheld computer*.ab,ti. |
|  | 29 | Wi-Fi.ab,ti. |
|  | 30 | Actigraph*.ab,ti. |
|  | 31 | Technolog*.ab,ti. |
|  | 32 | Wristband*.ab,ti. |
|  | 33 | Wrist band*.ab,ti. |
|  | 34 | Wrist-worn.ab,ti. |
|  | 35 | Wrist worn.ab,ti. |
|  | 36 | Accelerome*.ab,ti. |
|  | 37 | fitbit.ab,ti. |
|  | 38 | Track*.ab,ti. |
|  | 39 | Pedometer*.ab,ti. |
| **I (biomarker)** | 40 | Biomarker*.ab,ti. |
|  | 41 | Blood Pressure.ab,ti. |
|  | 42 | BP.ab,ti. |
|  | 43 | heart rate*.ab,ti. |
|  | 44 | HRV.ab,ti. |
|  | 45 | RHR.ab,ti. |
|  | 46 | light*.ab,ti. |
|  | 47 | light exposure.ab,ti. |
|  | 48 | Phone Usage.ab,ti. |
|  | 49 | Screen Time.ab,ti. |
|  | 50 | Sleep*.ab,ti. |
|  | 51 | REM.ab,ti. |
|  | 52 | Rapid eye movement.ab,ti. |
|  | 53 | regularity of life.ab,ti. |
|  | 54 | circadian rhythm*.ab,ti. |
|  | 55 | acrophase.ab,ti. |
|  | 56 | Awake.ab,ti. |
|  | 57 | Speech.ab,ti. |
|  | 58 | Pitch variability.ab,ti. |
|  | 59 | Voice.ab,ti. |
|  | 60 | Audio.ab,ti. |
|  | 61 | Temperature.ab,ti. |
|  | 62 | Movement*.ab,ti. |
|  | 63 | Motion.ab,ti. |
|  | 64 | Ambulat*.ab,ti. |
|  | 65 | activity.ab,ti. |
|  | 66 | activities.ab,ti. |
|  | 67 | Sport*.ab,ti. |
|  | 68 | Behavioral parameter*.ab,ti. |
|  | 69 | Daily life behavior.ab,ti. |
|  | 70 | Daily-life behavior.ab,ti. |
|  | 71 | Exercise*.ab,ti. |
|  | 72 | Walking.ab,ti. |
|  | 73 | Stepping.ab,ti. |
|  | 74 | Sitting.ab,ti. |
|  | 75 | Sedentary.ab,ti. |
|  | 76 | Supine Position.ab,ti. |
|  | 77 | EEG.ab,ti. |
|  | 78 | Electroencephalogram.ab,ti. |
|  | 79 | EKG.ab,ti. |
|  | 80 | Elektrokardiogramm.ab,ti. |
|  | 81 | ECG.ab,ti. |
|  | 82 | electrocardiogram.ab,ti. |
|  | 83 | electrodermal.ab,ti. |
|  | 84 | Geographic location*.ab,ti. |
| **P_or** | 85 | 1 or 2 |
| **Im_or** | 86 | 3 or 4 or 5 or 6 or 7 or 8 or 9 or 10 or 11 or 12 or 13 or 14 or 15 or 16 or 17 or 18 or 19 or 20 or 21 or 22 or 23 or 24 or 25 or 26 or 27 or 28 or 29 or 30 or 31 or 32 or 33 or 34 or 35 or 36 or 37 or 38 or 39 |
| **Ib_or** | 87 | 40 or 41 or 42 or 43 or 44 or 45 or 46 or 47 or 48 or 49 or 50 or 51 or 52 or 53 or 54 or 55 or 56 or 57 or 58 or 59 or 60 or 61 or 62 or 63 or 64 or 65 or 66 or 67 or 68 or 69 or 70 or 71 or 72 or 73 or 74 or 75 or 76 or 77 or 78 or 79 or 80 or 81 or 82 or 83 or 84 |
| **P and Im and Ib** | 88 | 85 and 86 and 87 |

**Table S3. Search strategy for KOREAMED via Korean Association of Medical Journal Editors**

| **Date searched:** December 28, 2025 | |
| --- | --- |
| **Total records retrieved:** 95 | |
| **Search Query** | |
| 1 | (Depressi*[ALL] OR MDD[ALL]) AND (Phenotyp*[ALL] OR Pheno-typ*[ALL] OR Wearable[ALL] OR mobile application[ALL] OR App[ALL] OR Apps[ALL] OR Smart Phone*[ALL] OR Smartphone*[ALL] OR Smart Watch*[ALL] OR Android[ALL] OR iPhone[ALL] OR Mobile-phone[ALL] OR Quantitative measure*[ALL] OR Quantitative assessment*[ALL] OR Objective measure*[ALL] OR Objective assessment*[ALL] OR Sensor*[ALL] OR Sensing[ALL] OR Digital[ALL] OR Passive sens*[ALL] OR GPS[ALL] OR global positioning systems[ALL] OR Accelerometer*[ALL] OR Handheld computer*[ALL] OR Wi-Fi[ALL] OR Actigraph*[ALL] OR Technolog*[ALL] OR Wristband[ALL] OR Wrist band*[ALL] OR Wrist-worn[ALL] OR Accelerome*[ALL] OR fitbit[ALL] OR Track*[ALL] OR Pedometer*[ALL]) AND (Biomarker*[ALL] OR Blood Pressure[ALL] OR BP[ALL] OR heart rate*[ALL] OR HRV[ALL] OR RHR[ALL] OR light*[ALL] OR light exposure[ALL] OR Phone Usage[ALL] OR Screen Time[ALL] OR Sleep*[ALL] OR REM[ALL] OR Rapid eye movement[ALL] OR regularity of life[ALL] OR circadian rhythm*[ALL] OR acrophase[ALL] OR Awake[ALL] OR Speech[ALL] OR Pitch variability[ALL] OR Voice[ALL] OR Audio[ALL]) |
| 2 | (Depressi*[ALL] OR MDD[ALL]) AND (Phenotyp*[ALL] OR Pheno-typ*[ALL] OR Wearable[ALL] OR mobile application[ALL] OR App[ALL] OR Apps[ALL] OR Smart Phone*[ALL] OR Smartphone*[ALL] OR Smart Watch*[ALL] OR Android[ALL] OR iPhone[ALL] OR Mobile-phone[ALL] OR Quantitative measure*[ALL] OR Quantitative assessment*[ALL] OR Objective measure*[ALL] OR Objective assessment*[ALL] OR Sensor*[ALL] OR Sensing[ALL] OR Digital[ALL] OR Passive sens*[ALL] OR GPS[ALL] OR global positioning systems[ALL] OR Accelerometer*[ALL] OR Handheld computer*[ALL] OR Wi-Fi[ALL] OR Actigraph*[ALL] OR Technolog*[ALL] OR Wristband[ALL] OR Wrist band*[ALL] OR Wrist-worn[ALL] OR Accelerome*[ALL] OR fitbit[ALL] OR Track*[ALL] OR Pedometer*[ALL]) AND (Temperature[ALL] OR Movement*[ALL] OR Motion[ALL] OR Ambulat*[ALL] OR activity[ALL] OR activities[ALL] OR Sport*[ALL] OR Behavioral parameter*[ALL] OR Daily life behavior[ALL] OR Daily-life behavior[ALL] OR Exercise*[ALL] OR Walking[ALL] OR Stepping[ALL] OR Sitting[ALL] OR Sedentary[ALL] OR Supine Position[ALL] OR EEG[ALL] OR Electroencephalogram[ALL] OR EKG[ALL] OR Elektrokardiogramm[ALL] OR ECG[ALL] OR electrocardiogram[ALL] OR electrodermal[ALL] OR Geographic location*[ALL]) |
| 3 | 1 or 2 |

**Table S4. Search strategy for KMbase**

| **Date searched:** December 28, 2025 |
| --- |
| **Total records retrieved:** 623 |
| **Search Query** |
| (우울\|abstract) AND ((웨어러블\|abstract) OR (애플리케이션\|abstract) OR (어플\|abstract) OR (앱\|abstract) OR (스마트폰\|abstract) OR (스마트 워치\|abstract) OR (스마트 밴드\|abstract) OR (모바일\|abstract) OR (평가\|abstract) OR (측정\|abstract) OR (디지털\|abstract)) AND ((바이오마커\|abstract) OR (혈압\|abstract) OR (심박변이도\|abstract) OR (심박수 변동성\|abstract) OR (심박 변동성\|abstract) OR (심박수\|abstract) OR (심장박동\|abstract) OR (일주기 리듬\|abstract) OR (빛 노출\|abstract) OR (휴대폰 사용\|abstract) OR (스크린 타임\|abstract) OR (수면\|abstract) OR (음성\|abstract) OR (목소리\|abstract) OR (대화\|abstract) OR (말하기\|abstract) OR (움직임\|abstract) OR (활동\|abstract) OR (활동량\|abstract) OR (스포츠\|abstract) OR (운동\|abstract) OR (걷기\|abstract) OR (걸음\|abstract) OR (뇌파\|abstract) OR (뇌전도\|abstract) OR (심전도\|abstract)) |

**Table S5. Search strategy for IEEE Xplore**

| **Date searched:** December 28, 2025 |
| --- |
| **Total records retrieved:** 978 |
| **Search Query (abstract and title)** |
| (Depressi* OR MDD) AND (Phenotyp* OR Pheno-typ* OR Wearable OR mobile application OR App OR Apps OR Smart Phone* OR Smartphone* OR Smart Watch* OR Android OR iPhone OR Mobile phone OR Mobile-phone OR Quantitative measure* OR Quantitative assessment* OR Objective measure* OR Objective assessment*) AND (Biomarker* OR Blood Pressure OR BP OR heart rate* OR HRV OR RHR OR light* OR light exposure OR Phone Usage OR Screen Time OR Sleep* OR REM OR Rapid eye movement OR regularity of life OR circadian rhythm* OR acrophase OR Awake OR Speech OR Pitch variability) |
| (Depressi* OR MDD) AND (Phenotyp* OR Pheno-typ* OR Wearable OR mobile application OR App OR Apps OR Smart Phone* OR Smartphone* OR Smart Watch* OR Android OR iPhone OR Mobile phone OR Mobile-phone OR Quantitative measure* OR Quantitative assessment* OR Objective measure* OR Objective assessment*) AND (Voice OR Audio OR Temperature OR Movement* OR Motion OR Ambulat* OR activity OR activities OR Sport* OR Behavioral parameter* OR Daily life behavior OR Daily-life behavior OR Exercise* OR Walking OR Stepping OR Sitting OR Sedentary OR Supine Position OR EEG OR Electroencephalogram OR EKG OR Elektrokardiogramm OR ECG OR electrocardiogram OR electrodermal OR Geographic location*) |
| (Depressi* OR MDD) AND (Physiological marker* OR Sensor* OR Sensing OR Digital OR Passive sens* OR GPS OR global positioning systems OR Accelerometer* OR Handheld computer* OR Wi-Fi OR Actigraph* OR Technolog*) AND (Biomarker* OR Blood Pressure OR BP OR heart rate* OR HRV OR RHR OR light* OR light exposure OR Phone Usage OR Screen Time OR Sleep* OR REM OR Rapid eye movement OR regularity of life OR circadian rhythm* OR acrophase OR Awake OR Speech OR Pitch variability) |
| (Depressi* OR MDD) AND (Physiological marker* OR Sensor* OR Sensing OR Digital OR Passive sens* OR GPS OR global positioning systems OR Accelerometer* OR Handheld computer* OR Wi-Fi OR Actigraph* OR Technolog*) AND (Voice OR Audio OR Temperature OR Movement* OR Motion OR Ambulat* OR activity OR activities OR Sport* OR Behavioral parameter* OR Daily life behavior OR Daily-life behavior OR Exercise* OR Walking OR Stepping OR Sitting OR Sedentary OR Supine Position OR EEG OR Electroencephalogram OR EKG OR Elektrokardiogramm OR ECG OR electrocardiogram OR electrodermal OR Geographic location*) |

**Table S6. Search strategy for RISS**

| **Date searched:** December 28, 2025 | | |
| --- | --- | --- |
| **Total records retrieved:** 956 | | |
| **Search Query (abstract and title)** | | |
| 우울.Ti | 웨어러블\|기기\|애플리케이션\|어플리케이션\|어플\|앱\|스마트폰\|스마트 워치\|스마트 밴드\|모바일\|평가\|측정\|디지털 Ti. | 바이오마커\|혈압\|심박변이도\|심박수 변동성\|심박 변동성\|심박수\|심장박동\|심장박출지수\|일주기 리듬\|빛 노출\|휴대폰 사용량\|휴대폰 사용\|스크린 타임\|수면\|깨어난 시간\|삶의 규칙성\|음성\|목소리\|대화\|말하기\|움직임\|활동\|평균 활동량\|평균 소모 에너지\|스포츠\|운동\|걷기\|걸음\|앉기\|뇌파\|뇌전도\|심전도 Ti. |
| 우울.Ti | 웨어러블\|기기\|애플리케이션\|어플리케이션\|어플\|앱\|스마트폰\|스마트 워치\|스마트 밴드\|모바일\|평가\|측정\|디지털 Ti. | 바이오마커\|혈압\|심박변이도\|심박수 변동성\|심박 변동성\|심박수\|심장박동\|심장박출지수\|일주기 리듬\|빛 노출\|휴대폰 사용량\|휴대폰 사용\|스크린 타임\|수면\|깨어난 시간\|삶의 규칙성\|음성\|목소리\|대화\|말하기\|움직임\|활동\|평균 활동량\|평균 소모 에너지\|스포츠\|운동\|걷기\|걸음\|앉기\|뇌파\|뇌전도\|심전도 Ab. |
| 우울.Ti | 웨어러블\|기기\|애플리케이션\|어플리케이션\|어플\|앱\|스마트폰\|스마트 워치\|스마트 밴드\|모바일\|평가\|측정\|디지털 Ab. | 바이오마커\|혈압\|심박변이도\|심박수 변동성\|심박 변동성\|심박수\|심장박동\|심장박출지수\|일주기 리듬\|빛 노출\|휴대폰 사용량\|휴대폰 사용\|스크린 타임\|수면\|깨어난 시간\|삶의 규칙성\|음성\|목소리\|대화\|말하기\|움직임\|활동\|평균 활동량\|평균 소모 에너지\|스포츠\|운동\|걷기\|걸음\|앉기\|뇌파\|뇌전도\|심전도 Ti. |
| 우울.Ti | 웨어러블\|기기\|애플리케이션\|어플리케이션\|어플\|앱\|스마트폰\|스마트 워치\|스마트 밴드\|모바일\|평가\|측정\|디지털 Ab. | 바이오마커\|혈압\|심박변이도\|심박수 변동성\|심박 변동성\|심박수\|심장박동\|심장박출지수\|일주기 리듬\|빛 노출\|휴대폰 사용량\|휴대폰 사용\|스크린 타임\|수면\|깨어난 시간\|삶의 규칙성\|음성\|목소리\|대화\|말하기\|움직임\|활동\|평균 활동량\|평균 소모 에너지\|스포츠\|운동\|걷기\|걸음\|앉기\|뇌파\|뇌전도\|심전도 Ab. |
| 우울.Ab | 웨어러블\|기기\|애플리케이션\|어플리케이션\|어플\|앱\|스마트폰\|스마트 워치\|스마트 밴드\|모바일\|평가\|측정\|디지털 Ti. | 바이오마커\|혈압\|심박변이도\|심박수 변동성\|심박 변동성\|심박수\|심장박동\|심장박출지수\|일주기 리듬\|빛 노출\|휴대폰 사용량\|휴대폰 사용\|스크린 타임\|수면\|깨어난 시간\|삶의 규칙성\|음성\|목소리\|대화\|말하기\|움직임\|활동\|평균 활동량\|평균 소모 에너지\|스포츠\|운동\|걷기\|걸음\|앉기\|뇌파\|뇌전도\|심전도 Ti. |
| 우울.Ab | 웨어러블\|기기\|애플리케이션\|어플리케이션\|어플\|앱\|스마트폰\|스마트 워치\|스마트 밴드\|모바일\|평가\|측정\|디지털 Ti. | 바이오마커\|혈압\|심박변이도\|심박수 변동성\|심박 변동성\|심박수\|심장박동\|심장박출지수\|일주기 리듬\|빛 노출\|휴대폰 사용량\|휴대폰 사용\|스크린 타임\|수면\|깨어난 시간\|삶의 규칙성\|음성\|목소리\|대화\|말하기\|움직임\|활동\|평균 활동량\|평균 소모 에너지\|스포츠\|운동\|걷기\|걸음\|앉기\|뇌파\|뇌전도\|심전도 Ab. |
| 우울.Ab | 웨어러블\|기기\|애플리케이션\|어플리케이션\|어플\|앱\|스마트폰\|스마트 워치\|스마트 밴드\|모바일\|평가\|측정\|디지털 Ab. | 바이오마커\|혈압\|심박변이도\|심박수 변동성\|심박 변동성\|심박수\|심장박동\|심장박출지수\|일주기 리듬\|빛 노출\|휴대폰 사용량\|휴대폰 사용\|스크린 타임\|수면\|깨어난 시간\|삶의 규칙성\|음성\|목소리\|대화\|말하기\|움직임\|활동\|평균 활동량\|평균 소모 에너지\|스포츠\|운동\|걷기\|걸음\|앉기\|뇌파\|뇌전도\|심전도 Ti. |
| 우울.Ab | 웨어러블\|기기\|애플리케이션\|어플리케이션\|어플\|앱\|스마트폰\|스마트 워치\|스마트 밴드\|모바일\|평가\|측정\|디지털 Ab. | 바이오마커\|혈압\|심박변이도\|심박수 변동성\|심박 변동성\|심박수\|심장박동\|심장박출지수\|일주기 리듬\|빛 노출\|휴대폰 사용량\|휴대폰 사용\|스크린 타임\|수면\|깨어난 시간\|삶의 규칙성\|음성\|목소리\|대화\|말하기\|움직임\|활동\|평균 활동량\|평균 소모 에너지\|스포츠\|운동\|걷기\|걸음\|앉기\|뇌파\|뇌전도\|심전도 Ab. |

**Table S7. Search strategy for KISS**

| **Date searched:** December 28, 2025 |
| --- |
| **Total records retrieved:** 906 |
| **Search Query (abstract)** |
| (우울) AND ((웨어러블) OR (애플리케이션) OR (어플) OR (앱) OR (스마트폰) OR (스마트 워치) OR (스마트 밴드) OR (모바일) OR (평가) OR (측정) OR (디지털)) AND ((바이오마커) OR (혈압) OR (심박변이도) OR (심박수 변동성) OR (심박 변동성) OR (심박수) OR (심장박동) OR (일주기 리듬) OR (빛 노출) OR (휴대폰 사용) OR (스크린 타임) OR (수면) OR (음성) OR (목소리) OR (대화) OR (말하기) OR (움직임) OR (활동) OR (활동량) OR (스포츠) OR (운동) OR (걷기) OR (걸음) OR (뇌파) OR (뇌전도) OR (심전도)) |

**Table S8. Search strategy for Cochrane library via Wiley**

| **Date searched:** December 28, 2025 | |
| --- | --- |
| **Total records retrieved:** 2,068 | |
| **Search Query (title, abstract, and keyword)** | |
| #1 | Depressi* |
| #2 | MDD |
| #3 | #1 or #2 |
| #4 | Phenotyp* |
| #5 | Pheno-typ* |
| #6 | Wearable |
| #7 | mobile application |
| #8 | App |
| #9 | Apps |
| #10 | Smart Phone* |
| #11 | Smartphone* |
| #12 | Smart Watch* |
| #13 | Android |
| #14 | iPhone |
| #15 | Mobile phone |
| #16 | Mobile-phone |
| #17 | Quantitative measure* |
| #18 | Quantitative assessment* |
| #19 | Objective measure* |
| #20 | Objective assessment* |
| #21 | Physiological marker* |
| #22 | Sensor* |
| #23 | Sensing |
| #24 | Digital |
| #25 | Passive sens* |
| #26 | GPS |
| #27 | global positioning systems |
| #28 | Accelerometer* |
| #29 | Handheld computer* |
| #30 | Wi-Fi |
| #31 | Actigraph* |
| #32 | Technolog* |
| #33 | Wristband* |
| #34 | Wrist band* |
| #35 | Wrist-worn |
| #36 | Wrist worn |
| #37 | Accelerome* |
| #38 | fitbit |
| #39 | Track* |
| #40 | Pedometer* |
| #41 | #4 or #5 or #6 or #7 or #8 or #9 or #10 or #11 or #12 or #13 or #14 or #15 or #16 or #17 or #18 or #19 or #20 or #21 or #22 or #23 or #24 or #25 or #26 or #27 or #28 or #29 or #30 or #31 or #32 or #33 or #34 or #35 or #36 or #37 or #38 or #39 or #40 |
| #42 | Biomarker* |
| #43 | Blood Pressure |
| #44 | BP |
| #45 | heart rate* |
| #46 | HRV |
| #47 | RHR |
| #48 | light* |
| #49 | light exposure |
| #50 | Phone Usage |
| #51 | Screen Time |
| #52 | Sleep* |
| #53 | REM |
| #54 | Rapid eye movement |
| #55 | regularity of life |
| #56 | circadian rhythm* |
| #57 | acrophase |
| #58 | Awake |
| #59 | Speech |
| #60 | Pitch variability |
| #61 | Voice |
| #62 | Audio |
| #63 | Temperature |
| #64 | Movement* |
| #65 | Motion |
| #66 | Ambulat* |
| #67 | activity |
| #68 | activities |
| #69 | Sport* |
| #70 | Behavioral parameter* |
| #71 | Daily life behavior |
| #72 | Daily-life behavior |
| #73 | Exercise* |
| #74 | Walking |
| #75 | Stepping |
| #76 | Sitting |
| #77 | Sedentary |
| #78 | Supine Position |
| #79 | EEG |
| #80 | Electroencephalogram |
| #81 | EKG |
| #82 | Elektrokardiogramm |
| #83 | ECG |
| #84 | electrocardiogram |
| #85 | electrodermal |
| #86 | Geographic location* |
| #87 | #42 or #43 or #44 or #45 or #46 or #47 or #48 or #49 or #50 or #51 or #52 or #53 or #54 or #55 or #56 or #57 or #58 or #59 or #60 or #61 or #62 or #63 or #64 or #65 or #66 or #67 or #68 or #69 or #70 or #71 or #72 or #73 or #74 or #75 or #76 or #77 or #78 or #79 or #80 or #81 or #82 or #83 or #84 or #85 or #86 |
| #88 | #3 and #41 and #87 |

**Table S9. Search strategy for CINAHL via EBSCOhost**

| **Date searched:** December 28, 2025 | | |
| --- | --- | --- |
| **Total records retrieved:** 3,751 | | |
| **Search Query** | | |
| S1 | Patients | TI ( (Depressi*) or (MDD) ) OR AB ( (Depressi*) or (MDD) ) |
| S2 | Intervention (Methods) | TI ((Phenotyp*) or (Pheno-typ*) or (Wearable) or (mobile application) or (App) or (Apps) or (Smart Phone*) or (Smartphone*) or (Smart Watch*) or (Android) or (iPhone) or (Mobile phone) or (Mobile-phone) or (Quantitative measure*) or (Quantitative assessment*) or (Objective measure*) or (Objective assessment*) or (Physiological marker*) or (Sensor*) or (Sensing) or (Digital) or (Passive sens*) or (GPS) or (global positioning systems) or (Accelerometer*) or (Handheld computer*) or (Wi-Fi) or (Actigraph*) or (Technolog*) or (Wristband) or (Wrist band*) or (Wrist-worn) or (Wrist worn) or (Accelerome*) or (fitbit) or (Track*) or (Pedometer*)) OR AB((Phenotyp*) or (Pheno-typ*) or (Wearable) or (mobile application) or (App) or (Apps) or (Smart Phone*) or (Smartphone*) or (Smart Watch*) or (Android) or (iPhone) or (Mobile phone) or (Mobile-phone) or (Quantitative measure*) or (Quantitative assessment*) or (Objective measure*) or (Objective assessment*) or (Physiological marker*) or (Sensor*) or (Sensing) or (Digital) or (Passive sens*) or (GPS) or (global positioning systems) or (Accelerometer*) or (Handheld computer*) or (Wi-Fi) or (Actigraph*) or (Technolog*) or (Wristband) or (Wrist band*) or (Wrist-worn) or (Wrist worn) or (Accelerome*) or (fitbit) or (Track*) or (Pedometer*)) |
| S3 | Intervention (biomarkers) | TI ((Biomarker*) or (Blood Pressure) or (BP) or (heart rate*) or (HRV) or (RHR) or (light*) or (light exposure) or (Phone Usage) or (Screen Time) or (Sleep*) or (REM) or (Rapid eye movement) or (regularity of life) or (circadian rhythm*) or (acrophase) or (Awake) or (Speech) or (Pitch variability) or (Voice) or (Audio) or (Temperature) or (Movement*) or (Motion) or (Ambulat*) or (activity) or (activities) or (Sport*) or (Behavioral parameter*) or (Daily life behavior) or (Daily-life behavior) or (Exercise*) or (Walking) or (Stepping) or (Sitting) or (Sedentary) or (Supine Position) or (EEG) or (Electroencephalogram) or (EKG) or (Elektrokardiogramm) or (ECG) or (electrocardiogram) or (electrodermal) or (Geographic location*)) OR AB((Biomarker*) or (Blood Pressure) or (BP) or (heart rate*) or (HRV) or (RHR) or (light*) or (light exposure) or (Phone Usage) or (Screen Time) or (Sleep*) or (REM) or (Rapid eye movement) or (regularity of life) or (circadian rhythm*) or (acrophase) or (Awake) or (Speech) or (Pitch variability) or (Voice) or (Audio) or (Temperature) or (Movement*) or (Motion) or (Ambulat*) or (activity) or (activities) or (Sport*) or (Behavioral parameter*) or (Daily life behavior) or (Daily-life behavior) or (Exercise*) or (Walking) or (Stepping) or (Sitting) or (Sedentary) or (Supine Position) or (EEG) or (Electroencephalogram) or (EKG) or (Elektrokardiogramm) or (ECG) or (electrocardiogram) or (electrodermal) or (Geographic location*)) |
| S4 |  | S1 AND S2 AND S3 |

**Table S10. Search strategy for Web of Science via Clarivate**

| **Date searched:** December 28, 2025 | | |
| --- | --- | --- |
| **Total records retrieved:** 7,770 | | |
| **Search Query** | | |
| 1 | Patients | TS=((Depressi*) or (MDD)) |
| 2 | Intervention (Methods) 1 | TS=((Phenotyp*) or (Pheno-typ*) or (Wearable) or (mobile application) or (App) or (Apps) or (Smart Phone*) or (Smartphone*) or (Smart Watch*) or (Android) or (iPhone) or (Mobile phone) or (Mobile-phone) or (Quantitative measure*) or (Quantitative assessment*) or (Objective measure*) or (Objective assessment*) or (Physiological marker*) or (Sensor*)) |
| 3 | Intervention (Methods) 2 | TS=((Sensing) or (Digital) or (Passive sens*) or (GPS) or (global positioning systems) or (Accelerometer*) or (Handheld computer*) or (Wi-Fi) or (Actigraph*) or (Technolog*) or (Wristband) or (Wrist band*) or (Wrist-worn) or (Wrist worn) or (Accelerome*) or (fitbit) or (Track*) or (Pedometer*)) |
| 4 | Intervention (biomarkers) 1 | TS=((Biomarker*) or (Blood Pressure) or (BP) or (heart rate*) or (HRV) or (RHR) or (light*) or (light exposure) or (Phone Usage) or (Screen Time) or (Sleep*) or (REM) or (Rapid eye movement) or (regularity of life) or (circadian rhythm*) or (acrophase) or (Awake) or (Speech) or (Pitch variability) or (Voice) or (Audio) or (Temperature) or (Movement*)) |
| 5 | Intervention (biomarkers) 2 | TS=((Motion) or (Ambulat*) or (activity) or (activities) or (Sport*) or (Behavioral parameter*) or (Daily life behavior) or (Daily-life behavior) or (Exercise*) or (Walking) or (Stepping) or (Sitting) or (Sedentary) or (Supine Position) or (EEG) or (Electroencephalogram) or (EKG) or (Elektrokardiogramm) or (ECG) or (electrocardiogram) or (electrodermal) or (Geographic location*)) |
| 6 | IM 1 OR IM 2 | #2 OR #3 |
| 7 | Ib 1 OR Ib 2 | #4 OR #5 |
| 8 | P AND IM AND Ib | #1 AND #6 AND #7 |

**Table S11. Search strategy for PsycINFO via Ovid**

| **Date searched:** December 28, 2025 | | |
| --- | --- | --- |
| **Total records retrieved:** 12,361 | | |
| **Search Query** | | |
| S1 | Patients | abstract((Depressi*) or (MDD)) OR title((Depressi*) or (MDD)) |
| S2 | Intervention (Methods) | abstract((Phenotyp*) or (Pheno-typ*) or (Wearable) or (mobile application) or (App) or (Apps) or (Smart Phone*) or (Smartphone*) or (Smart Watch*) or (Android) or (iPhone) or (Mobile phone) or (Mobile-phone) or (Quantitative measure*) or (Quantitative assessment*) or (Objective measure*) or (Objective assessment*) or (Physiological marker*) or (Sensor*) or (Sensing) or (Digital) or (Passive sens*) or (GPS) or (global positioning systems) or (Accelerometer*) or (Handheld computer*) or (Wi-Fi) or (Actigraph*) or (Technolog*) or (Wristband) or (Wrist band*) or (Wrist-worn) or (Wrist worn) or (Accelerome*) or (fitbit) or (Track*) or (Pedometer*)) OR title((Phenotyp*) or (Pheno-typ*) or (Wearable) or (mobile application) or (App) or (Apps) or (Smart Phone*) or (Smartphone*) or (Smart Watch*) or (Android) or (iPhone) or (Mobile phone) or (Mobile-phone) or (Quantitative measure*) or (Quantitative assessment*) or (Objective measure*) or (Objective assessment*) or (Physiological marker*) or (Sensor*) or (Sensing) or (Digital) or (Passive sens*) or (GPS) or (global positioning systems) or (Accelerometer*) or (Handheld computer*) or (Wi-Fi) or (Actigraph*) or (Technolog*) or (Wristband) or (Wrist band*) or (Wrist-worn) or (Wrist worn) or (Accelerome*) or (fitbit) or (Track*) or (Pedometer*)) |
| S3 | Intervention (Biomarkers) | abstract((Biomarker*) or (Blood Pressure) or (BP) or (heart rate*) or (HRV) or (RHR) or (light*) or (light exposure) or (Phone Usage) or (Screen Time) or (Sleep*) or (REM) or (Rapid eye movement) or (regularity of life) or (circadian rhythm*) or (acrophase) or (Awake) or (Speech) or (Pitch variability) or (Voice) or (Audio) or (Temperature) or (Movement*) or (Motion) or (Ambulat*) or (activity) or (activities) or (Sport*) or (Behavioral parameter*) or (Daily life behavior) or (Daily-life behavior) or (Exercise*) or (Walking) or (Stepping) or (Sitting) or (Sedentary) or (Supine Position) or (EEG) or (Electroencephalogram) or (EKG) or (Elektrokardiogramm) or (ECG) or (electrocardiogram) or (electrodermal) or (Geographic location*)) OR title((Biomarker*) or (Blood Pressure) or (BP) or (heart rate*) or (HRV) or (RHR) or (light*) or (light exposure) or (Phone Usage) or (Screen Time) or (Sleep*) or (REM) or (Rapid eye movement) or (regularity of life) or (circadian rhythm*) or (acrophase) or (Awake) or (Speech) or (Pitch variability) or (Voice) or (Audio) or (Temperature) or (Movement*) or (Motion) or (Ambulat*) or (activity) or (activities) or (Sport*) or (Behavioral parameter*) or (Daily life behavior) or (Daily-life behavior) or (Exercise*) or (Walking) or (Stepping) or (Sitting) or (Sedentary) or (Supine Position) or (EEG) or (Electroencephalogram) or (EKG) or (Elektrokardiogramm) or (ECG) or (electrocardiogram) or (electrodermal) or (Geographic location*)) |
| S4 |  | [S1] AND [S2] AND [S3] |
