# Supplementary material for: The Role of Digital Biomarkers in Physiological Signal-Based Depression Assessment: Systematic Review and Meta-Analysis
Source: J Med Internet Res. 2026 Apr 2;28:e76432. doi: 10.2196/76432 (PMC13046098; doi:10.2196/76432)
Supplement: Multimedia Appendix 2 [file jmir-v28-e76432-s002.docx]

Multimedia Appendix 2: Detailed study characteristics and quality assessment of the included studies.

Table S1. Summary characteristics of included studies with a single parameter (n=87).

|  | First Author | Year | Country | Design | Participants | | | Digital Biomarker Measure | | Depression Measure | Depression Assessment |
| --- | --- | --- | --- | --- | --- | --- | --- | --- | --- | --- | --- |
|  |  |  |  |  | N | Mean Age (SD or %) | % Male | Device | Duration |  |  |
| **1. Sleep parameter (n=23)** | | | | | | | | | | | |
| 1 | Poon [1] | 2024 | Hong Kong | Prospective | 150 (DG: 75, CG: 75) | DG: 30.41 (11.16)  CG: 30.19 (11.08) | 18.7 | Actiwatch | 1 week | PHQ-9, SCID-I, HADS | Difference |
| 2 | Peng [2] | 2023 | China | - | 73 (DG: 33, CG: 40) | Nr | Nr | Dsi-24, dsi-streamer | Once | HDRS-17, BDI-II | Diag. accuracy |
| 3 | Comas [3] | 2023 | Australia | - | DG: 115 | 47.1 (14.8) | 30.0 | Embla  Compumedics | 2 days | PHQ-9 | Association |
| 4 | Bowman [4] | 2021 | USA | Longitudinal | Healthy female: 302 | Baseline: 46.2 (2.7) | 0 | Actiwatch-64, PSG | 38 days | CES-D | Association |
| 5 | Smith [5] | 2021 | USA | Prospective | 66  (Lung transplant: 42, heart transplant: 24) | Lung transplant: 53.2 (14.0)  Heart transplant: 52.2 (11.0) | 59.0 | Actigraph GT9X | 1 week | CES-D | Association |
| 6 | Mohammadi [6] | 2021 | Iran | - | 60 | 30.0 (12.0) | 53.3 | Miistar-201 system | 5 min | BDI-II | Association |
| 7 | Zhang [7] | 2021 | UK | Prospective | DG: 368 | UK: 46.0, Netherlands: 55.0  Spain: 42.0 | 61.1 | Fitbit charge 2, 3 | 2 years | PHQ-8 | Association |
| 8 | Chen [8] | 2020 | China | - | DG & CG: 35 | DG: 44 (14.54)  CG: 47 (15.88) | 31.4 | Rm6280c | 5 min | HDRS | Association |
| 9 | Hasanzadeh [9] | 2020 | Iran | Cross-sectional | 49 (DG: 26, CG: 23) | DG: 34.7 (13.1)  CG: 37.6 (13.6) | 40.8 | 19-channel Mitsar-EEG 201 machine | 5 min | HDRS, BDI-II | Diag. accuracy |
| 10 | Rajpurkar [10] | 2020 | USA | Prognostic | DG: 518 | 39.0 (12.6) | 47.1 | EEG | 2 min | HDRS-21 | Association |
| 11 | Ding [11] | 2019 | China | Prospective | 348 (DG: 144, CG: 204) | DG: 27.65 (9.50)  CG: 27.46 (9.61) | 36.2 | Eye Tracker, EEG, Grove-GSR monitor | Once | SDS | Diag. accuracy |
| 12 | Gould [12] | 2018 | USA | - | GP: 167 | 71.29 (7.82) | 46.1 | Ambulatory PSG system | Once | GDS | Association |
| 13 | Haghighi [13] | 2017 | Iran | - | DG: 20 | Male: 28.67 (11.93)  Female: 39.53 (9.36) | 15.0 | Mistar 21-channel amplifier | 20 min | HDRS | Association |
| 14 | Lee [14] | 2014 | Korea | - | DG & CG: 282 | Mild/Moderate DG: 68.99 (5.62)  Severe DG: 67.28 (6.01)  CG: 67.68 (4.91) | 40.4 | Psg | Once | GDS | Association |
| 15 | Maglione [15] | 2014 | USA | Longitudinal | DG: 952 | 82.5 | 0 | Sleepwatch-O | 4.1 nights | GDS | Association |
| 16 | Pillai [16] | 2014 | USA | - | Uni. students with depressive symptoms: 39 | 19.6 (3.2) | 26.2 | Actiwatch 2 | 7 days | BDI-II | Corr. in DG |
| 17 | Maglione [17] | 2012 | USA | Cross-sectional | GP: 3045 | 83.6 (3.8) | 0 | Sleepwatch-O | 72 days | GDS | Association |
| 18 | Bei [18] | 2010 | Australia | 2-stage longitudinal | Low-risk postpartum depression: 44 | 30.41(5.59) | 0 | Wrist actigraph | 1 week | DASS, HADS | Corr. in GP |
| 19 | Goyal 19] | 2009 | USA | Longitudinal RCT | Pregnancy third-trimester group: 112 | 32.5(4.6) | 0 | Wrist actigraph | 48 h | CES-D | Association |
| 20 | Coffield [20] | 2004 | USA | 2×2 mixed design | 56 (DG: 33, CG: 23) | Nr | Nr | Motionlogger  Actigraphs™ | 1 week | BDI, IDD | Corr. in DG |
| 21 | Nofzinger [21] | 1999 | USA | - | 14 (DG: 6, CG: 8) | DG: 35.0, CG: 37.0 | 21.4 | PET, MRI | Once | BDI, HDRS | Difference |
| 22 | Luthringer [22] | 1996 | France | RCT | 24 (Medication group: 12, placebo group: 12) | Medication (venlafaxine) group: 45.3 (10.1),  Placebo group: 43.8 (15.6) | 41.7 | EEG derivations | Twice  (1 week, 1 month) | HDRS, MADRS | Difference |
| 23 | Roemer [23] | 1992 | USA | Cross-sectional | DG: 31 | 63.8 (14.6) | 22.6 | Cadwell Spectrum 32tm | 10 min | HDRS | Corr. in DG |
| **2. Cardiac parameter (n=19)** | | | | | | | | | | | |
| 24 | Theofilis [24] | 2023 | Greece | Cross-sectional | DG & CG: 1637 | 40–99 | 40.1 | ECG | Once | SDS, GDS | Association |
| 25 | Siddi [25] | 2023 | Spain | Cohort | Recurrence DG: 510 | 46.6 (15.1) | 24.3 | Fitbit | 1 week | PHQ | Association |
| 26 | Subramaniam [26] | 2023 | India | Prospective | DG: 80 | 42.8 | Nr | Biopac mp100 | 3 h | HDRS-17 | Difference |
| 27 | Lee [27] | 2022 | USA | Cross-sectional | DG & CG: 864 | 20–39: (13.0%)  40–59: (27.7%)  60–79: (47.3%)  80+: (12.0%) | 91.7 | Sphygmomanometer | Once | PHQ-9 | Association |
| 28 | Chung [28] | 2021 | USA | One-group pre-post | GP: 14 | 33.0 (6.0) | 36 | Lief smart patch | 8 weeks | PHQ-2 | Corr. in GP |
| 29 | da Estrela [29] | 2021 | Canada | - | 222 (Mothers with chronic caregiving stress: 125, CG: 97) | Mothers with chronic caregiving stress: 47.71 (6.15)  CG: 45.69 (5.69) | Nr | Polar RS800CX | 5 min | CES-D | Corr. in GP |
| 30 | Lee [30] | 2021 | Korea | - | DG: 34 | 43.76 (9.3) | 5.9 | SA-2000E analyzer | 5 min | HDRS, MADRS | Corr. in DG |
| 31 | Coutts [31] | 2020 | UK | - | DG & CG  Exp. 1: 91, Exp. 2: 584 | Exp. 1: 21, Exp. 2: 22 | Exp. 1: 38.0  Exp. 2: 28.0 | Biobeam band | 2−5 min | DASS | Diag. accuracy |
| 32 | Huang [32] | 2018 | USA | Cross-lagged twin study | DG & CG: 146 | Baseline: 54.0 (3.0)  Follow-up: 61.0 (3.0) | 100 | Ambulatory ECG | 24 h | BDI-II | Association |
| 33 | Hamilton [33] | 2017 | USA | Longitudinal | DG: 102 | 19.86 (1.17) | 21.0 | Bioharness MP150 | 2 weeks | BDI-II, 7U7D | Association |
| 34 | Sun [34] | 2016 | Japan | - | 91 (Drug-naïve DG: 44,  CG: 47) | Drug-naïve DG: 43 (12)  CG: 41 (12) | 48.4 | ECG | Once | SDS | Difference |
| 35 | Liang [35] | 2015 | Taiwan | Cross-sectional | 205 (DG: 156, CG: 49) | DG: 24.4 (3.0)  CG: 24.7 (1.5) | Nr | EEG | 5 min | BDI-II | Difference |
| 36 | Roh [36] | 2014 | Korea | - | DG: 23 | Nr | Nr | Cardiac monitoring patch | Nr | BDI | Diag. accuracy |
| 37 | Lee [37] | 2012 | Korea | - | GP: 19 | 30.4 | 52.6 | Biopac MP150, PPG | 5 min | BDI | Corr. in GP |
| 38 | Zhang [38] | 2012 | Korea | - | 24 (DG: 10, CG: 14) | Nr | 2.5 | Holter monitor | 13 min | SDS | Diag. accuracy |
| 39 | Licht [39] | 2008 | Netherlands | Cross-sectional | 2981 (Recovered DG: 2329, CG: 652) | 41.8 | 33.2 | VU-AMS | Once | IDS-SR | Difference |
| 40 | Kojima [40] | 2008 | Japan | - | Renal failure: 119 | 55.2 (10.5) | 55.46 | Scm2000 | 24 h | BDI-II | Corr. in GP |
| 41 | Stroup-Benham [41] | 2000 | USA | Population-based study | GP: 2723 | 72.62 (0.19) | 42.1 | Mercury sphygmomanometer | Once | CES-D | Association |
| 42 | Stein [42] | 2000 | USA | - | 70 (DG: 40, CG: 30) | Mild DG: 60.2 (8.1), Moderate/Severe DG: 60.5 (9.1), CG: 62.4 (8.9) | 60.0 | Marquette Series 8500 Holter monitors | 24 h | BDI | Difference |
| **3. Physical activity (n=16)** | | | | | | | | | | | |
| 43 | Wang [43] | 2023 | USA | - | DG: 722 | Mild DG: 52 (8.7)  Moderate DG: 55 (7.7)  Severer DG: 54 (7.9) | 40.0 | Apple watch | 30 days | CES-D | Association |
| 44 | Espino-Salinas [44] | 2022 | Mexico | - | DG & CG: 55 | Nr | Nr | Actiwatch | 1 week | MADRS | Diag. accuracy |
| 45 | Hsiao [45] | 2022 | Taiwan | Cross-sectional | GP: 167 | 80.2 (7.0) | 50.9 | Actigraph wgt3x-BT  Triaxial accelerometer | 1 week | CES-D, GDS-15, GDS-30, PHQ-9 | Association |
| 46 | Ramsey [46] | 2022 | USA | Observational | DG: 66 | 18–40: (47.0%)  41–70: (53.0%) | 71.2 | Actigraph | 24 weeks | PHQ-9 | Association |
| 47 | Hussenoeder [47] | 2022 | Germany | Cohort | GP: 1,451 | 55.0 (11.8) | 47.9 | Sensewear, Actigraphy | 1 week | CES-D | Association |
| 48 | Michalak [48] | 2022 | Germany | - | 71 (DG: 35, CG: 36) | DG: 39.57 (12.3)  CG: 39.06 (10.0) | 39.4 | Accelerometers | 2 days | BDI-II | Association |
| 49 | Li [49] | 2022 | Germany |  | GP: 78 | 25.46 (6.18) | 73.1 | Movisens ECG move 3  Chest belt | 2 weeks | PHQ-9 | Association |
| 50 | Figueroa [50] | 2021 | USA | Observational | DG: 274 | Mild DG: 56.0  Severer DG: 50.5 | 0 | Omron active style pro hja-350it | 17.6 days | CES-D | Association |
| 51 | Hsueh [51] | 2021 | Taiwan | Prospective | GP: 274 | 74.5 (6.1) | 45.6 | Actigraph GT3X+ | 5 days | GDS-15 | Diag. accuracy |
| 52 | Jakobsen [52] | 2020 | Norway | Observational cohort | 55 (DG: 23, CG: 32) | DG: 42.8 (11.0)  CG: 38.2 (13.0) | 45.5 | Wrist-worn actigraph | 14 days | MADRS | Diag. accuracy |
| 53 | Zhao [53] | 2019 | China | - | GP: 179 | 24.2 (1.5) | 55.9 | Microsoft Kinect camera | Once | PHQ-9 | Diag. accuracy |
| 54 | Averill [54] | 2018 | New Zealand | Cohort | DG: 24 | 38.08 (13.625) | 62.5 | Actiwatch 2  Fitbit | 3 weeks | QIDS-C16 | Corr. in DG |
| 55 | Ku [55] | 2018 | Taiwan | Prospective  Cohort | GP: 274 | 74.5 (6.1) | 45.6 | Actigraph GT3X+ | 7 days | GDS-15 | Diag. accuracy |
| 56 | Holmquist [56] | 2017 | Sweden | - | GP: 2084 | 70.0 (0.0) | 51.0 | Gaitrite  Actigraph | 1 week | GDS-15 | Corr. in GP |
| 57 | Todder [57] | 2009 | Israel | Prospective | 54 (DG: 27, CG: 27) | Male: 48.6 (12.9)  Female: 50.5 (13.2) | 53.7 | Actiwatch S | 14 days | DSM-IV, HDRS-21, BDI | Association |
| 58 | Lemke [58] | 2000 | Germany | Quantitative analysis | 32 (DG: 16, CG: 16) | DG: 44.1 (12.8)  CG: 43.6 (11.0) | 50.0 | Walkway | Once | HDRS, SHAPS-D, SDS, WDRS | Corr. in DG |
| **4. Smartphone parameter (n=9)** | | | | | | | | | | | |
| 59 | Fadul [59] | 2023 | UAE | - | 24 (DG: 10, CG: 14) | DG: 23.6 (3.24)  CG: 23.86 (4.44) | 37.5 | Mobile app | 3 months | PHQ-9 | Diag. accuracy |
| 60 | Auerbach [60] | 2022 | USA | - | 46 (DG: 22, CG: 24) | DG: 22.2 (3.45)  CG: 21.8 (3.37) | 26.09 | Smartphone GPS  Metricwire | 14 days | IDAS | Diag. accuracy |
| 61 | Otte Andersen [61] | 2022 | Denmark | - | GP: 6407 | GP: 36.6 (10.2), Citizen science group: 44.4 (14.5) | 35.30 | Smartsleep app | 2 weeks | MDI | Diag. accuracy |
| 62 | Opoku Asare [62] | 2021 | Finland | Longitudinal observational | DG & CG: 629 | 18–24: 73 (11.6%)  25–34: 204 (32.4%)  35–44: 156 (24.8%)  45–64: 166 (26.4%)  65+: 30 (4.8%) | 86.8 | Smartphone | For a mean of 22.1 days | PHQ-8 | Diag. accuracy |
| 63 | Chikersal [63] | 2021 | USA | - | Uni. students: 138 | Nr | Nr | Smartphones and wearable fitness trackers | 16 weeks | BDI-II | Diag. accuracy |
| 64 | Zhang [64] | 2021 | UK | Longitudinal | DG: 316 | 51.0 (35.0) | 25.9 | Prmt, armt app  active tracker | 2 years  follow-up | PHQ-9 | Diag. accuracy |
| 65 | Pedrelli [65] | 2020 | USA | Prospective | DG: 31 | 33.7 (14.0) | 26 | E4 Empatica wristbands | 8 weeks | HDRS-17 | Diag. accuracy |
| 66 | Mastoras [66] | 2019 | Greece | Cohort | 25 (DG: 11, CG: 14) | DG: 23.55 (3.24)  CG: 23.86 (4.44) | 60.00 | Typeofmood app | 124 days | PHQ-9 | Diag. accuracy |
| 67 | Saeb [67] | 2015 | USA | Observational | GP: 28 | 28.9(10.1) | 28.57 | Mobile phone | 2 weeks | PHQ-9 | Diag. accuracy |
| **5. Speech parameter (n=10)** | | | | | | | | | | | |
| 68 | Wiseman [68] | 2025 | Canada | Cross-sectional | DG: 66  CG: 54 | DG: 45.2  (15.1)  CG: 44.5  (17.2) | DG: 45.5  CG: 24.1 | Mobile app,  Eyelink 100 Plus | 1 time | HAMD-17 | Diag.  accuracy |
| 69 | Wadle [69] | 2024 | Germany | Prospective | DG: 22 | 33.5(12.4) | Nr | Galaxy J7 | 3 weeks | ADS-K, MADRS | Association |
| 70 | Silva [70] | 2024 | Brazil | Observational case-control | 144 (DG: 54, CG: 90) | Age range: 18–32/32–46/46–60  DG: 20.4%, 40.7%, 38.9%  CG: 48.9%, 28.9%, 22.2% | 34.72 | Adapted vocal screening protocol | 45 min | BDI-II | Association |
| 71 | Kim [71] | 2023 | Korea | Prospective observational | 318 (DG: 153, CG: 165) | >19 | DG: 33.33  CG: 25.45 | Smartphone | Once | HDRS, PHQ-9 | Diag. accuracy |
| 72 | Wang [72] | 2023 | China | Longitudinal | Uni. Students with depressive symptoms: 47 | 20.51 (1.50) | 10.64 | Mobile phone | 20 min, 3 times a week | PHQ-9, HDRS-17 | Corr. in DG |
| 73 | Zhao [73] | 2022 | China | - | 133 (DG: 71, CG: 62) | DG: 34.90 (9.32)  CG: 36.67 (8.56) | 32.33 | ISK BM-500 microphone | 3 min | HDRS-17, PHQ-9 | Diag. accuracy |
| 74 | Ye [74] | 2021 | China | - | 160 (DG: 80, CG: 80) | 18–65 | 49 | Kinect 2.0, camera, Sound card | Once | HDRS | Diag. accuracy |
| 75 | Klangpornkun [75] | 2021 | Thailand | Screening test | 66 (DG: 27, Other mental disorders: 12, CG: 27) | DG: 37.63 (16.16)  Other mental disorders: 41.08 (13.37)  CG: 35.96 (13.58) | 28.79 | Mobile  app | 40 min to 1.5 h | PHQ-9, HDRS | Diag. accuracy |
| 76 | Demiroglu [76] | 2020 | Turkey | Observational | DG & CG: 343 (Turkish: 70, German: 84, English: 189) | Turkish: 34  German: 31.5  English: Nr | Turkish: 20.00, German &  English: Nr | Headphone microphone | Once | BDI-II, PHQ-8 | Diag. accuracy |
| 77 | Yamamoto [77] | 2020 | Japan | - | 223 (DG: 84, CG: 71  Bipolar: 68) | DG: 50.0 (15.0)  CG: 64.3 (17.3)  Bipolar: 55.1 (17.0) | 44.84 | Microphone | 10 min | HDRS-17 | Diag. accuracy |
| **6. Circadian rhythm (n=7)** | | | | | | | | | | | |
| 78 | Ali [78] | 2023 | USA | RCT | 40 (DG with improvement: 16, DG without improvement: 24) | DG with improvement: 32.18 (17.44)  DG without improvement: 30.24 (14.20) | 30 | Actical | 1 week | HDRS | Difference |
| 79 | Braund [79] | 2022 | Australia | Prospective longitudinal | 121 (DG: 79, Bipolar: 42) | DG: 41.63 (13.94)  Bipolar: 41.00 (13.16) | 33.9 | Mobile app  GPS | 10 weeks | PHQ-9 | Association |
| 80 | Choi [80] | 2021 | Korea | - | GP: 1552 | 42.1 (22.99) | 44.90 | Actigraph GT3X+ | 1 week | PHQ-9 | Diag. accuracy |
| 81 | George [81] | 2021 | Netherlands | Longitudinal | 46 (DG: 21, CG: 25) | 20–50 | Nr | Actical | 30 days | BDI-II | Difference |
| 82 | Smagula [82] | 2015 | USA | Longitudinal | DG: 2,124 | 76.2 (5.5) | 100 | Actigraph | 4.8 days | GDS | Association |
| 83 | Maglione [83] | 2014 | USA | Prospective cohort | 3020 (DG: 1059, CG: 1961) | 83.55 (3.79) | 0 | Sleepwatch-O | 3 days | GDS | Association |
| 84 | Luik [84] | 2013 | Netherlands | Cohort | GP: 1734 | 62.25 (9.35) | 46.6 | Actiwatch AW4 | 7 days | CES-D | Association |
| **7. EEG (n=2)** | | | | | | | | | | | |
| 85 | Anik [85] | 2024 | Bangladesh | Observational | DG: 34  CG: 30 | DG: 40.3 (12.9)  CG: 38.3 (15.6) | DG: 50  CG: 70 | 19-node EEG cap | 10 min | BDI  HDRS | Diag. accuracy |
| 86 | Tian [86] | 2025 | China | Observational, cross-sectional | DG: 73  CG: 108 | DG:  16–56  CG:  18–55 | Nr | Wearable EEG | < 1 min | PHQ-9 | Diag. accuracy |
| **8. Video parameters (n=1)** | | | | | | | | | | | |
| 87 | Islam [87] | 2024 | USA | Longitudinal observational | GP: 25 | 27.88 | 44 | Mobile App | 4 weeks | PHQ-9 | Diag.  accuracy |
| *SD* Standard Deviation, *DG* Depression Group, *CG* Control Group, *PHQ* Patient Health Questionnaire, *SCID-I* Semi-Structured Clinical Interview, *HADS* Hospital Anxiety and Depression Scale, *Nr* Not Reported, *DSI* Depressive Symptom Inventory, *HDRS* Hamilton Depression Rating Scale, *BDI* Beck Depression Inventory, *Diag.* Diagnostic, *USA* United States Of America, *PSG* Polysomnography, *CES-D* Center for Epidemiologic Studies Depression Scale, *UK* United Kingdom, *EEG* Electroencephalography, *GSR* Galvanic Skin Response, *SDS* Zung Self-Rating Depression Scale, *GP* General Population, *GDS* Geriatric Depression Scale, *Uni.* University, *Corr.* Correlation, *DASS* Depression Anxiety Stress Scale, *RCT* Randomized Controlled Trial, *IDD* Inventory to Diagnose Depression, *PET* Positron Emission Tomography, *MRI* Magnetic Resonance Imaging, *MADRS* Montgomery-Åsberg Depression Rating Scale, *ECG* Electrocardiogram, *7U7D* 7 Up 7 Down Inventory, *PPG* Photoplethysmography, *VU-AMS* Vrije Universiteit Ambulatory Monitoring System, *IDS-SR* 30-Item Inventory of Depressive Symptomatology, *QIDS* Quick Inventory of Depressive Symptomatology, *SHAPS-D* Snaith±Hamilton-Pleasure Scale (German Version), *WDRS* Widloècher Depression Retardation Scale, *UAE* United Arab Emirates, *App* Application, *GPS* Global Positioning System, *IDAS* Inventory of Depression and Anxiety Symptoms, *MDI* Major Depression Inventory, *Prmt* Passive Remote Monitoring Tool, *Armt* Active Remote Monitoring Tool, and *ADS-K* Allgemeine Depressionsskala – Kurzform | | | | | | | | | | | |

Table S2. Summary characteristics of included studies with multiple parameters (n=45).

|  | First Author | Year | Country | Design | Participants | | | Parameter Type | Digital Biomarker Measure | | Depression Measure | Depression Assessment |
| --- | --- | --- | --- | --- | --- | --- | --- | --- | --- | --- | --- | --- |
|  |  |  |  |  | N | Mean Age (SD) | % Male |  | Device | Duration |  |  |
| 1 | Makhmutova [88] | 2022 | Switzerland | - | GP: 4036 | Nr | Nr | Sleep, PhAct | Fitbit | 2 weeks | PHQ-9 | Diag. accuracy |
| 2 | Price [89] | 2024 | USA | - | 8378  DG: 766, CG: 7612 | DG: 48.61 (16.95)  CG: 47.48 (18.69) | DG: 34.85  CG: 50.20 | Sleep, PhAct | Actigraph GT3X+ | 1 week | PHQ-9 | Diag. accuracy |
| 3 | Bae [90] | 2023 | Korea | - | DG & CG: 283 | 75.8 (3.7) | 97(34.28) | Sleep, PhAct | Fitbit | 20 days | SGDS-K | Association |
| 4 | Griffiths [91] | 2022 | UK | Pre and post intervention | Treatment-resistant DG: 24 | 46.83 (14.02) | 5(21) | Sleep, PhAct | Fitbit | 5 weeks | PHQ-9 | Diag. accuracy |
| 5 | Mishra [92] | 2021 | USA | Prospective | Adults: 10 | 77.3 (1.9) | 6(60.00) | Sleep, PhAct | PAMSys™ | 48 h | CES-D | Corr. in GP |
| 6 | Raoux [93] | 1994 | France | Prospective | DG: 26 | 43.9 (21.0) | 6(23.08) | Sleep, PhAct | Nondominant wrist | 3 days | MADRS | Corr. in DG |
| 7 | Rykov [94] | 2021 | Singapore | Cross-sectional | DG & CG: 267 | 32.8 (8.6) | 36.3 | Sleep, Cardiac | Fitbit Charge 2 | 14 days | PHQ-9 | Diag. accuracy |
| 8 | Sayar [95] | 2002 | Turkey | Comparative | 42  DG: 21, CG: 21 | DG: 38.0 (9.1)  CG: 43.7 (8.6) | 23.81 | Sleep, Cardiac | Holter monitors | 24 h | HDRS | Corr. in DG |
| 9 | Landolt [96] | 2005 | Switzerland | Cross-sectional | 32  DG: 16, CG: 16 | DG: 41.2  CG: 41.1 | 50.00 | Sleep, SPhone | Polygraphic amplifier | at least 1 night | BDI, HDRS | Difference |
| 10 | Hoyos [97] | 2020 | Australia | - | 64  DG: 34, CG: 30 | DG: 63.85 (10.76)  CG: 65.70 (9.11) | 53.13 | Sleep, CircRhythm | Actiwatch Polysomnography | 2 weeks | HDRS | Association |
| 11 | Lorenz [98] | 2019 | Germany | Cohort | 242  DG: 121, CG: 121 | DG: 56.52 (9.96)  CG: 57.01 (10.50) | 76.03 | Sleep, CircRhythm | SenseWear  actigraphy | 1 week | CES-D | Difference |
| 12 | Vanderlind [99] | 2014 | USA | Prospective | GP: 35 | 19.83 (1.25) | 60.00 | Sleep, CircRhythm | Actigraph™ | 3 weeks | CES-D | Corr. in GP |
| 13 | Ahmed [100] | 2022 | USA | Observational | DG: 87 | Nr | Nr | PhAct, Cardiac | Customized wristband | 5 days | BDI-II | Diag. accuracy |
| 14 | Choi [101] | 2022 | USA | - | GP: 14 | 76 (oldest 86) | 14.29 | PhAct, Cardiac | Wearable band | 71 days | PHQ-9, SGDS | Diag. accuracy |
| 15 | Mahendran [102] | 2019 | India | - | DG: 450 | 40 | Nr | PhAct, Cardiac | Mi band-3 | 1 week | HDRS | Diag. accuracy |
| 16 | Xu [103] | 2019 | USA | - | DG & CG: 350 | Nr | Nr | PhAct, SPhone | Smartphone  Fitbit tracker | 106~113 days | BDI-II | Diag. accuracy |
| 17 | Lu [104] | 2018 | USA | - | Uni. students: 103 | 18–25 | 23.3 | PhAct, SPhone | Smartphone, Fitbit | 10 weeks | QIDS | Diag. accuracy |
| 18 | Farhan [105] | 2016 | USA | Longitudinal observational | DG & CG: 79 | 18–25 | 26.1 | PhAct, SPhone | Smartphone (iOS, Android) | 8 months | PHQ-9 | Diag. accuracy |
| 19 | Karimi [106] | 2025 | USA | Observational | DG: 60 | DG: 20–70 | DG: 48 | Cardiac,  CircRhythm | Wearable ECG,  SpO2 sensor | 120 min | BDI-II  PHQ-9 | Diag. accuracy |
| 20 | Jacobson [107] | 2020 | USA | Longitudinal observational | DG: 31 | 19.13 | 35.48 | CircRhythm, SPhone | Smartphone | 8 days | DASS | Diag. accuracy |
| 21 | Wang [108] | 2018 | USA | - | Uni. students: 83 | 20.13 (2.31) | 48.2 | CircRhythm, SPhone | Smartphone  Wearable passive mobile | 9 weeks | PHQ-8, PHQ-4 | Diag. accuracy |
| 22 | Di Matteo [109] | 2020 | Canada | Observational | GP: 84 | 30 (8.6) | 58 | SPhone, Speech | Mobile app | 2 weeks | PHQ-8, SDS | Corr. in GP |
| 23 | Zhou [110] | 2022 | China | - | DG: 52 | 60–74: 82.7%  75+: 17.3% | 19.23 | Speech, Video | Digital video | 7 min | PHQ-9 | Diag. accuracy |
| 24 | Williamson [111] | 2019 | USA | Longitudinal | WIBD data: 18 (DG: 12, CG: 6)  Mundt data: 35 (DG 35) | WIBD data: Nr  Mundt data: 41.8 | WIBD data: DG 50, CG 50  Mundt data: DG 42.86 | Speech, Video | Lapel microphone  HD video camera | WIBD: 8 weeks  Mundt: 6 weeks | HDRS | Diag. accuracy |
| 25 | Shah [112] | 2021 | USA | Longitudinal | DG: 14 | 21.6 (2.8) | 4 | Sleep, PhAct, Cardiac | Mobile App  Wristwatch, wireless EEG | 30 days | PHQ-9 | Association |
| 26 | Asare [113] | 2022 | Finland | Longitudinal | DG & CG: 54 | 43.04 (11.58) | 44.4 | Sleep, PhAct, SPhone | Smartphone  Oura Rings | 30 days | DASS-21 | Diag. accuracy |
| 27 | Narziev [114] | 2020 | Korea | - | 21  DG: 16, CG: 5 | Nr | Nr | Sleep, PhAct, SPhone | Smartphone | 4 weeks | PHQ-9, BDI-II | Diag. accuracy |
| 28 | Kim [115] | 2019 | Korea | - | 47  DG: 18, CG: 29 | 78.0 (5.24) | 3 | Sleep, PhAct, SPhone | Actiwatch  Spectrum PRO | 2 weeks | SGDS-K, K-HDRS | Diag. accuracy |
| 29 | Kumagai [116] | 2019 | Japan | - | DG: 89 | 44.3 (10.8) | 55.1 | Sleep, PhAct, SPhone | Mobile app  Wristwatch | 1 year | PHQ-9 | Association |
| 30 | Funkhouser [117] | 2025 | USA | Second analysis of RCT | DG: 47 | 22.04 (2.15) | 25 | Sleep,  PhAct, GPS | Mobile app | 12 weeks | PHQ-8 | Association |
| 31 | Di Matteo 118] | 2021 | Canada | Cross-sectional | GP: 84 | 28.8 (8.6) | 58 | Sleep, SPhone, Speech | Smartphone | 2 weeks | PHQ-8 | Diag. accuracy |
| 32 | Sverdlov [119] | 2021 | Netherlands | Cross-sectional | 40  DG: 20, CG: 20 | DG: 35.0 (14.9)  CG: 27.5 (8.86) | DG:15.0  CG: 20.0 | Sleep, SPhone, Speech | Mobile App, EEG | 2 weeks | HDRS, MADRS, PHQ-9 | Diag. accuracy |
| 33 | Ben-Zeev [120] | 2015 | USA | Cohort | GP: 47 | 22.5 | 79.0 | Sleep, SPhone, Speech | Smartphone sensors | 10 weeks | PHQ-9 | Association |
| 34 | Minaeva [121] | 2020 | Netherlands | Ambulatory assessment | DG & CG: 54 | 20–50 | Nr | Sleep, SPhone, CircRhythm | Wrist-worn accelerometer | 30 days | BDI-II | Diag. accuracy |
| 35 | Dai [122] | 2022 | USA | RCT | DG: 89 | 46.7 (11.7) | 22.5 | PhAct, Cardiac, SPhone | Wearable activity tracker | 6 months | PHQ-9 | Diag. accuracy |
| 36 | Tazawa [123] | 2020 | Japan | - | 86  DG: 45, CG: 41 | DG: 52.1 (13.2)  CG: 69.1 (14.2) | DG: 53.3  CG: 53.7 | PhAct, Cardiac, SPhone | Wrist wearable device | 1 week | HDRS | Diag. accuracy |
| 37 | Thati [124] | 2023 | India | - | DG & CG: 102 | >18 | 44 | SPhone, Speech, Video | Mobile phone | 2 weeks | PHQ-9 | Diag. accuracy |
| 38 | Jiang [125] | 2024 | USA | Cross-sectional | DG: 51  CG: 22 | DG: 36.6 (13.2)  CG: 42.7 (14.0) | DG: 19.6  CG: 40.9 | Speech,  CircRhythm  Video | Remote telehealth video interviews (Zoom) | - | PHQ-9 | Diag. accuracy |
| 39 | Moshe [126] | 2021 | Finland | Longitudinal observational | GP: 55 | 42.8 (11.6) | 45.5 | Sleep, PhAct, Cardiac, SPhone | iPhone  Oura Ring | 30 days | DASS-21 | Association |
| 40 | Wang [127] | 2014 | USA | Longitudinal | Uni. students: 48 | Nr | 79.17 | Sleep, PhAct, SPhone, Speech | Smartphone | 10 weeks | PHQ-9 | Corr. in GP |
| 41 | Song [128] | 2024 | Korea | Observational longitudinal | GP: 25 | GP: 76.40  (4.23) | GP:  24 | PhAct,  Cardiac,  SPhone,  GPS | Wearable device  (Fitbit Sence)  Mobile app,  Camera | 6 weeks | PHQ-9,  GDS-15 | Association |
| 42 | Bai [129] | 2021 | China | Prospective | DG: 261 | Nr | Nr | Sleep, PhAct, Cardiac, SPhone, CircRhythm | Mobile App, Mi Band 2 | - | PHQ-9 | Diag. accuracy |
| 43 | Borelli [130] | 2025 | USA | Longitudinal | GP: 28 | GP: 19.96 (1.23) | GP: 46 | Sleep,  PhAct,  Cardiac,  CircRhythm,  SPhone | Oura ring,  Smartwatch,  Smartphone | 19 weeks | PHQ-9 | Diag. accuracy |
| 44 | Chen [131] | 2024 | China | Case-control | DG: 80  CG: 76 | DG: 49.94 (11.0)  CG: 50.17 (12.0) | DG: 27.5  CG: 36.8 | Sleep, PhAct,  CircRhythm,  Speech, Video | Actiwatch Spectrum Plus,  Mobile app | 1 week | HADS | Diag. accuracy |
| 45 | Aledavood [132] | 2025 | Finland | Observational longitudinal | DG: 121  CG: 30 | DG: 34.7 (12.7)  CG: 45 (14.0) | DG: 28.9  CG: 23 | Sleep, PhAct, Cardiac,  SPhone,  Speech,  GPS | Smartphone,  Mobile app,  Actiwatch 2, Under | Active phase:  2 weeks  Passive phase:  1 years | PHQ-9, MINI | Association |
| *SD* Standard Deviation, *GP* General Population, *Nr* Not Reported, *PhAct* Physical Activity, *PHQ* Patient Health Questionnaire, *Diag.* Diagnostic, *DG* Depression Group, *CG* Control Group, *SGDS* Shortened Version of the Geriatric Depression Scale, *UK* United Kingdom, *USA* United States of America, *PAMSys* Physical Activity Monitoring System, *CES-D* Center for Epidemiologic Studies Depression Scale, *Corr.* Correlation, *MADRS* Montgomery-Åsberg Depression Rating Scale, *HDRS* Hamilton Depression Rating Scale, *SPhone* Smartphone, *BDI* Beck Depression Inventory, *Circrhythm* Circadian Rhythm, *Uni.* University, *QIDS* Quick Inventory of Depressive Symptomatology, *DASS* Depression Anxiety Stress Scale, *App* Application, *SDS* Zung Self-Rating Depression Scale, *HD* High Definition*, EEG* Electroencephalography, and *RCT* Randomized Controlled Trial, *MINI* Mini-International Neuropsychiatric Interview | | | | | | | | | | | | |

Table S3. Detailed features by category of digital biomarkers.

| Feature | | n | Definition |
| --- | --- | --- | --- |
| **1. Sleep parameters** | | | |
| Total sleep time (TST) | | 41 | The total duration of sleep during the night, measured in minutes or hours. |
| Sleep efficiency (SE) | | 21 | The percentage of time spent asleep while in bed, calculated as (TST / TIB) × 100. |
| Wake after sleep onset (WASO) | | 13 | The total time spent awake after initially falling asleep, measured in minutes. |
| Sleep fragmentation | | 6 | The frequency of awakenings or sleep interruptions during the night. |
| Sleep onset latency (SOL) | | 12 | The amount of time it takes to fall asleep after "lights off," measured in minutes. |
| Time in bed (TIB) | | 8 | The total duration spent in bed, regardless of whether asleep or awake, measured in hours or minutes. |
| Rapid eye movement sleep (REM sleep) | | 7 | The sleep stage characterized by REMs, measured in minutes |
| REM sleep latency | | 6 | The time from sleep onset to the first REM period, including any awakenings during this interval. |
| Non-rapid eye movement sleep (NREM sleep) | | 3 | Sleep stages excluding REM sleep, measured in minutes. |
| Slow-wave sleep (SWS) | | 4 | The deepest sleep stage (stages 3 and 4), characterized by slow brain waves, measured in minutes. |
| Sleep onset | | 9 | The clock time at which sleep begins (e.g., hh: mm). |
| Sleep midpoint | | 5 | The calculated midpoint of sleep duration (e.g., hh: mm). |
| Sleep offset | | 8 | The clock time at which sleep ends (e.g., hh: mm). |
| **2. Physical activity** | | | |
| Physical activity counts | | 18 | A measure of physical activity based on triaxial accelerometer data, typically collected at regular intervals (e.g., every 30 s). |
| Step counts | | 17 | The total number of steps taken during a specified time period, typically measured daily. |
| Sedentary time | | 16 | The total duration of inactivity or minimal activity (e.g., sitting or lying down) during a given period, often measured using metabolic equivalents (METs) or low step counts. |
| Moderate-to-vigorous physical activity (MVPA) | | 10 | Physical activity of moderate to high intensity. |
| Light physical activity (LPA) | | 8 | Physical activity of low intensity |
| Energy expenditure | | 3 | The amount of energy burned during physical activity, typically measured in kilocalories (kcal) or METs. |
| **3. Cardiac parameters** | | | |
| **Time domain** | Mean heart rate (mHR) | 22 | The average number of heart beats per minute over a specific time period. |
|  | Standard deviation of the normal-to-normal intervals (SDNN) | 13 | The standard deviation of the intervals between successive heartbeats (NN intervals). |
|  | Root mean square of the successive differences (RMSSD) | 11 | The square root of the mean of the squared differences between successive NN intervals. |
|  | Proportion of normal-to-normal intervals (pNN) | 7 | The percentage of successive NN intervals that differ by more than a specific threshold (e.g., pNN50 for >50 ms), reflecting heart rate variability (HRV). |
|  | Mean RR interval (mean RRI) | 6 | The average time interval between consecutive R-wave peaks on an ECG, measured in milliseconds. |
|  | Standard deviation of heart rate (SD of HR) | 3 | The standard deviation of heart rate values over a given time period. |
| **Frequency domain** | High-frequency (HF) | 14 | Reflects parasympathetic nervous system activity and is associated with respiration-related HRV, typically measured in the range of 0.15–0.4 Hz. |
|  | Low-frequency (LF) | 12 | Represents both sympathetic and parasympathetic nervous system activities, measured in the frequency range of 0.04–0.15 Hz. |
|  | Low-frequency to high-frequency power ratio (LF/HF ratio) | 13 | The ratio of low-frequency to high-frequency power, indicating the balance between sympathetic and parasympathetic nervous system activity. |
|  | Very low frequency (VLF) | 7 | Reflects long-term regulatory mechanisms of the heart, typically measured in the range of 0.0033–0.04 Hz. |
|  | Ultra-low frequency (ULF) | 4 | Reflects extremely slow regulatory mechanisms, typically measured in frequencies <0.003 Hz. |
|  | Total power (TP) | 5 | Represents the total variance in HRV over all frequency components, reflecting overall autonomic nervous system activity. |
| **4. Smartphone parameters** | | | |
| Phone usage frequency | | 17 | The number of times a phone is used during a specified period. |
| Phone usage duration | | 7 | The total time spent using a phone during a specified period, measured in minutes or hours. |
| Phone calls | | 8 | The number of phone calls made or received during a specified period. |
| Light exposure | | 6 | The amount of light detected by the smartphone’s sensors, representing the user’s environment. |
| Number of Bluetooth-connected devices | | 4 | The count of unique Bluetooth devices detected within the range of the smartphone. |
| Typing pattern | | 2 | The characteristics of typing behavior on a touchscreen smartphone, such as speed or rhythm. |
| **5. Speech parameters** | | | |
| **Speech flow parameter** | Speech rate | 8 | The number of words spoken per minute, calculated based on the duration of speech. |
|  | Speech duration | 6 | The total time spent speaking during a recording or conversation, measured in seconds or minutes. |
|  | Pause time | 6 | The duration of silence between speech segments, reflecting pauses in communication. |
| **Voice acoustic parameters** | Mel-frequency cepstral coefficients (MFCCs) | 7 | Acoustic features representing the energy distribution and frequency characteristics of voice. |
|  | Fundamental frequency (F0) | 6 | The lowest frequency of a sound wave, representing the pitch of the voice. |
|  | Jitter | 4 | Variations in the fundamental frequency of a voice signal between consecutive vocal cycles. |
|  | Shimmer | 4 | Variations in the amplitude (loudness) of the voice signal between consecutive cycles. |
| **6. GPS parameters** | | | |
| Total distance | | 13 | The total distance traveled by the individual during a specific period. |
| Location variance | | 11 | The variability in the distances between different locations visited. |
| Time spent at home | | 10 | The total duration an individual spends at their home location during a specific period. |
| Location entropy | | 7 | A measure of the diversity of locations visited, indicating how uniformly time is distributed across places. |
| Normalized location entropy | | 5 | Location entropy normalized by the total number of visited places or maximum possible entropy. |
| Number of locations visited | | 6 | The total count of unique locations visited during a specific period. |
| Average moving speed | | 6 | The average speed of movement during active travel. |
| Time spent in moving | | 5 | The total duration of time spent actively moving, such as walking, driving, or cycling. |
| **7. Circadian rhythm** | | | |
| Interdaily stability (IS) | | 8 | A measure of the consistency of circadian rhythms across days, ranging from 0 (no stability) to 1 (perfect stability). |
| Intradaily variability (IV) | | 7 | A measure of the fragmentation or variability of activity patterns in a single day. |
| Midline estimating statistic of rhythm (MESOR) | | 7 | The average level of a rhythm over a 24-h period, often used as the baseline in circadian rhythm studies. |
| Amplitude | | 7 | The difference between the peak and the average level (MESOR) of the circadian rhythm. |
| Acrophase, peak | | 7 | The time of day when the rhythm reaches its peak level, typically expressed in hours (hh: mm). |
| Relative amplitude (RA) | | 4 | A ratio comparing the difference between the most active and least active periods to the total activity, reflecting rhythm robustness. |
| Pseudo F-statistic (F-pseudo) | | 4 | A statistical measure used to assess the significance of rhythmicity in circadian rhythm studies. |
| Most active 10-h period (M10) | | 3 | The period of 10 consecutive hours with the highest activity levels within a day. |
| Least active 5-h period (L5) | | 2 | The period of 5 consecutive hours with the lowest activity levels within a day. |

**Table S4. Study Quality Assessment using SIGN Methodology Checklist 4: Case-control Studies**

| **SIGN Methodology Checklist 4: Case-control studies** | | | | | | | | | | | | | | | |
| --- | --- | --- | --- | --- | --- | --- | --- | --- | --- | --- | --- | --- | --- | --- | --- |
| 1.1 | The study addresses an appropriate and clearly focused question. | | | | | | | | | | | | | | |
| 1.2 | The cases and controls are taken from comparable populations. | | | | | | | | | | | | | | |
| 1.3 | The same exclusion criteria are used for both cases and controls. | | | | | | | | | | | | | | |
| 1.4 | What percentage of each group (cases and controls) participated in the study? | | | | | | | | | | | | | | |
| 1.5 | Comparison is made between participants and non-participants to establish their similarities or differences. | | | | | | | | | | | | | | |
| 1.6 | Cases are clearly defined and differentiated from controls. | | | | | | | | | | | | | | |
| 1.7 | It is clearly established that controls are non-cases. | | | | | | | | | | | | | | |
| 1.8 | Measures will have been taken to prevent knowledge of primary exposure influencing case ascertainment. | | | | | | | | | | | | | | |
| 1.9 | Exposure status is measured in a standard, valid, and reliable way. | | | | | | | | | | | | | | |
| 1.10 | The main potential confounders are identified and taken into account in the design and analysis. | | | | | | | | | | | | | | |
| 1.11 | Confidence intervals are provided. | | | | | | | | | | | | | | |
| 2.1 | How well was the study done to minimise the risk of bias or confounding? | | | | | | | | | | | | | | |
| 2.2 | Taking into account clinical considerations, your evaluation of the methodology used, and the statistical power of the study, do you think there is clear evidence of an association between exposure and outcome? | | | | | | | | | | | | | | |
| 2.3 | Are the results of this study directly applicable to the patient group targeted by this guideline? | | | | | | | | | | | | | | |
| First Author | Year | **Internal validity** | **Selection of subjects** | | | | | | **Evaluation** | | **Confounding** | **Statistical analysis** | **Overall assessment** | | |
|  |  | **1.1** | **1.2** | **1.3** | **1.4** | **1.5** | **1.6** | **1.7** | **1.8** | **1.9** | **1.10** | **1.11** | **2.1** | **2.2** | **2.3** |
| Poon [1] | 2024 | Yes | Yes | Yes | 71.5, 77.1 | No | Yes | Yes | Yes | Yes | Yes | Yes | ++ | Yes | Yes |
| Comas [3] | 2023 | Yes | Yes | Yes | 85.2 | No | Yes | No | Yes | Yes | Yes | Yes | ++ | Yes | Yes |
| Bowman [4] | 2021 | Yes | No | Yes | 81.6 | Yes | Yes | Yes | Yes | Yes | Yes | Yes | ++ | Yes | Yes |
| Smith [5] | 2021 | Yes | No | Yes | 36.7 | No | No | No | Yes | Yes | Yes | Yes | ++ | Yes | Yes |
| Mohammadi [6] | 2021 | Yes | Yes | Yes | 100 | No | Yes | No | Yes | Yes | Yes | Yes | ++ | Yes | Yes |
| Zhang [7] | 2021 | Yes | Yes | Yes | 61.3 | No | Yes | Yes | Yes | Yes | Yes | Yes | ++ | Yes | Yes |
| Chen [8] | 2020 | Yes | Yes | Yes | 100 | No | Yes | No | Yes | Yes | Yes | Yes | ++ | Yes | Yes |
| Rajpurkar [10] | 2020 | Yes | No | Yes | 51.4 | No | No | No | Yes | Yes | No | Yes | + | Yes | Yes |
| Gould [12] | 2018 | Yes | Yes | Yes | 100 | No | Yes | No | Yes | Yes | Yes | Yes | ++ | Yes | Yes |
| Haghighi [13] | 2017 | Yes | Yes | Yes | 100 | No | Yes | No | Yes | Yes | Yes | Yes | ++ | Yes | Yes |
| Lee [14] | 2014 | Yes | Yes | Yes | 81 | No | Yes | Yes | Yes | Yes | No | Yes | + | Yes | Yes |
| Maglione [15] | 2014 | Yes | Yes | Yes | 20.1 | No | Yes | No | Yes | Yes | Yes | Yes | ++ | Yes | Yes |
| Pillai [16] | 2014 | Yes | No | Yes | 92.8 | No | No | No | Yes | Yes | Yes | Yes | + | Yes | No |
| Maglione [17] | 2012 | Yes | Yes | Yes | 64.4 | No | Yes | Yes | Yes | Yes | Yes | Yes | ++ | Yes | Yes |
| Bei [18] | 2010 | Yes | Yes | Yes | 63.6 | No | Yes | No | Yes | Yes | Yes | Yes | + | Yes | Yes |
| Goyal [19] | 2009 | Yes | No | Yes | 100 | No | No | No | Yes | Yes | Yes | Yes | ++ | Yes | No |
| Coffield [20] | 2004 | Yes | No | Can't say | NS | No | Yes | Yes | Yes | Yes | Can't say | No | + | Can't say | No |
| Nofzinger [21] | 1999 | Yes | Yes | Yes | NS | No | Yes | Yes | Yes | Yes | Yes | Yes | ++ | Yes | Yes |
| Luthringer [22] | 1996 | Yes | Yes | Yes | NS | No | Yes | Yes | Yes | Yes | Yes | Yes | ++ | Yes | Yes |
| Roemer [23] | 1992 | Yes | Yes | Yes | 100 | No | Yes | No | Yes | Yes | Yes | Yes | ++ | Yes | Yes |
| Theofilis [24] | 2023 | Yes | Yes | Yes | 80.1 | No | Yes | Yes | Yes | Yes | Yes | Yes | ++ | Yes | Yes |
| Siddi [25] | 2023 | Yes | Yes | Yes | NS | No | Yes | Can't say | Yes | Yes | Yes | Yes | + | Yes | Yes |
| Subramaniam [26] | 2023 | Yes | Yes | Yes | 100 | No | Yes | No | Yes | Yes | Yes | Yes | ++ | Yes | Yes |
| Lee [27] | 2022 | Yes | Yes | Yes | 80.9 | No | Yes | Yes | Yes | Yes | Yes | Yes | ++ | Yes | Yes |
| Chung [28] | 2021 | Yes | No | Yes | 62.8 | No | No | No | Yes | Yes | Yes | Yes | ++ | Yes | Yes |
| da Estrela [29] | 2021 | Yes | Yes | Yes | High participation rate | No | Yes | Yes | Yes | Yes | Yes | Yes | ++ | Yes | Yes |
| Lee [30] | 2021 | Yes | Yes | Yes | 100 | No | Yes | No | Yes | Yes | Yes | Yes | ++ | Yes | Yes |
| Huang [32] | 2018 | Yes | Yes | Yes | 88 | No | Yes | Yes | Yes | Yes | Yes | Yes | ++ | Yes | Yes |
| Hamilton [33] | 2017 | Yes | Yes | Yes | 100 | No | Yes | No | Yes | Yes | Yes | Yes | + | Yes | Yes |
| Sun [34] | 2016 | Yes | Yes | Yes | 100 | No | Yes | Yes | Yes | Yes | Yes | Yes | ++ | Yes | Yes |
| Liang [35] | 2015 | Yes | Yes | Yes | 97.6 | No | Yes | Yes | Yes | Yes | Yes | Yes | ++ | Yes | Yes |
| Lee [37] | 2012 | Yes | No | Yes | 100 | No | No | No | Yes | Yes | No | Yes | + | Yes | Yes |
| Licht [39] | 2008 | Yes | Yes | Yes | 79.6 | No | Yes | Yes | Yes | Yes | Yes | Yes | ++ | Yes | Yes |
| Kojima [40] | 2008 | Yes | Yes | Yes | 47.8 | No | Yes | No | Yes | Yes | Yes | Yes | + | Yes | Yes |
| Stroup-Benham [41] | 2000 | Yes | Yes | Yes | 90 | No | Yes | No | Yes | Yes | Yes | Yes | ++ | Yes | Yes |
| Stein [42] | 2000 | Yes | Yes | Yes | 74.5 | No | Yes | Yes | Yes | Yes | Yes | Yes | ++ | Yes | Yes |
| Wang [43] | 2023 | Yes | Yes | Yes | 33.6 | No | Yes | No | Yes | Yes | Yes | Yes | + | Yes | Yes |
| Hsiao [45] | 2022 | Yes | Yes | Yes | 61.4 | No | Yes | No | Yes | Yes | Yes | Yes | + | Yes | Yes |
| Ramsey [46] | 2022 | Yes | Yes | Yes | 71.7 | No | Yes | No | Yes | Yes | Yes | Yes | + | Yes | Yes |
| Hussenoeder [47] | 2022 | Yes | Yes | Yes | 65.8 | No | Yes | Yes | Yes | Yes | Yes | Yes | ++ | Yes | Yes |
| Michalak [48] | 2022 | Yes | Yes | Yes | 100 | No | Yes | Yes | Yes | Yes | Yes | Yes | ++ | Yes | Yes |
| Li [49] | 2022 | Yes | Yes | Yes | 100 | No | Yes | No | Yes | Yes | Yes | Yes | ++ | Yes | Yes |
| Figueroa [50] | 2021 | Yes | Yes | Yes | 86.2 | No | Yes | Yes | Yes | Yes | Yes | Yes | ++ | Yes | Yes |
| Averill [54] | 2018 | Yes | No | Yes | 61.5 | No | No | No | Yes | Yes | No | Yes | + | Yes | Yes |
| Holmquist [56] | 2017 | Yes | No | Yes | 96 | No | No | No | Yes | Yes | Yes | Yes | ++ | Yes | Yes |
| Todder [57] | 2009 | Yes | Yes | Yes | 100 | Yes | Yes | Yes | Yes | Yes | Yes | Yes | ++ | Yes | No |
| Lemke [58] | 2000 | Yes | Yes | Yes | NS | No | Yes | Yes | Yes | Yes | Yes | Yes | ++ | Yes | Yes |
| Wadle [69] | 2024 | Yes | Yes | Yes | 73 | No | Yes | No | Yes | Yes | Yes | Yes | ++ | Yes | Yes |
| Silva [70] | 2024 | Yes | Yes | Yes | 100 | No | Yes | Yes | Yes | Yes | Yes | Yes | ++ | Yes | Yes |
| Wang [72] | 2023 | Yes | Yes | Yes | 100 | No | Yes | No | Yes | Yes | Yes | Yes | ++ | Yes | Yes |
| Ali [78] | 2023 | Yes | Yes | Yes | 51.9 | No | Yes | Yes | Yes | Yes | Yes | Yes | + | Yes | Yes |
| Braund [79] | 2022 | Yes | No | Yes | 74.6 | No | Yes | No | Yes | Yes | Can't say | Yes | + | No | Yes |
| George [81] | 2021 | Yes | Yes | Yes | 85.2 | No | Yes | Yes | Yes | Yes | Yes | Yes | ++ | Yes | Yes |
| Smagula [82] | 2015 | Yes | Yes | Yes | 92.2 | No | Yes | Yes | Yes | Yes | Yes | Yes | ++ | Yes | Yes |
| Maglione [83] | 2014 | Yes | Yes | Yes | 63.9 | No | Yes | No | Yes | Yes | Yes | Yes | ++ | Yes | Yes |
| Luik [84] | 2013 | Yes | No | Yes | 78 | No | No | No | Yes | Yes | Yes | Yes | ++ | Yes | Yes |
| Bae [90] | 2023 | Yes | Yes | Yes | 57.1 | No | Yes | No | Yes | Yes | Yes | Yes | + | Yes | Yes |
| Mishra [92] | 2021 | Yes | No | Yes | 100 | No | No | No | Yes | Yes | No | Yes | + | Yes | No |
| Raoux [93] | 1994 | Yes | Yes | Yes | 100 | No | Yes | No | Yes | Yes | Yes | Yes | + | Yes | Yes |
| Sayar [95] | 2002 | Yes | Yes | Yes | 100 | No | Yes | Yes | Yes | Yes | Yes | Yes | + | Yes | Yes |
| Landolt [96] | 2005 | Yes | Yes | Yes | 100 | No | Yes | Yes | Yes | Yes | Yes | Yes | ++ | Yes | Yes |
| Hoyos [97] | 2020 | Yes | Yes | Yes | 80 | No | No | No | Yes | Yes | Yes | Yes | ++ | Yes | Yes |
| Lorenz [98] | 2019 | Yes | Yes | Yes | 100 | No | Yes | Yes | Yes | Yes | Yes | Yes | ++ | Yes | Yes |
| Vanderlind [99] | 2014 | Yes | No | Yes | 67 | No | No | No | Yes | Yes | No | Yes | + | Yes | No |
| Di Matteo [109] | 2020 | Yes | Yes | Yes | 41 | No | Yes | No | Yes | Yes | Yes | Yes | + | Yes | Yes |
| Shah [112] | 2021 | Yes | No | Yes | 100 | No | No | No | Yes | Yes | Yes | Yes | + | Yes | No |
| Kumagai [116] | 2019 | Yes | Yes | Yes | 89 | No | Yes | No | Yes | Yes | Yes | Yes | ++ | Yes | Yes |
| Funkhouser [117] | 2025 | Yes | Yes | Yes | 64.4 | Yes | Yes | No | Yes | Yes | Yes | Yes | + | Yes | Yes |
| Ben-Zeev [120] | 2015 | Yes | No | Yes | 78.7 | No | No | No | Yes | Yes | No | Yes | + | Yes | No |
| Moshe [126] | 2021 | Yes | Yes | Yes | 91.7 | No | Yes | Yes | Yes | Yes | Yes | Yes | ++ | Yes | Yes |
| Wang [127] | 2014 | Yes | Yes | Yes | 80 | No | Yes | Yes | Yes | Yes | Yes | Yes | ++ | Yes | Yes |
| Song [128] | 2024 | Yes | Yes | Yes | 64.1 | No | Yes | No | Yes | Yes | Yes | Yes | + | Yes | Yes |
| Aledavood [132] | 2025 | Yes | Yes | Yes | 92.1 | No | Yes | No | Yes | Yes | Yes | Yes | + | Yes | Yes |

**References**

1. Poon CY, Cheng YC, Wong VWH, et al. Directional associations among real-time activity, sleep, mood, and daytime symptoms in major depressive disorder using actigraphy and ecological momentary assessment. Behav Res Ther. Feb 2024;173:104464. [doi: 10.1016/j.brat.2023.104464] [Medline: 38159415]
2. Peng D, Liu W, Luo Y, Mao Z, Zheng WL, Lu BL. Deep depression detection with resting-state and cognitive-task EEG. Annu Int Conf IEEE Eng Med Biol Soc. Jul 2023;2023:1-4. [doi: 10.1109/EMBC40787.2023.10340667] [Medline: 38083722]
3. Comas M, Solis Flores A, Lovato N, et al. The relationship between anxiety, subjective and objective sleep, chronotype and circadian rhythms with depressive symptoms in insomnia disorder. Brain Sci. Apr 4, 2023;13(4):613. [doi: 10.3390/ brainsci13040613] [Medline: 37190578]
4. Bowman MA, Kline CE, Buysse DJ, et al. Longitudinal association between depressive symptoms and multidimensional sleep health: the SWAN Sleep Study. Ann Behav Med. Jun 28, 2021;55(7):641-652. [doi: 10.1093/abm/kaaa107] [Medline: 33410460]
5. Smith P, Kandakatla A, Frankel CW, et al. Sleep quality, depressive symptoms, and transplant outcomes: follow-up analyses from the ADAPT prospective pilot study. Gen Hosp Psychiatry. 2021;72:53-58. [doi: 10.1016/j.genhosppsych. 2021.06.011] [Medline: 34298477]
6. Mohammadi Y, Moradi MH. Prediction of depression severity scores based on functional connectivity and complexity of the EEG signal. Clin EEG Neurosci. Jan 2021;52(1):52-60. [doi: 10.1177/1550059420965431] [Medline: 33040603]
7. Zhang Y, Folarin AA, Sun S, et al. Predicting depressive symptom severity through individuals’ nearby Bluetooth device count data collected by mobile phones: preliminary longitudinal study. JMIR Mhealth Uhealth. Jul 30, 2021;9(7):e29840. [doi: 10.2196/29840] [Medline: 34328441]
8. Chen F, Zhao L, Li B, Yang L. Depression evaluation based on prefrontal EEG signals in resting state using fuzzy measure entropy. Physiol Meas. Oct 6, 2020;41(9):095007. [doi: 10.1088/1361-6579/abb144] [Medline: 33021227]
9. Hasanzadeh F, Mohebbi M, Rostami R. Graph theory analysis of directed functional brain networks in major depressive disorder based on EEG signal. J Neural Eng. Mar 27, 2020;17(2):026010. [doi: 10.1088/1741-2552/ab7613] [Medline: 32053813]
10. Rajpurkar P, Yang J, Dass N, et al. Evaluation of a machine learning model based on pretreatment symptoms and electroencephalographic features to predict outcomes of antidepressant treatment in adults with depression: a prespecified secondary analysis of a randomized clinical trial. JAMA Netw Open. Jun 1, 2020;3(6):e206653. [doi: 10. 1001/jamanetworkopen.2020.6653] [Medline: 32568399]
11. Ding X, Yue X, Zheng R, Bi C, Li D, Yao G. Classifying major depression patients and healthy controls using EEG, eye tracking and galvanic skin response data. J Affect Disord. May 15, 2019;251:156-161. [doi: 10.1016/j.jad.2019.03.058] [Medline: 30925266]
12. Gould CE, Karna R, Jordan J, et al. Subjective but not objective sleep is associated with subsyndromal anxiety and depression in community-dwelling older adults. Am J Geriatr Psychiatry. Jul 2018;26(7):806-811. [doi: 10.1016/j.jagp. 2018.03.010] [Medline: 29709510]
13. Haghighi M, Ludyga S, Rahimi B, et al. In patients suffering from major depressive disorders, quantitative EEG showed favorable changes in left and right prefrontal cortex. Psychiatry Res. May 2017;251:137-141. [doi: 10.1016/j.psychres. 2017.02.012] [Medline: 28199912]
14. Lee HJ, Lee JS, Kim T, Yoon IY. Relationship between sleep disturbances and cognitive impairments in older adults with depression. Sleep Medicine and Psychophysiology. Jun 30, 2014;21(1):5-13. [doi: 10.14401/KASMED.2014.21.1. 5]
15. Maglione JE, Ancoli-Israel S, Peters KW, et al. Subjective and objective sleep disturbance and longitudinal risk of depression in a cohort of older women. Sleep. Jul 1, 2014;37(7):1179-1187. [doi: 10.5665/sleep.3834] [Medline: 25061246]
16. Pillai V, Steenburg LA, Ciesla JA, Roth T, Drake CL. A seven day actigraphy-based study of rumination and sleep disturbance among young adults with depressive symptoms. J Psychosom Res. Jul 2014;77(1):70-75. [doi: 10.1016/j. jpsychores.2014.05.004] [Medline: 24913345]
17. Maglione JE, Ancoli-Israel S, Peters KW, et al. Depressive symptoms and subjective and objective sleep in communitydwelling older women. J Am Geriatr Soc. Apr 2012;60(4):635-643. [doi: 10.1111/j.1532-5415.2012.03908.x] [Medline: 22428562]
18. Bei B, Milgrom J, Ericksen J, Trinder J. Subjective perception of sleep, but not its objective quality, is associated with immediate postpartum mood disturbances in healthy women. Sleep. Apr 2010;33(4):531-538. [doi: 10.1093/sleep/33.4. 531] [Medline: 20394323]
19. Goyal D, Gay C, Lee K. Fragmented maternal sleep is more strongly correlated with depressive symptoms than infant temperament at three months postpartum. Arch Womens Ment Health. Aug 2009;12(4):229-237. [doi: 10.1007/s00737- 009-0070-9] [Medline: 19396527]
20. Coffield TG, Tryon WW. Construct validation of actigraphic sleep measures in hospitalized depressed patients. Behav Sleep Med. 2004;2(1):24-40. [doi: 10.1207/s15402010bsm0201_3] [Medline: 15600222]
21. Nofzinger EA, Nichols TE, Meltzer CC, et al. Changes in forebrain function from waking to REM sleep in depression: preliminary analyses [of 18F]FDG PET studies. Psychiatry Research: Neuroimaging. Aug 1999;91(2):59-78. [doi: 10. 1016/S0925-4927(99)00025-6]
22. Luthringer R, Toussaint M, Schaltenbrand N, et al. A double-blind, placebo-controlled evaluation of the effects of orally administered venlafaxine on sleep in inpatients with major depression. Psychopharmacol Bull. 1996;32(4):637-646. [Medline: 8993085]
23. Roemer RA, Shagass C, Dubin W, Jaffe R, Siegal L. Quantitative EEG in elderly depressives. Brain Topogr. 1992;4(4):285-290. [doi: 10.1007/BF01135566] [Medline: 1510872]
24. Theofilis P, Oikonomou E, Lazaros G, et al. The association of depression with QT duration: a comparison between individuals younger or older than 65 years. Psychosom Med. Jan 1, 2023;85(1):18-25. [doi: 10.1097/PSY. 0000000000001152] [Medline: 36516316]
25. Siddi S, Bailon R, Giné-Vázquez I, et al. The usability of daytime and night-time heart rate dynamics as digital biomarkers of depression severity. Psychol Med. Jun 2023;53(8):3249-3260. [doi: 10.1017/S0033291723001034] [Medline: 37184076]
26. Subramaniam V, R N. Relationship between heart rate variability and major depressive disorder in young adults. Swiss Arch Neurol Psychiatr Psychother. 2023;174:126-129. [doi: 10.4414/sanp.2023.03284]
27. Lee SY, Waring ME, Park CL, Blake EC. Do depressive symptoms predict blood pressure control in US veterans? J Gen Intern Med. Jan 2022;37(1):57-63. [doi: 10.1007/s11606-021-06709-5] [Medline: 33772439]
28. Chung AH, Gevirtz RN, Gharbo RS, Thiam MA, Ginsberg JPJ. Pilot study on reducing symptoms of anxiety with a heart rate variability biofeedback wearable and remote stress management coach. Appl Psychophysiol Biofeedback. Dec 2021;46(4):347-358. [doi: 10.1007/s10484-021-09519-x] [Medline: 34308526]
29. da Estrela C, McGrath J, Booij L, Gouin JP. Heart rate variability, sleep quality, and depression in the context of chronic stress. Ann Behav Med. Mar 16, 2021;55(2):155-164. [doi: 10.1093/abm/kaaa039] [Medline: 32525208]
30. Lee MS, Seo YE, Mok YE, Lee SH. Heart rate variability after treatment for depression in North Korean defectors. Appl Psychophysiol Biofeedback. Mar 2021;46(1):11-18. [doi: 10.1007/s10484-020-09491-y] [Medline: 33074464]
31. Coutts LV, Plans D, Brown AW, Collomosse J. Deep learning with wearable based heart rate variability for prediction of mental and general health. J Biomed Inform. Dec 2020;112(112):103610. [doi: 10.1016/j.jbi.2020.103610] [Medline: 33137470]
32. Huang M, Shah A, Su S, et al. Association of depressive symptoms and heart rate variability in Vietnam war-era twins: a longitudinal twin difference study. JAMA Psychiatry. Jul 1, 2018;75(7):705-712. [doi: 10.1001/jamapsychiatry.2018. 0747] [Medline: 29799951]
33. Hamilton JL, Alloy LB. Physiological markers of interpersonal stress generation in depression. Clin Psychol Sci. 2017;5(6):911-929. [doi: 10.1177/2167702617720211] [Medline: 29123970]
34. Sun G, Shinba T, Kirimoto T, Matsui T. An objective screening method for major depressive disorder using logistic regression analysis of heart rate variability data obtained in a mental task paradigm. Front Psychiatry. 2016;7:180. [doi: 10.3389/fpsyt.2016.00180] [Medline: 27867364]
35. Liang CS, Lee JF, Chen CC, Chang YC. Reactive heart rate variability in male patients with first-episode major depressive disorder. Prog Neuropsychopharmacol Biol Psychiatry. Jan 2, 2015;56:52-57. [doi: 10.1016/j.pnpbp.2014.08. 004] [Medline: 25149628]
36. Roh T, Hong S, Yoo HJ. Wearable depression monitoring system with heart-rate variability. Annu Int Conf IEEE Eng Med Biol Soc. 2014;2014:562-565. [doi: 10.1109/EMBC.2014.6943653] [Medline: 25570021]
37. Lee CK, Yoo SK. An analysis of the relationship between self-reported anxiety, depressiveness and parameters of heart rate variability based on photoplethysmography [Article in Korean]. Sci Emot Sens. 2012;3:345-354. URL: https:// koreascience.kr/article/JAKO201229665547045.pdf [Accessed 2026-03-21]
38. Zhang ZX, Tian XW, Lim JS. Neuro-fuzzy network-based depression diagnosis algorithm using optimal features of HRV. The Journal of the Korea Contents Association. Feb 28, 2012;12(2):1-9. [doi: 10.5392/JKCA.2012.12.02.001]
39. Licht CMM, de Geus EJC, Zitman FG, Hoogendijk WJG, van Dyck R, Penninx B. Association between major depressive disorder and heart rate variability in the Netherlands Study of Depression and Anxiety (NESDA). Arch Gen Psychiatry. Dec 2008;65(12):1358-1367. [doi: 10.1001/archpsyc.65.12.1358] [Medline: 19047522]
40. Kojima M, Hayano J, Fukuta H, et al. Loss of fractal heart rate dynamics in depressive hemodialysis patients. Psychosom Med. Feb 2008;70(2):177-185. [doi: 10.1097/PSY.0b013e31816477a1] [Medline: 18256338]
41. Stroup-Benham CA, Markides KS, Black SA, Goodwin JS. Relationship between low blood pressure and depressive symptomatology in older people. J Am Geriatr Soc. Mar 2000;48(3):250-255. [doi: 10.1111/j.1532-5415.2000.tb02642. x] [Medline: 10733049]
42. Stein PK, Carney RM, Freedland KE, et al. Severe depression is associated with markedly reduced heart rate variability in patients with stable coronary heart disease. J Psychosom Res. 2000;48(4-5):493-500. [doi: 10.1016/s0022- 3999(99)00085-9] [Medline: 10880671]
43. Wang X, Pathiravasan CH, Zhang Y, et al. Association of depressive symptom trajectory with physical activity collected by mHealth devices in the Electronic Framingham Heart Study: cohort study. JMIR Ment Health. Jul 14, 2023;10:e44529. [doi: 10.2196/44529] [Medline: 37450333]
44. Espino-Salinas CH, Galván-Tejada CE, Luna-García H, et al. Two-dimensional convolutional neural network for depression episodes detection in real time using motor activity time series of Depresjon dataset. Bioengineering (Basel). Sep 9, 2022;9(9):458. [doi: 10.3390/bioengineering9090458] [Medline: 36135004]
45. Hsiao C, Hsueh MC, Liao Y. Associations between objectively measured sedentary behavior patterns and depressive symptoms in older adults: a cross sectional study. Ment Health Phys Act. Oct 2022;23:100471. [doi: 10.1016/j.mhpa. 2022.100471]
46. Ramsey CM, Lynch KG, Gehrman PR, et al. Daily steps and depressive symptoms: a longitudinal evaluation of patients with major depressive disorder in the precision medicine in mental health care study. J Affect Disord. Mar 1, 2022;300:334-340. [doi: 10.1016/j.jad.2021.12.116] [Medline: 34979178]
47. Hussenoeder FS, Conrad I, Pabst A, et al. Physical activity and mental health: the connection between step count and depression, anxiety and quality of sleep. Psychol Health Med. 2023;28(9):2419-2429. [doi: 10.1080/13548506.2022. 2159453] [Medline: 36529963]
48. Michalak J, Niemeyer H, Tschacher W, Baumann N, Chi Zhang X, Adolph D. Subjective and objective measures of activity in depressed and non-depressed individuals in everyday life. J Exp Psychopathol. Apr 2022;13(2):20438087221092582. [doi: 10.1177/20438087221092582]
49. Li YM, Konstabel K, Mõttus R, Lemola S. Temporal associations between objectively measured physical activity and depressive symptoms: an experience sampling study. Front Psychiatry. 2022;13:920580. [doi: 10.3389/fpsyt.2022. 920580] [Medline: 35923450]
50. Figueroa CA, Vittinghoff E, Aguilera A, Fukuoka Y. Differences in objectively measured daily physical activity patterns related to depressive symptoms in community dwelling women - mPED trial. Prev Med Rep. Jun 2021;22:101325. [doi: 10.1016/j.pmedr.2021.101325] [Medline: 33659156]
51. Hsueh MC, Stubbs B, Lai YJ, Sun CK, Chen LJ, Ku PW. A dose response relationship between accelerometer assessed daily steps and depressive symptoms in older adults: a two-year cohort study. Age Ageing. Feb 26, 2021;50(2):519-526. [doi: 10.1093/ageing/afaa162] [Medline: 32980870]
52. Jakobsen P, Garcia-Ceja E, Riegler M, et al. Applying machine learning in motor activity time series of depressed bipolar and unipolar patients compared to healthy controls. PLoS ONE. 2020;15(8):e0231995. [doi: 10.1371/journal. pone.0231995] [Medline: 32833958]
53. Zhao N, Zhang Z, Wang Y, et al. See your mental state from your walk: recognizing anxiety and depression through Kinect-recorded gait data. PLoS ONE. 2019;14(5):e0216591. [doi: 10.1371/journal.pone.0216591] [Medline: 31116785]
54. Averill IR, Crowe M, Frampton CM, et al. Clinical response to treatment in inpatients with depression correlates with changes in activity levels and psychomotor speed. Aust N Z J Psychiatry. Jul 2018;52(7):652-659. [doi: 10.1177/ 0004867417753549] [Medline: 29417833]
55. Ku PW, Steptoe A, Liao Y, Sun WJ, Chen LJ. Prospective relationship between objectively measured light physical activity and depressive symptoms in later life. Int J Geriatr Psychiatry. Jan 2018;33(1):58-65. [doi: 10.1002/gps.4672] [Medline: 28181713]
56. Holmquist S, Mattsson S, Schele I, Nordström P, Nordström A. Low physical activity as a key differentiating factor in the potential high-risk profile for depressive symptoms in older adults. Depress Anxiety. Sep 2017;34(9):817-825. [doi: 10.1002/da.22638] [Medline: 28489319]
57. Todder D, Caliskan S, Baune BT. Longitudinal changes of day-time and night-time gross motor activity in clinical responders and non-responders of major depression. World J Biol Psychiatry. 2009;10(4):276-284. [doi: 10.3109/ 15622970701403081] [Medline: 19921969]
58. Lemke MR, Wendorff T, Mieth B, Buhl K, Linnemann M. Spatiotemporal gait patterns during over ground locomotion in major depression compared with healthy controls. J Psychiatr Res. 2000;34(4-5):277-283. [doi: 10.1016/s0022- 3956(00)00017-0] [Medline: 11104839]
59. Fadul R, Alfalahi H, Shehhi AA, Hadjileontiadis L. Depressive disorder remote detection through touchscreen typing behaviour. Annu Int Conf IEEE Eng Med Biol Soc. Jul 2023;2023:1-4. [doi: 10.1109/EMBC40787.2023.10340393] [Medline: 38082634]
60. Auerbach RP, Srinivasan A, Kirshenbaum JS, Mann JJ, Shankman SA. Geolocation features differentiate healthy from remitted depressed adults. J Psychopathol Clin Sci. May 2022;131(4):341-349. [doi: 10.1037/abn0000742] [Medline: 35230855]
61. Otte Andersen T, Skovlund Dissing A, Rosenbek Severinsen E, et al. Predicting stress and depressive symptoms using high-resolution smartphone data and sleep behavior in Danish adults. Sleep. Jun 13, 2022;45(6):zsac067. [doi: 10.1093/ sleep/zsac067] [Medline: 35298650]
62. Opoku Asare K, Terhorst Y, Vega J, Peltonen E, Lagerspetz E, Ferreira D. Predicting depression from smartphone behavioral markers using machine learning methods, hyperparameter optimization, and feature importance analysis: exploratory study. JMIR Mhealth Uhealth. Jul 12, 2021;9(7):e26540. [doi: 10.2196/26540] [Medline: 34255713]
63. Chikersal P, Doryab A, Tumminia M, et al. Detecting depression and predicting its onset using longitudinal symptoms captured by passive sensing: a machine learning approach with robust feature selection. ACM Trans Comput Hum Interact. 2021;28(1):1-41. [doi: 10.1145/3422821]
64. Zhang Y, Folarin AA, Sun S, et al. Relationship between major depression symptom severity and sleep collected using a wristband wearable device: multicenter longitudinal observational study. JMIR Mhealth Uhealth. Apr 12, 2021;9(4):e24604. [doi: 10.2196/24604] [Medline: 33843591]
65. Pedrelli P, Fedor S, Ghandeharioun A, et al. Monitoring changes in depression severity using wearable and mobile sensors. Front Psychiatry. 2020;11:584711. [doi: 10.3389/fpsyt.2020.584711] [Medline: 33391050]
66. Mastoras RE, Iakovakis D, Hadjidimitriou S, et al. Touchscreen typing pattern analysis for remote detection of the depressive tendency. Sci Rep. Sep 16, 2019;9(1):13414. [doi: 10.1038/s41598-019-50002-9] [Medline: 31527640]
67. Saeb S, Zhang M, Karr CJ, et al. Mobile phone sensor correlates of depressive symptom severity in daily-life behavior: an exploratory study. J Med Internet Res. Jul 15, 2015;17(7):e175. [doi: 10.2196/jmir.4273] [Medline: 26180009]
68. Wiseman M, Yep R, Wood Alexander M, et al. Objective speech measures capture depressive symptoms and associated cognitive difficulties. Transl Psychiatry. Nov 17, 2025;15(1):525. [doi: 10.1038/s41398-025-03728-2] [Medline: 41257835]
69. Wadle LM, Ebner-Priemer UW, Foo JC, et al. Speech features as predictors of momentary depression severity in patients with depressive disorder undergoing sleep deprivation therapy: ambulatory assessment pilot study. JMIR Ment Health. Jan 18, 2024;11:e49222. [doi: 10.2196/49222] [Medline: 38236637]
70. Silva WJ, Lopes L, Galdino MKC, Almeida AA. Voice acoustic parameters as predictors of depression. J Voice. Jan 2024;38(1):77-85. [doi: 10.1016/j.jvoice.2021.06.018] [Medline: 34353686]
71. Kim AY, Jang EH, Lee SH, Choi KY, Park JG, Shin HC. Automatic depression detection using smartphone-based textdependent speech signals: deep convolutional neural network approach. J Med Internet Res. Jan 25, 2023;25:e34474. [doi: 10.2196/34474] [Medline: 36696160]
72. Wang Y, Liang L, Zhang Z, et al. Fast and accurate assessment of depression based on voice acoustic features: a crosssectional and longitudinal study. Front Psychiatry. 2023;14:1195276. [doi: 10.3389/fpsyt.2023.1195276]
73. Zhao Q, Fan HZ, Li YL, et al. Vocal acoustic features as potential biomarkers for identifying/diagnosing depression: a cross-sectional study. Front Psychiatry. 2022;13:815678. [doi: 10.3389/fpsyt.2022.815678] [Medline: 35573349]
74. Ye J, Yu Y, Wang Q, et al. Multi-modal depression detection based on emotional audio and evaluation text. J Affect Disord. Dec 1, 2021;295:904-913. [doi: 10.1016/j.jad.2021.08.090] [Medline: 34706461]
75. Klangpornkun N, Ruangritchai M, Munthuli A, et al. Classification of depression and other psychiatric conditions using speech features extracted from a Thai psychiatric and verbal screening test. Annu Int Conf IEEE Eng Med Biol Soc. Nov 2021;2021:651-656. [doi: 10.1109/EMBC46164.2021.9629571] [Medline: 34891377]
76. Demiroglu C, Beşirli A, Ozkanca Y, Çelik S. Depression-level assessment from multi-lingual conversational speech data using acoustic and text features. J Audio Speech Music Proc. Dec 2020;2020(1):17. [doi: 10.1186/s13636-020-00182-4]
77. Yamamoto M, Takamiya A, Sawada K, et al. Using speech recognition technology to investigate the association between timing-related speech features and depression severity. PLoS ONE. 2020;15(9):e0238726. [doi: 10.1371/ journal.pone.0238726] [Medline: 32915846]
78. Ali FZ, Parsey RV, Lin S, Schwartz J, DeLorenzo C. Circadian rhythm biomarker from wearable device data is related to concurrent antidepressant treatment response. NPJ Digit Med. Apr 29, 2023;6(1):81. [doi: 10.1038/s41746-023- 00827-6] [Medline: 37120493]
79. Braund TA, Zin MT, Boonstra TW, et al. Smartphone sensor data for identifying and monitoring symptoms of mood disorders: a longitudinal observational study. JMIR Ment Health. May 4, 2022;9(5):e35549. [doi: 10.2196/35549] [Medline: 35507385]
80. Choi JG, Ko I, Han S. Depression level classification using machine learning classifiers based on actigraphy data. IEEE Access. 2021;9:116622-116646. [doi: 10.1109/ACCESS.2021.3105393]
81. George SV, Kunkels YK, Booij S, Wichers M. Uncovering complexity details in actigraphy patterns to differentiate the depressed from the non-depressed. Sci Rep. Jun 29, 2021;11(1):13447. [doi: 10.1038/s41598-021-92890-w] [Medline: 34188115]
82. Smagula SF, Ancoli-Israel S, Blackwell T, et al. Circadian rest-activity rhythms predict future increases in depressive symptoms among community-dwelling older men. Am J Geriatr Psychiatry. May 2015;23(5):495-505. [doi: 10.1016/j. jagp.2014.06.007] [Medline: 25066948]
83. Maglione JE, Ancoli-Israel S, Peters KW, et al. Depressive symptoms and circadian activity rhythm disturbances in community-dwelling older women. Am J Geriatr Psychiatry. Apr 2014;22(4):349-361. [doi: 10.1016/j.jagp.2012.09.003] [Medline: 23567424]
84. Luik AI, Zuurbier LA, Hofman A, Van Someren EJW, Tiemeier H. Stability and fragmentation of the activity rhythm across the sleep-wake cycle: the importance of age, lifestyle, and mental health. Chronobiol Int. Dec 2013;30(10):1223-1230. [doi: 10.3109/07420528.2013.813528] [Medline: 23971909]
85. Anik IA, Kamal AHM, Kabir MA, Uddin S, Moni MA. A robust deep-learning model to detect major depressive disorder utilizing EEG signals. IEEE Trans Artif Intell. 2024;5(10):4938-4947. [doi: 10.1109/TAI.2024.3394792]
86. Tian F, Zhang H, Tan Y, et al. An on-board executable multi-feature transfer-enhanced fusion model for three-lead EEG sensor-assisted depression diagnosis. IEEE J Biomed Health Inform. Jan 2025;29(1):152-165. [doi: 10.1109/JBHI.2024. 3487012] [Medline: 39466874]
87. Islam R, Bae SW. FacePsy: an open-source affective mobile sensing system - analyzing facial behavior and head gesture for depression detection in naturalistic settings. Proc ACM Hum-Comput Interact. Sep 24, 2024;8(MHCI):1-32. [doi: 10. 1145/3676505]
88. Makhmutova M, Kainkaryam R, Ferreira M, Min J, Jaggi M, Clay I. Predicting changes in depression severity using the PSYCHE-D (Prediction of Severity Change-Depression) model involving person-generated health data: longitudinal case-control observational study. JMIR Mhealth Uhealth. Mar 25, 2022;10(3):e34148. [doi: 10.2196/34148] [Medline: 35333186]
89. Price GD, Heinz MV, Collins AC, Jacobson NC. Detecting major depressive disorder presence using passively-collected wearable movement data in a nationally-representative sample. Psychiatry Res. Feb 2024;332:115693. [doi: 10.1016/j. psychres.2023.115693] [Medline: 38194801]
90. Bae S, Jang M, Kim GM, et al. Nonlinear associations between physical function, physical activity, sleep, and depressive symptoms in older adults. J Clin Med. Sep 16, 2023;12(18):6009. [doi: 10.3390/jcm12186009] [Medline: 37762948]
91. Griffiths C, da Silva KM, Leathlean C, Jiang H, Ang CS, Searle R. Investigation of physical activity, sleep, and mental health recovery in treatment resistant depression (TRD) patients receiving repetitive transcranial magnetic stimulation (rTMS) treatment. J Affect Disord Rep. Apr 2022;8:100337. [doi: 10.1016/j.jadr.2022.100337] [Medline: 35619990]
92. Mishra R, Park C, York MK, et al. Decrease in mobility during the COVID-19 pandemic and its association with increase in depression among older adults: a longitudinal remote mobility monitoring using a wearable sensor. Sensors (Basel). Apr 29, 2021;21(9):3090. [doi: 10.3390/s21093090] [Medline: 33946664]
93. Raoux N, Benoit O, Dantchev N, et al. Circadian pattern of motor activity in major depressed patients undergoing antidepressant therapy: relationship between actigraphic measures and clinical course. Psychiatry Res. Apr 1994;52(1):85-98. [doi: 10.1016/0165-1781(94)90122-8] [Medline: 8047624]
94. Rykov Y, Thach TQ, Bojic I, Christopoulos G, Car J. Digital biomarkers for depression screening with wearable devices: cross-sectional study with machine learning modeling. JMIR Mhealth Uhealth. Oct 25, 2021;9(10):e24872. [doi: 10.2196/24872] [Medline: 34694233]
95. Sayar K, Güleç H, Gökçe M, Ismail AK. Heart rate variability in depressed patients. Psychiatry Clin Psychopharmacol. 2002;12(3):130-133. URL: https://psychiatry-psychopharmacology.com/index.php/pub/article/view/313 [Accessed 2026-03-11]
96. Landolt HP, Gillin JC. Similar sleep EEG topography in middle-aged depressed patients and healthy controls. Sleep. Feb 2005;28(2):239-247. [doi: 10.1093/sleep/28.2.239] [Medline: 16171249]
97. Hoyos CM, Gordon C, Terpening Z, et al. Circadian rhythm and sleep alterations in older people with lifetime depression: a case-control study. BMC Psychiatry. Apr 29, 2020;20(1):192. [doi: 10.1186/s12888-020-02606-z] [Medline: 32349697]
98. Lorenz N, Spada J, Sander C, Riedel-Heller SG, Hegerl U. Circadian skin temperature rhythms, circadian activity rhythms and sleep in individuals with self-reported depressive symptoms. J Psychiatr Res. Oct 2019;117:38-44. [doi: 10. 1016/j.jpsychires.2019.06.022] [Medline: 31279242]
99. Vanderlind WM, Beevers CG, Sherman SM, et al. Sleep and sadness: exploring the relation among sleep, cognitive control, and depressive symptoms in young adults. Sleep Med. Jan 2014;15(1):144-149. [doi: 10.1016/j.sleep.2013.10. 006] [Medline: 24332565]
100. Ahmed A, Ramesh J, Ganguly S, Aburukba R, Sagahyroon A, Aloul F. Investigating the feasibility of assessing depression severity and valence-arousal with wearable sensors using discrete wavelet transforms and machine learning. Information. 2022;13(9):406. [doi: 10.3390/info13090406]
101. Choi J, Lee S, Kim S, Kim D, Kim H. Depressed mood prediction of elderly people with a wearable band. Sensors (Basel). 2022;22(11):4174. [doi: 10.3390/s22114174]
102. Mahendran N, Vincent DR, Srinivasan K, et al. Sensor-assisted weighted average ensemble model for detecting major depressive disorder. Sensors (Basel). Nov 6, 2019;19(22):4822. [doi: 10.3390/s19224822] [Medline: 31698678]
103. Xu X, Chikersal P, Doryab A, et al. Leveraging routine behavior and contextually-filtered features for depression detection among college students. Proc ACM Interact Mob Wearable Ubiquitous Technol. Sep 9, 2019;3(3):1-33. [doi: 10.1145/3351274]
104. Lu J, Shang C, Yue C, et al. Joint modeling of heterogeneous sensing data for depression assessment via multi-task learning. Proc ACM Interact Mob Wearable Ubiquitous Technol. Mar 26, 2018;2(1):1-21. [doi: 10.1145/3191753]
105. Farhan AA, Yue C, Morillo R, et al. Behavior vs. introspection: refining prediction of clinical depression via smartphone sensing data. Presented at: 2016 IEEE Wireless Health (WH); Oct 25-27, 2026; Bethesda, MD. [doi: 10.1109/WH.2016. 7764553]
106. Karimi S, Nateghi M, Cestero GI, et al. Prescreening depression using wearable electrocardiogram and photoplethysmogram data from a psycholinguistic experiment. Physiol Meas. Aug 2, 2025;46(8):085004. [doi: 10.1088/ 1361-6579/adf6fe] [Medline: 40752509]
107. Jacobson NC, Chung YJ. Passive sensing of prediction of moment-to-moment depressed mood among undergraduates with clinical levels of depression sample using smartphones. Sensors (Basel). Jun 24, 2020;20(12):3572. [doi: 10.3390/ s20123572] [Medline: 32599801]
108. Wang R, Wang W, Dasilva A, et al. Tracking depression dynamics in college students using mobile phone and wearable sensing. Proc ACM Interact Mob Wearable Ubiquitous Technol. Mar 2018;2(1):1-26. [doi: 10.1145/3191775] [Medline: 39449996]
109. Di Matteo D, Fotinos K, Lokuge S, et al. The relationship between smartphone-recorded environmental audio and symptomatology of anxiety and depression: exploratory study. JMIR Form Res. Aug 13, 2020;4(8):e18751. [doi: 10. 2196/18751] [Medline: 32788153]
110. Zhou Y, Yao X, Han W, Wang Y, Li Z, Li Y. Distinguishing apathy and depression in older adults with mild cognitive impairment using text, audio, and video based on multiclass classification and shapely additive explanations. Int J Geriat Psychiatry. Nov 2022;37(11):2345-2356. [doi: 10.1002/gps.5827]
111. Williamson JR, Young D, Nierenberg AA, Niemi J, Helfer BS, Quatieri TF. Tracking depression severity from audio and video based on speech articulatory coordination. Comput Speech Lang. May 2019;55:40-56. [doi: 10.1016/j.csl. 2018.08.004] [Medline: 32773961]
112. Shah RV, Grennan G, Zafar-Khan M, et al. Personalized machine learning of depressed mood using wearables. Transl Psychiatry. Jun 9, 2021;11(1):338. [doi: 10.1038/s41398-021-01445-0] [Medline: 34103481]
113. Opoku Asare K, Moshe I, Terhorst Y, et al. Mood ratings and digital biomarkers from smartphone and wearable data differentiates and predicts depression status: a longitudinal data analysis. Pervasive Mob Comput. Jul 2022;83:101621. [doi: 10.1016/j.pmcj.2022.101621]
114. Narziev N, Goh H, Toshnazarov K, Lee SA, Chung KM, Noh Y. STDD: short-term depression detection with passive sensing. Sensors (Basel). Mar 4, 2020;20(5):1396. [doi: 10.3390/s20051396] [Medline: 32143358]
115. Kim H, Lee S, Lee S, Hong S, Kang H, Kim N. Depression prediction by using ecological momentary assessment, Actiwatch data, and machine learning: observational study on older adults living alone. JMIR Mhealth Uhealth. Oct 16, 2019;7(10):e14149. [doi: 10.2196/14149] [Medline: 31621642]
116. Kumagai N, Tajika A, Hasegawa A, et al. Predicting recurrence of depression using lifelog data: an explanatory feasibility study with a panel VAR approach. BMC Psychiatry. Dec 11, 2019;19(1):391. [doi: 10.1186/s12888-019- 2382-2] [Medline: 31829206]
117. Funkhouser CJ, Weiner LS, Crowley RN, et al. Early changes in passively sensed homestay predict depression symptom improvement during digital behavioral activation. Behav Res Ther. Oct 2025;193:104815. [doi: 10.1016/j.brat.2025. 104815] [Medline: 40614686]
118. Di Matteo D, Fotinos K, Lokuge S, et al. Automated screening for social anxiety, generalized anxiety, and depression from objective smartphone-collected data: cross-sectional study. J Med Internet Res. Aug 13, 2021;23(8):e28918. [doi: 10.2196/28918] [Medline: 34397386]
119. Sverdlov O, Curcic J, Hannesdottir K, et al. A study of novel exploratory tools, digital technologies, and central nervous system biomarkers to characterize unipolar depression. Front Psychiatry. 2021;12:640741. [doi: 10.3389/fpsyt.2021. 640741] [Medline: 34025472]
120. Ben-Zeev D, Scherer EA, Wang R, Xie H, Campbell AT. Next-generation psychiatric assessment: using smartphone sensors to monitor behavior and mental health. Psychiatr Rehabil J. Sep 2015;38(3):218-226. [doi: 10.1037/prj0000130] [Medline: 25844912]
121. Minaeva O, Riese H, Lamers F, Antypa N, Wichers M, Booij SH. Screening for depression in daily life: development and external validation of a prediction model based on actigraphy and experience sampling method. J Med Internet Res. Dec 1, 2020;22(12):e22634. [doi: 10.2196/22634] [Medline: 33258783]
122. Dai R, Kannampallil T, Zhang J, Lv N, Ma J, Lu C. Multi-task learning for randomized controlled trials: a case study on predicting depression with wearable data. Proc ACM Interact Mob Wearable Ubiquitous Technol. 2022;6(2):1-23. [doi: 10.1145/3534591]
123. Tazawa Y, Liang KC, Yoshimura M, et al. Evaluating depression with multimodal wristband-type wearable device: screening and assessing patient severity utilizing machine-learning. Heliyon. Feb 2020;6(2):e03274. [doi: 10.1016/j. heliyon.2020.e03274] [Medline: 32055728]
124. Thati RP, Dhadwal AS, Kumar P, Sainaba P. Multimodal depression detection: using fusion strategies with smart phone usage and audio-visual behavior. Int J Artif Intell Tools. Mar 2023;32(2):2340008. [doi: 10.1142/S0218213023400080]
125. Jiang Z, Seyedi S, Griner E, et al. Multimodal mental health digital biomarker analysis from remote interviews using facial, vocal, linguistic, and cardiovascular patterns. IEEE J Biomed Health Inform. Mar 2024;28(3):1680-1691. [doi: 10.1109/JBHI.2024.3352075] [Medline: 38198249]
126. Moshe I, Terhorst Y, Opoku Asare K, et al. Predicting symptoms of depression and anxiety using smartphone and wearable data. Front Psychiatry. 2021;12:625247. [doi: 10.3389/fpsyt.2021.625247] [Medline: 33584388]
127. Wang R, Chen F, Chen Z, et al. StudentLife: assessing mental health, academic performance and behavioral trends of college students using smartphones. Presented at: UbiComp ’14: Proceedings of the 2014 ACM International Joint Conference on Pervasive and Ubiquitous Computing; Sep 13-17, 2014; Seattle, Washington. [doi: 10.1145/2632048. 2632054]
128. Song S, Seo Y, Hwang S, Kim HY, Kim J. Digital phenotyping of geriatric depression using a community-based digital mental health monitoring platform for socially vulnerable older adults and their community caregivers: 6-week living lab single-arm pilot study. JMIR Mhealth Uhealth. Jun 17, 2024;12:e55842. [doi: 10.2196/55842] [Medline: 38885033]
129. Bai R, Xiao L, Guo Y, et al. Tracking and monitoring mood stability of patients with major depressive disorder by machine learning models using passive digital data: prospective naturalistic multicenter study. JMIR Mhealth Uhealth. Mar 8, 2021;9(3):e24365. [doi: 10.2196/24365] [Medline: 33683207]
130. Borelli JL, Wang Y, Li FH, et al. Detection of depressive symptoms in college students using multimodal passive sensing data and light gradient boosting machine: longitudinal pilot study. JMIR Form Res. Jun 3, 2025;9:e67964. [doi: 10.2196/67964] [Medline: 40460426]
131. Chen J, Chan NY, Li CT, et al. Multimodal digital assessment of depression with actigraphy and app in Hong Kong Chinese. Transl Psychiatry. Mar 18, 2024;14(1):150. [doi: 10.1038/s41398-024-02873-4] [Medline: 38499546]
132. Aledavood T, Luong N, Baryshnikov I, et al. Multimodal digital phenotyping study in patients with major depressive episodes and healthy controls (mobile monitoring of mood): observational longitudinal study. JMIR Ment Health. Feb 21, 2025;12:e63622. [doi: 10.2196/63622] [Medline: 39984168]
