# Supplementary material for: The Role of Digital Biomarkers in Physiological Signal-Based Depression Assessment: Systematic Review and Meta-Analysis
Source: J Med Internet Res. 2026 Apr 2;28:e76432. doi: 10.2196/76432 (PMC13046098; doi:10.2196/76432)
Supplement: Multimedia Appendix 3 [file jmir-v28-e76432-s003.docx]

**Multimedia Appendix 3. Participants and analytical methods in the studies.**

| **Study Type** | **Description** | **Number of Studies** |
| --- | --- | --- |
| 1 | Comparison of depression vs. no depression groups (e.g., using t-tests) | 12 |
| 2 | Reporting correlations between depression indicators and biomarkers in the general adult population | 10 |
| 3 | Reporting correlations between depression indicators and biomarkers in patients with depression | 9 |
| 4 | Reporting the explanatory power of depression biomarkers (independent variable) on depression indicators (dependent variable) (e.g., logistic regression) | 42 |
| 5 | Reporting the diagnostic accuracy of depression biomarkers (sensitivity, specificity, accuracy, recall, Root Mean Squared Error (RMSE), Area under the curve (AUC), Receiver Operating Characteristics (ROC) Curve) | 59 |
