# Supplementary material for: The Role of Digital Biomarkers in Physiological Signal-Based Depression Assessment: Systematic Review and Meta-Analysis
Source: J Med Internet Res. 2026 Apr 2;28:e76432. doi: 10.2196/76432 (PMC13046098; doi:10.2196/76432)
Supplement: Multimedia Appendix 4 [file jmir-v28-e76432-s004.docx]

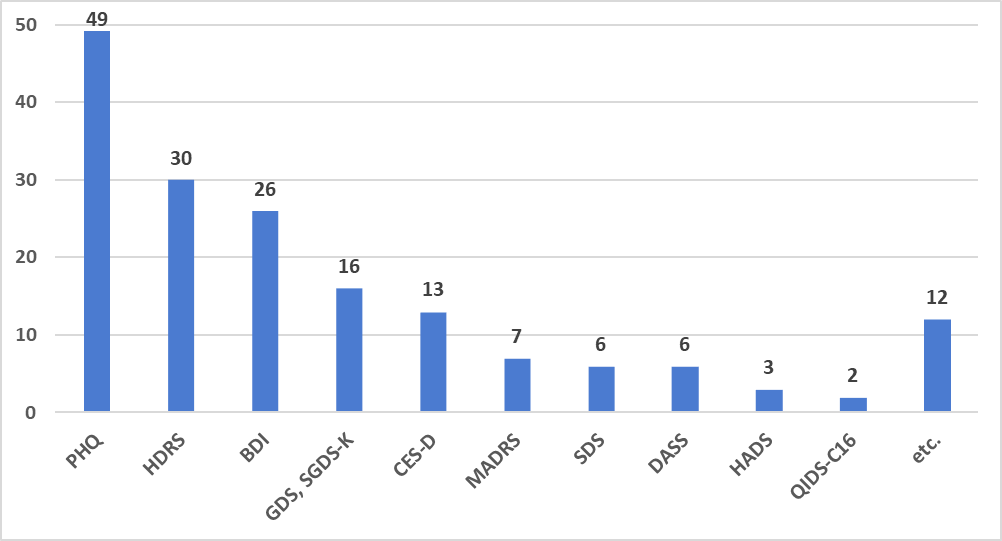
PHQ, Patient Health Questionnaire; HDRS, Hamilton Depression Rating Scale; BDI, Beck Depression Inventory; GDS, Geriatric Depression Scale; SGDS-K, shortened version of the Geriatric Depression Scale in Korean; CES-D, Center for Epidemiologic Studies Depression Scale; MADRS, Montgomery-Åsberg Depression Rating Scale; SDS, Zung Self-Rating Depression Scale; DASS, Depression Anxiety Stress Scale; HADS, Hospital Anxiety and Depression Scale; QIDS-C16, Quick Inventory of Depressive Symptomatology - Clinician Rating 16-item

**Multimedia Appendix 4. Depression measurement tools included in the studies.**
