# Supplementary figures and images for: The Role of Digital Biomarkers in Physiological Signal-Based Depression Assessment: Systematic Review and Meta-Analysis
Source: J Med Internet Res. 2026 Apr 2;28:e76432. doi: 10.2196/76432 (PMC13046098; doi:10.2196/76432)

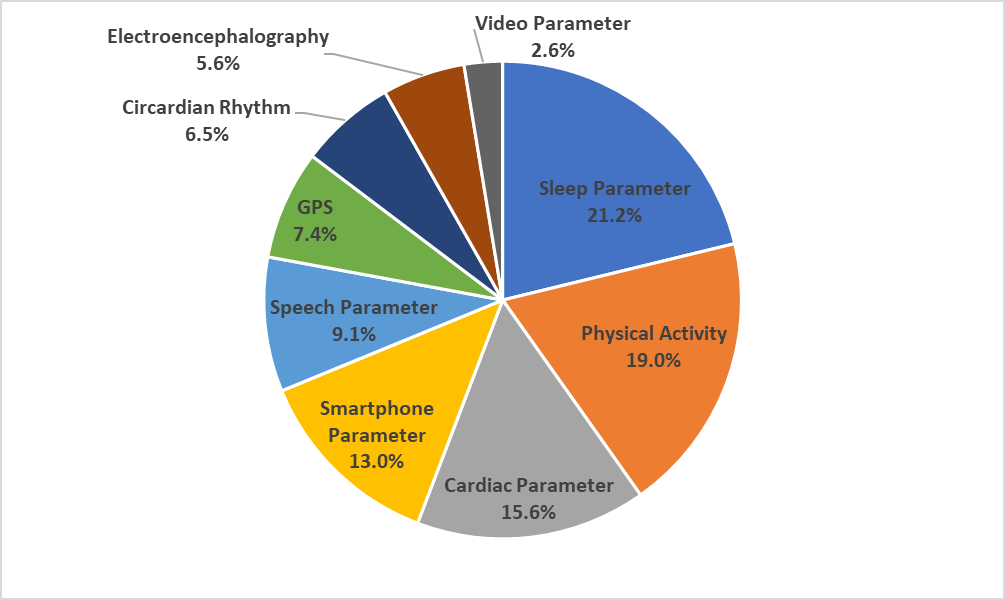


**Multimedia Appendix 5. Digital biomarkers included in the studies**

Supplement: Multimedia Appendix 5 [file jmir-v28-e76432-s005.docx]
