# Supplementary material for: The Role of Digital Biomarkers in Physiological Signal-Based Depression Assessment: Systematic Review and Meta-Analysis
Source: J Med Internet Res. 2026 Apr 2;28:e76432. doi: 10.2196/76432 (PMC13046098; doi:10.2196/76432)
Supplement: Multimedia Appendix 6 [file jmir-v28-e76432-s006.docx]

**Multimedia Appendix 6. Digital biomarkers not included in the meta-analysis.**

| **Feature** | **Finding in Individual Studies** | **References** |
| --- | --- | --- |
| Sleep parameters | | |
| Sleep fragmentation | Frequent sleep fragmentation has been significantly associated with depression, with patients experiencing depression exhibiting more nighttime awakenings than controls. Studies also found that the number of awakenings was a key predictor of depression [1,2] and treatment outcomes [3]. | [1-3] |
| Rapid eye movement sleep (REM sleep) | Most studies on REM sleep in depression have been based on single hospital measurements with small sample sizes, making conclusions difficult. A follow-up study found that REM sleep was significantly reduced in patients treated for depression, especially in the first third of the night [4]. While some studies suggest REM sleep as a predictor of depression [3], others report no group differences [5,6] or no explanatory value of REM duration [7,8]. In older adults, REM sleep has sometimes been shown to decrease, possibly reflecting accelerated aging processes [9]. Overall, evidence for REM sleep time or frequency as a digital biomarker of depression remains inconclusive. | [3-9] |
| REM sleep latency | Some studies reported that REM latency significantly increased in depressed patients receiving pharmacological treatment compared with untreated patients [4], while others found no difference between depressed and control groups [5,9]. In older adults with depression, REM latency has been reported as either comparable to controls or significantly longer [6]. However, analyses using longitudinal models suggest that sleep onset latency may serve as a more reliable indicator of depression than REM latency or REM proportion [7]. Since most studies measured REM latency only once in hospital settings with small sample sizes, evidence supporting its role as a digital biomarker of depression remains inconclusive. | [4-7,9] |
| Non-rapid eye movement sleep (NREM sleep) | Apart from one study using a linear mixed model that reported a significant association [7], no studies have examined the link between NREM sleep and depression [4,6]. Evidence supporting NREM sleep as a digital biomarker of depression remains unclear. | [4,6,7] |
| Slow wave sleep (SWS) | No studies have reported an association between SWS and depression [4-6,9]. | [4-6,9] |
| Sleep onset | A study involving hospitalized patients reported a weak negative correlation between sleep onset time and depression (r = -0.381), showing a delay of about six minutes before discharge compared to treatment initiation [10]. Other studies found no significant associations or did not provide detailed results [6,7,11,12]. | [6,7,10-12] |
| Sleep midpoint | Three studies, one in working adults [13] and another in middle-aged women [14], and a clinical sample of Chinese patients with MDD [15], reported that higher depression scores were associated with a later sleep midpoint. Other studies found no significant associations [6,10,16]. | [6,10,13-16] |
| Sleep offset | Studies on working adults and patients with depression have shown that higher depression scores or depressive status are associated with later sleep offset [13]. In older adults, actigraphy indicated significantly later sleep offset in depressed patients, whereas polysomnography showed no difference [6], suggesting variation by measurement method. Overall, sleep offset may be a potential digital biomarker for depression, but further studies are needed. | [6,10,13,14] |
| Physical activity | | |
| Light physical activity (LPA) | In adults, greater time spent in LPA was associated with fewer depressive symptoms at follow-up [17,18]. However, other studies reported no significant changes in LPA despite increased depression during the COVID-19 pandemic [19], no significant effect of LPA after r-TMS treatment [3], and low variable importance in college populations [20]. Consistently, a study on socially vulnerable older adults found that LPA did not emerge as a primary daily predictor of symptom fluctuations when compared to more robust metrics such as sleep efficiency [21]. Furthermore, recent evidence from a college student sample indicates that the predictive utility of LPA is significantly enhanced only when integrated with other passive sensing modalities, such as heart rate variability and sleep, rather than being analyzed in isolation [22]. Overall, findings are inconsistent, suggesting that LPA may serve only as a supplementary digital biomarker alongside measures, such as MVPA or step count, or multimodal sensing features [21,22]. | [3,17-22] |
| Energy expenditure | A study found that depressed patients had significantly lower energy expenditure than controls [23]. A longitudinal study observed changes in energy expenditure with symptom improvement [24], while another reported no difference between depressed and control groups [25]. Overall, findings on energy expenditure in depression are inconsistent. | [23-25] |
| Cardiac parameters | | |
| Root mean square of the successive differences (RMSSD) | A study comparing first-episode depressed patients with controls reported a significant association with depression [26]. Some studies found lower RMSSD in depressed patients, but without statistical significance [27-30], and others showed no correlation between depression scores and RMSSD [31-34]. Recent digital phenotyping research has further clarified these inconsistencies; for instance, while RMSSD was identified as a key feature in machine learning models for depression prescreening [35], its predictive power often depends on being part of a multimodal framework including sleep and activity data [22]. In older populations, daily fluctuations in RMSSD have shown limited direct association with depressive symptom severity compared to sleep-related biomarkers [21]. Consequently, further validation of RMSSD as a standalone biomarker is required, particularly regarding its integration into comprehensive digital assessment models [21,22,35]. | [21,22,26-35] |
| Proportion of normal-to-normal intervals (pNN) | Three studies reported no significant difference in pNN between depressed and non-depressed patients [28,30,31], though a slightly lower trend was observed in the depressed group in one study [29]. A diagnostic algorithm study identified pNN as an optimal feature [33,36]. More recently, pNN50 has been identified as an important physiological feature for depression prescreening in wearable-based machine learning models, suggesting its potential when analyzed alongside other temporal and spectral HRV parameters [35]. However, despite these technical applications, its clinical value as a standalone biomarker remains unclear. | [28-31,33,35,36] |
| Mean RR interval (mean RRI) | Two studies reported that mean RRI was significantly lower with greater depression severity [26,31], while two others found no significant correlation [28,37]. Mean RRI tends to be shorter in depression, suggesting autonomic imbalance, but the limited number of studies indicates that further studies are needed. | [26,28,31,37] |
| Standard deviation of heart rate (SD of HR) | In recurrent depression, lower daily heart rate standard deviation (β = -0.30) was associated with higher PHQ-8 scores [38], indicating greater severity with reduced variability. In contrast, a study on working adults found a positive correlation between nighttime HRV and depression [13], while another showed it was not a significant predictor in final models [39]. Overall, evidence on heart rate standard deviation and depression is mixed and inconclusive. | [13,38,39] |
| High frequency (HF) | Several studies have reported lower HF in depressed patients compared to controls [27,31,40]. In end-stage renal disease, this difference was significant in men but not in women [37], and a longitudinal analysis showed that HF predicted depressive symptoms seven years later [41]. Other studies found only a nonsignificant trend toward lower HF in severe depression [29]. In one correlation study, HF was the only HRV variable showing a significant negative association with depression scores, suggesting it may be the most useful among HRV measures [28]. Consistent findings also showed higher HF in controls than in depressed patients before and after mental tasks [42], though some multivariate models reported nonsignificant results [26,33]. Recent digital phenotyping using wearable devices similarly highlights reduced HF power as a discriminative feature for depression prescreening [35], though its daily predictive utility may be less prominent in older populations compared to objective sleep metrics [21]. Overall, HF tends to be reduced with greater depression severity and may serve as a potential digital biomarker. | [21,26-29,31,33,35,37,40-42] |
| Low frequency (LF) | Some studies reported lower LF in depressed patients [27,31,37], and a longitudinal study showed that LF predicted depressive symptoms seven years later [41]. In contrast, other studies found higher LF in depression or no significant correlation with depression scores [26,28,33]. More recently, wearable-based screening research has utilized LF as a relevant feature within multivariate machine learning models, although its individual importance was often rated lower than other spectral or time-domain metrics [35]. Consequently, its specific role and reliability as a standalone biomarker of depression remain unclear. | [26-28,31,33,35,37,41] |
| LF/HF ratio | Some studies reported significantly higher LF/HF in depressed patients compared to controls [26,31,42], while others found no correlation with depression scores after adjustment [27-29,33]. Recent evidence from wearable ECG studies supports the inclusion of the LF/HF ratio as a contributing feature in automated depression screening models [35], although its daily predictive strength may be secondary to sleep-related digital biomarkers in specific populations, such as socially vulnerable older adults [21]. Overall, the LF/HF ratio may show an increasing trend with depression, but it requires further validation as a reliable standalone biomarker. | [21,26-29,31,33,35,42] |
| Very low frequency (VLF) | Studies have identified VLF as an important feature in depression diagnostic algorithms [33], with lower values observed in men with coronary artery disease [29] and end-stage renal disease [37]. Longitudinal analysis also showed that VLF predicted depressive symptoms [41], though another study found no correlation after adjusting for other variables [27]. Overall, VLF tends to be lower in depressed patients and may serve as a potential digital biomarker, but further studies are needed. | [27,29,33,37,41] |
| Ultra-low frequency (ULF) | ULF was reported to decrease with greater depression severity [29] and predict depressive symptoms [41], though another study found no significant difference between depressed and control groups [37]. Overall, ULF tends to be lower in depression, but the limited number of studies calls for further studies. | [29,37,41] |
| Total power (TP) | Among the four studies examining TP and depression, one reported a significant effect in multivariate models [26], while the other three found no association with depression severity or differences between groups [27-29]. | [26-29] |
| Smartphone parameters | | |
| Phone usage frequency | Some studies suggest that features, such as physical activity or sleep, are more useful than phone usage frequency [43-45], with some reporting no correlation between usage frequency and depression [34,46]. A positive association was observed only in a study of young adults [47]. Other studies highlight the importance of regularity indices (e.g., screen or internet use) and communication patterns over simple usage counts or duration [48]. Notably, recent longitudinal monitoring in patients with major depressive episodes revealed that while phone usage patterns (e.g., number of unlocks or session duration) were part of the digital phenotype, they often exhibited high individual variability [49]. Furthermore, in college populations, the importance of phone usage features was found to be lower than that of sleep and heart rate variability when detecting depressive symptoms, highlighting the need for multimodal integration [22]. | [22,34,43-49] |
| Phone usage duration | Studies in young adults and college students reported a positive correlation between depression and phone use time [11,47,50], while other studies found shorter use in depressed groups or no association at all [23,34]. Some studies also suggest that features, like physical activity or sleep, are more informative than phone use duration [12,50]. Consistent with this, recent multimodal sensing in college students demonstrated that the feature importance of phone usage duration in detecting depressive symptoms was substantially lower than that of heart rate variability and sleep metrics [22]. Overall, the relationship between phone use time and depression is inconsistent, limited by low individual predictive power compared to physiological indicators, and may vary significantly by age group [12,22]. | [11,12,22,23,34,47,50] |
| Phone calls | Most studies used call data alongside other features rather than as a standalone biomarker, with physical activity and sleep shown to be more useful [12,43,44]. While some studies noted reduced phone communication [51] or included calls in optimal models [52], younger adults tend to rely more on text-based communication through apps or social media. Recent longitudinal sensing reinforces this, showing that the feature importance of phone call logs in detecting depressive symptoms is significantly lower than that of physiological indicators like heart rate variability HRV [22]. Furthermore, communication patterns via calls often exhibit high individual variability among patients with major depressive episodes, making it difficult to establish a consistent group-level biomarker [49]. Overall, phone calls have limited potential as a standalone digital biomarker for depression but may contribute to comprehensive screening within multimodal frameworks [22,49]. | [12,22,43,44,49,51,52] |
| Light exposure | Some studies reported that light exposure predicted depression relapse in patients under maintenance treatment [53] and was a significant factor in logistic regression among older adults living alone [54], while others found it was not an important variable [24,44,45]. Light exposure may have potential relevance for depression relapse or late-life depression, but further studies are needed. | [24,44,45,53,54] |
| Number of Bluetooth-connected devices | A study found a negative correlation between the number of Bluetooth connections and PHQ scores [55], while others suggested it could be included as a feature in optimal models without direct analysis [52,50]. Taken together, the number of Bluetooth connections may serve as an indicator of social activity or environmental engagement, but further studies are needed. | [50,52,55] |
| Typing pattern | A machine learning model using keystroke timing sequences and typing metadata collected from natural smartphone typing achieved optimal performance of 86% and 89% [47,56]. Typing patterns may serve as a meaningful digital biomarker for depression, but further studies are needed. | [47,56] |
| Speech parameters | | |
| Speech rate | Some studies found slower speech in more severe depression [57,58], and models including speech rate and duration outperformed acoustic features [59]. Recent evidence further confirms that reduced speech rate is a significant indicator of MDD, often correlating with both depressive symptoms and objective executive dysfunction [60]. Additionally, multimodal models using remote interview data have shown that speech rate remains a robust vocal feature for distinguishing between depressed and healthy individuals [61]. Speech rate shows potential as a digital biomarker for depression, but further studies are needed. | [57-61] |
| Speech duration | Four studies reported reduced speaking time in more severe depression [11, 55,59,62], and one study found it to be the strongest predictor among various smartphone data features [45]. Recent automated assessments have also demonstrated that depressed patients exhibit significantly lower mobile or active speaking time compared to controls [15,61]. Findings consistently suggest less conversation in depressed patients, indicating potential as a digital biomarker. | [11,15,45,55,59,61,62] |
| Pause time | While associations between depression and speech-pause duration have occasionally been non-significant [63], the majority of studies [57,58]. Recent objective speech analysis has reinforced that increased time spent pausing is a key characteristic of depressive speech, effectively capturing the psychomotor retardation associated with the disorder [60]. When integrated with facial and linguistic patterns, pause-related features contribute to high-accuracy multimodal detection models [61]. However, only a few studies have highlighted the need for further investigation. | [57,58,60,61,63] |
| Mel-frequency cepstral coefficients (MFCCs) | Most studies found MFCC to significantly explain depression severity [60,63-65], though one reported better performance with text feature models than with acoustic features, including MFCC [59]. Recent multimodal analysis further supports MFCCs as a key vocal biomarker, demonstrating their effectiveness in distinguishing depressed individuals when integrated with facial and cardiovascular patterns [61]. While MFCCs show strong potential as digital biomarkers, they require further validation across diverse clinical settings. | [59-61,63-65] |
| Fundamental frequency (F0) | Among five studies on F0 and depression, three reported significant group differences [64] or identified F0 as an important predictor [63,65], while others found no association [60,66]. However, recent multimodal analysis suggests that while F0 remains a key vocal feature, its effectiveness in depression screening is significantly enhanced when integrated with facial and cardiovascular patterns, helping to mitigate its individual inconsistency [61]. Given these inconsistent results, further studies are needed. | [60,61,63-66] |
| Jitter | A significant difference was observed between the depressed group (1.11 ± 1.43) and the control group (0.27 ± 0.22) (p < .001) [66], while the other two studies did not identify it as a significant variable [59,63]. However, recent multimodal research suggests that jitter can still provide discriminative value when combined with facial and cardiovascular features, despite its individual inconsistency [61]. Results are inconsistent across studies, indicating the need for further studies. | [59,61,63,66] |
| Shimmer | A study reported a significant difference between the depressed (8.57 ± 4.25) and control group (4.94 ± 3.16) (p < .001) [66], and served as an important predictor in older adults [63]. While one study found no significant association [59], recent evidence indicates that shimmer's predictive power is notably enhanced within multimodal frameworks, contributing to more robust detection than when used alone [61]. These inconsistent findings across individual studies highlight the necessity for further investigation, particularly within integrated sensing systems. | [59,61,63,66] |
| GPS parameters | | |
| Total distance | Some studies reported a negative correlation between depression and total distance [20,23,55,62,67,68], with recent evidence further supporting mobility patterns—such as reduced location variance and entropy—as robust indicators [49]. While the remaining three studies, although not statistically significant, also showed a tendency for total distance to decrease with increasing depression severity [34,47,69]. Thus, reduced distance remain promising digital biomarker, particularly when integrated into multimodal detection models [22]. | [22,23,34,47,49,55,62,67-69,] |
| Location variance | Lower location variance has been consistently associated with higher depression severity [11,23,34,47,69], with recent evidence further validating it as a robust digital phenotyping marker for MDD patients [49]. Specifically, in one study, location variance was included as a feature in the optimal ensemble model [52], further supporting the link between reduced mobility patterns and depression. While one study noted inconsistencies depending on smartphone types [20], location variance remains a key feature in high-accuracy multimodal models for detecting depressive symptoms [22]. | [11,20,22,23,34,47,49,52,69] |
| Time spent at home | Most studies reported a significant positive correlation between depression severity and time spent at home [11,20,23,47,69], with recent evidence further identifying early changes in "homestay" as a critical predictor of symptom improvement during digital interventions [16]. While its individual predictive power can vary, time spent at home remains a robust digital biomarker, especially when integrated with other passive sensing data in multimodal detection models [22,49]. | [11,16,20,22,23,47,49,69] |
| Location entropy | Most studies using GPS-measured location entropy reported a negative correlation between location entropy and depression scores [20,23,69], with recent longitudinal evidence further confirming that lower entropy is a significant indicator of major depressive episodes compared to healthy controls [49]. Although not statistically significant, a study of 28 adults also found lower location entropy in individuals with depression [47]. However, a study of 83 college students found no significant association between depression severity and location entropy [11]. | [11,20,23,47,49,69] |
| Normalized location entropy | Normalized location entropy, which accounts for the total number of visited places, has shown a more consistent negative correlation with depression severity [20,23,47,69]. Recent research supports this trend, indicating that reduced movement diversity—as captured by normalized entropy—is a core mobility pattern in depressed individuals [49]. Furthermore, integrating this normalized metric into multimodal sensing frameworks has been shown to significantly enhance the accuracy of detecting depressive symptoms compared to using unimodal features alone [22]. This consistent trend is further supported by the fact that even the one nonsignificant study showed a decreasing trend with greater depression severity [34]. | [20,22,23,34,47,49,69] |
| Number of locations visited | Among five studies examining the number of places visited using GPS, four reported a negative correlation between depression and the number of places visited [11,20,23,45], a finding further validated by recent longitudinal data showing that MDD patients visit significantly fewer unique locations than healthy controls [49]. While one study found no correlation with depression scores [47], the number of visited locations has been identified as a key contributing feature in high-accuracy multimodal models, where it enhances the detection of depressive symptoms [22]. | [11,20,22,23,45,47,49] |
| Time spent in moving | While findings regarding time spent moving have been inconsistent—with one study reporting a negative correlation only among Android users [20] and others finding no significant association [34,47,69]—recent longitudinal data suggests that early changes in physical activity duration can serve as a critical predictor of symptom improvement [16]. Furthermore, although individual significance may be limited, time-related mobility features have been identified as essential components in high-performance multimodal frameworks, contributing to more robust depression detection [22]. | [16,20,22,34,47,69] |
| Average moving speed | Average movement speed has been identified as a key feature in depression prediction models [70], although its individual correlation often varies by device type [20]. Recent evidence further shows that MDD patients generally exhibit significantly lower movement speeds compared to healthy controls [49]. Furthermore, while its standalone predictive power may be limited, average speed remains a crucial component in high-accuracy multimodal frameworks, contributing to more robust detection of depressive symptoms [22]. | [20,22,49,70] |
| Circadian rhythm | | |
| Interdaily stability (IS) | In studies of 1,734 and 1,552 adults, IS was identified as an important feature in depression classification models [71,72]. Another study reported a negative correlation between depression scores and IS, indicating less regular daily activity with higher depression severity [13]. However, no significant differences were found between depressed and control groups [15,25,73,74], and IS values did not differ between treatment responders and non-responders [75], suggesting limited evidence for IS as an independent digital biomarker of depression. | [13,15,25,71-75] |
| Intradaily variability (IV) | Generally, IV is unrelated to depression [15,25,71,73-75]. Although one study of 1,734 adults associated higher depression scores with increased IV—indicating more frequent activity fluctuations and less stable rest-activity transitions [72]—evidence for IV as an independent digital biomarker remains limited. | [15,25,71-75] |
| Midline estimating statistic of rhythm (MESOR) | MESOR was lower in individuals with greater depression severity in two studies [12,76] and was identified as an important predictor in another study [71]. Older men in the lowest MESOR quartile had a 2.04-fold higher risk of clinically significant depressive symptoms (95% CI: 1.36–3.04) [77]. Consistently, a recent multimodal study found that MDD subjects exhibit significantly lower MESOR than healthy controls (p = .003) [15]. While some studies found no changes before and after treatment [75] or no predictive value [78]. Overall, lower MESOR may reflect reduced energy in depression, but evidence as an independent digital biomarker remains limited. | [12,15,71,75-78] |
| Amplitude | The association between amplitude and depression has shown varying results across different populations. In older men, those in the lowest amplitude quartile had a 1.37-fold higher risk of moderate depressive symptoms (95% CI: 1.07–1.75) [77], and among 3,020 women with osteoporosis, amplitude tended to decrease with greater depression severity [76]. However, other studies found no pre- and post-treatment differences [75] or reported limited predictive value [71,78,12,15]. Overall, depressed individuals show reduced differences between peak and trough activity levels, but evidence for amplitude as an independent digital biomarker of depression remains limited. | [12,15,71,75-78] |
| Acrophase, peak | While most studies have found no significant correlation between acrophase and depression [12,13,75-77] or reported only limited predictive value [78], a recent study observed a significantly later acrophase in MDD patients, suggesting a potential delay in the circadian phase [15]. However, given the inconsistency with earlier findings, the evidence for acrophase as a reliable independent digital biomarker remains inconclusive, and further validation is required to clarify these mixed results. | [12,13,15,75-78] |
| Relative amplitude (RA) | Four studies examined the relationship between RA and depression. In two studies, depressed individuals showed lower RA than controls, indicating reduced daily activity variation and overall activity levels, though differences were not significant [25,73]. Two other studies also found no association between RA and depression [71,75]. Evidence for RA as a digital biomarker of depression remains limited. | [25,71,73,75] |
| Pseudo F-statistic (F-pseudo) | Among four studies, one involving 3,020 women with osteoporosis reported lower values in those with depression [76], and another with 2,124 men found that lower F-pseudo values were associated with more severe depression [77], indicating weaker or irregular circadian rhythms in depressed individuals. Two studies found no significant associations [13,71]. Overall, F-pseudo tended to decrease with depression, but further studies are needed due to the limited number of studies. | [13,71,76,77] |
| Most active 10-h period (M10) | Three studies examined M10 and depression, and none found significant differences [25,71,75]. There is no evidence supporting M10 as a digital biomarker of depression. | [25,71,75] |
| Least active 5-h period (L5) | Three studies examined L5 and depression, and none reported a significant association [25,71,75]. | [25,71,75] |

**References**

1. Smith P, Kandakatla A, Frankel CW, et al. Sleep quality, depressive symptoms, and transplant outcomes: follow-up analyses from the ADAPT prospective pilot study. Gen Hosp Psychiatry. 2021;72:53-58. [doi: 10.1016/j.genhosppsych. 2021.06.011] [Medline: 34298477]
2. Coffield TG, Tryon WW. Construct validation of actigraphic sleep measures in hospitalized depressed patients. Behav Sleep Med. 2004;2(1):24-40. [doi: 10.1207/s15402010bsm0201_3] [Medline: 15600222]
3. Griffiths C, da Silva KM, Leathlean C, Jiang H, Ang CS, Searle R. Investigation of physical activity, sleep, and mental health recovery in treatment resistant depression (TRD) patients receiving repetitive transcranial magnetic stimulation (rTMS) treatment. J Affect Disord Rep. Apr 2022;8:100337. [doi: 10.1016/j.jadr.2022.100337] [Medline: 35619990]
4. Luthringer R, Toussaint M, Schaltenbrand N, et al. A double-blind, placebo-controlled evaluation of the effects of orally administered venlafaxine on sleep in inpatients with major depression. Psychopharmacol Bull. 1996;32(4):637-646. [Medline: 8993085]
5. Landolt HP, Gillin JC. Similar sleep EEG topography in middle-aged depressed patients and healthy controls. Sleep. Feb 2005;28(2):239-247. [doi: 10.1093/sleep/28.2.239] [Medline: 16171249]
6. Hoyos CM, Gordon C, Terpening Z, et al. Circadian rhythm and sleep alterations in older people with lifetime depression: a case-control study. BMC Psychiatry. Apr 29, 2020;20(1):192. [doi: 10.1186/s12888-020-02606-z] [Medline: 32349697]
7. Zhang Y, Folarin AA, Sun S, et al. Predicting depressive symptom severity through individuals’ nearby Bluetooth device count data collected by mobile phones: preliminary longitudinal study. JMIR Mhealth Uhealth. Jul 30, 2021;9(7):e29840. [doi: 10.2196/29840] [Medline: 34328441]
8. Gould CE, Karna R, Jordan J, et al. Subjective but not objective sleep is associated with subsyndromal anxiety and depression in community-dwelling older adults. Am J Geriatr Psychiatry. Jul 2018;26(7):806-811. [doi: 10.1016/j.jagp. 2018.03.010] [Medline: 29709510]
9. Lee HJ, Lee JS, Kim T, Yoon IY. Relationship between sleep disturbances and cognitive impairments in older adults with depression. Sleep Medicine and Psychophysiology. Jun 30, 2014;21(1):5-13. [doi: 10.14401/KASMED.2014.21.1. 5]
10. Raoux N, Benoit O, Dantchev N, et al. Circadian pattern of motor activity in major depressed patients undergoing antidepressant therapy: relationship between actigraphic measures and clinical course. Psychiatry Res. Apr 1994;52(1):85-98. [doi: 10.1016/0165-1781(94)90122-8] [Medline: 8047624]
11. Wang R, Wang W, Dasilva A, et al. Tracking depression dynamics in college students using mobile phone and wearable sensing. Proc ACM Interact Mob Wearable Ubiquitous Technol. Mar 2018;2(1):1-26. [doi: 10.1145/3191775] [Medline: 39449996]
12. Bai R, Xiao L, Guo Y, et al. Tracking and monitoring mood stability of patients with major depressive disorder by machine learning models using passive digital data: prospective naturalistic multicenter study. JMIR Mhealth Uhealth. Mar 8, 2021;9(3):e24365. [doi: 10.2196/24365] [Medline: 33683207]
13. Rykov Y, Thach TQ, Bojic I, Christopoulos G, Car J. Digital biomarkers for depression screening with wearable devices: cross-sectional study with machine learning modeling. JMIR Mhealth Uhealth. Oct 25, 2021;9(10):e24872. [doi: 10.2196/24872] [Medline: 34694233]
14. Bowman MA, Kline CE, Buysse DJ, et al. Longitudinal association between depressive symptoms and multidimensional sleep health: the SWAN Sleep Study. Ann Behav Med. Jun 28, 2021;55(7):641-652. [doi: 10.1093/abm/kaaa107] [Medline: 33410460]
15. Chen J, Chan NY, Li CT, et al. Multimodal digital assessment of depression with actigraphy and app in Hong Kong Chinese. Transl Psychiatry. Mar 18, 2024;14(1):150. [doi: 10.1038/s41398-024-02873-4] [Medline: 38499546]
16. Funkhouser CJ, Weiner LS, Crowley RN, et al. Early changes in passively sensed homestay predict depression symptom improvement during digital behavioral activation. Behav Res Ther. Oct 2025;193:104815. [doi: 10.1016/j.brat.2025. 104815] [Medline: 40614686]
17. Li YM, Konstabel K, Mõttus R, Lemola S. Temporal associations between objectively measured physical activity and depressive symptoms: an experience sampling study. Front Psychiatry. 2022;13:920580. [doi: 10.3389/fpsyt.2022. 920580] [Medline: 35923450]
18. Ku PW, Steptoe A, Liao Y, Sun WJ, Chen LJ. Prospective relationship between objectively measured light physical activity and depressive symptoms in later life. Int J Geriatr Psychiatry. Jan 2018;33(1):58-65. [doi: 10.1002/gps.4672] [Medline: 28181713]
19. Mishra R, Park C, York MK, et al. Decrease in mobility during the COVID-19 pandemic and its association with increase in depression among older adults: a longitudinal remote mobility monitoring using a wearable sensor. Sensors (Basel). Apr 29, 2021;21(9):3090. [doi: 10.3390/s21093090] [Medline: 33946664]
20. Lu J, Shang C, Yue C, et al. Joint modeling of heterogeneous sensing data for depression assessment via multi-task learning. Proc ACM Interact Mob Wearable Ubiquitous Technol. Mar 26, 2018;2(1):1-21. [doi: 10.1145/3191753]
21. Song S, Seo Y, Hwang S, Kim HY, Kim J. Digital phenotyping of geriatric depression using a community-based digital mental health monitoring platform for socially vulnerable older adults and their community caregivers: 6-week living lab single-arm pilot study. JMIR Mhealth Uhealth. Jun 17, 2024;12:e55842. [doi: 10.2196/55842] [Medline: 38885033]
22. Borelli JL, Wang Y, Li FH, et al. Detection of depressive symptoms in college students using multimodal passive sensing data and light gradient boosting machine: longitudinal pilot study. JMIR Form Res. Jun 3, 2025;9:e67964. [doi: 10.2196/67964] [Medline: 40460426]
23. Opoku Asare K, Moshe I, Terhorst Y, et al. Mood ratings and digital biomarkers from smartphone and wearable data differentiates and predicts depression status: a longitudinal data analysis. Pervasive Mob Comput. Jul 2022;83:101621. [doi: 10.1016/j.pmcj.2022.101621]
24. Tazawa Y, Liang KC, Yoshimura M, et al. Evaluating depression with multimodal wristband-type wearable device: screening and assessing patient severity utilizing machine-learning. Heliyon. Feb 2020;6(2):e03274. [doi: 10.1016/j. heliyon.2020.e03274] [Medline: 32055728]
25. Lorenz N, Spada J, Sander C, Riedel-Heller SG, Hegerl U. Circadian skin temperature rhythms, circadian activity rhythms and sleep in individuals with self-reported depressive symptoms. J Psychiatr Res. Oct 2019;117:38-44. [doi: 10. 1016/j.jpsychires.2019.06.022] [Medline: 31279242]
26. Liang CS, Lee JF, Chen CC, Chang YC. Reactive heart rate variability in male patients with first-episode major depressive disorder. Prog Neuropsychopharmacol Biol Psychiatry. Jan 2, 2015;56:52-57. [doi: 10.1016/j.pnpbp.2014.08. 004] [Medline: 25149628]
27. Lee MS, Seo YE, Mok YE, Lee SH. Heart rate variability after treatment for depression in North Korean defectors. Appl Psychophysiol Biofeedback. Mar 2021;46(1):11-18. [doi: 10.1007/s10484-020-09491-y] [Medline: 33074464]
28. Lee CK, Yoo SK. An analysis of the relationship between self-reported anxiety, depressiveness and parameters of heart rate variability based on photoplethysmography [Article in Korean]. Sci Emot Sens. 2012;3:345-354. URL: https:// koreascience.kr/article/JAKO201229665547045.pdf [Accessed 2026-03-21]
29. Stein PK, Carney RM, Freedland KE, et al. Severe depression is associated with markedly reduced heart rate variability in patients with stable coronary heart disease. J Psychosom Res. 2000;48(4-5):493-500. [doi: 10.1016/s0022 3999(99)00085-9] [Medline: 10880671]
30. Sayar K, Güleç H, Gökçe M, Ismail AK. Heart rate variability in depressed patients. Psychiatry Clin Psychopharmacol. 2002;12(3):130-133. URL: https://psychiatry-psychopharmacology.com/index.php/pub/article/view/313 [Accessed 2026-03-11]
31. Subramaniam V, R N. Relationship between heart rate variability and major depressive disorder in young adults. Swiss Arch Neurol Psychiatr Psychother. 2023;174:126-129. [doi: 10.4414/sanp.2023.03284]
32. Chung AH, Gevirtz RN, Gharbo RS, Thiam MA, Ginsberg JPJ. Pilot study on reducing symptoms of anxiety with a heart rate variability biofeedback wearable and remote stress management coach. Appl Psychophysiol Biofeedback. Dec 2021;46(4):347-358. [doi: 10.1007/s10484-021-09519-x] [Medline: 34308526]
33. Zhang ZX, Tian XW, Lim JS. Neuro-fuzzy network-based depression diagnosis algorithm using optimal features of HRV. The Journal of the Korea Contents Association. Feb 28, 2012;12(2):1-9. [doi: 10.5392/JKCA.2012.12.02.001]
34. Moshe I, Terhorst Y, Opoku Asare K, et al. Predicting symptoms of depression and anxiety using smartphone and wearable data. Front Psychiatry. 2021;12:625247. [doi: 10.3389/fpsyt.2021.625247] [Medline: 33584388]
35. Karimi S, Nateghi M, Cestero GI, et al. Prescreening depression using wearable electrocardiogram and photoplethysmogram data from a psycholinguistic experiment. Physiol Meas. Aug 2, 2025;46(8):085004. [doi: 10.1088/ 1361-6579/adf6fe] [Medline: 40752509]
36. Roh T, Hong S, Yoo HJ. Wearable depression monitoring system with heart-rate variability. Annu Int Conf IEEE Eng Med Biol Soc. 2014;2014:562-565. [doi: 10.1109/EMBC.2014.6943653] [Medline: 25570021]
37. Kojima M, Hayano J, Fukuta H, et al. Loss of fractal heart rate dynamics in depressive hemodialysis patients. Psychosom Med. Feb 2008;70(2):177-185. [doi: 10.1097/PSY.0b013e31816477a1] [Medline: 18256338]
38. Siddi S, Bailon R, Giné-Vázquez I, et al. The usability of daytime and night-time heart rate dynamics as digital biomarkers of depression severity. Psychol Med. Jun 2023;53(8):3249-3260. [doi: 10.1017/S0033291723001034] [Medline: 37184076]
39. Shah RV, Grennan G, Zafar-Khan M, et al. Personalized machine learning of depressed mood using wearables. Transl Psychiatry. Jun 9, 2021;11(1):338. [doi: 10.1038/s41398-021-01445-0] [Medline: 34103481]
40. Theofilis P, Oikonomou E, Lazaros G, et al. The association of depression with QT duration: a comparison between individuals younger or older than 65 years. Psychosom Med. Jan 1, 2023;85(1):18-25. [doi: 10.1097/PSY. 0000000000001152] [Medline: 36516316]
41. Huang M, Shah A, Su S, et al. Association of depressive symptoms and heart rate variability in Vietnam war-era twins: a longitudinal twin difference study. JAMA Psychiatry. Jul 1, 2018;75(7):705-712. [doi: 10.1001/jamapsychiatry.2018. 0747] [Medline: 29799951]
42. Sun G, Shinba T, Kirimoto T, Matsui T. An objective screening method for major depressive disorder using logistic regression analysis of heart rate variability data obtained in a mental task paradigm. Front Psychiatry. 2016;7:180. [doi: 10.3389/fpsyt.2016.00180] [Medline: 27867364]
43. Pedrelli P, Fedor S, Ghandeharioun A, et al. Monitoring changes in depression severity using wearable and mobile sensors. Front Psychiatry. 2020;11:584711. [doi: 10.3389/fpsyt.2020.584711] [Medline: 33391050]
44. Narziev N, Goh H, Toshnazarov K, Lee SA, Chung KM, Noh Y. STDD: short-term depression detection with passive sensing. Sensors (Basel). Mar 4, 2020;20(5):1396. [doi: 10.3390/s20051396] [Medline: 32143358]
45. Di Matteo D, Fotinos K, Lokuge S, et al. Automated screening for social anxiety, generalized anxiety, and depression from objective smartphone-collected data: cross-sectional study. J Med Internet Res. Aug 13, 2021;23(8):e28918. [doi: 10.2196/28918] [Medline: 34397386]
46. Otte Andersen T, Skovlund Dissing A, Rosenbek Severinsen E, et al. Predicting stress and depressive symptoms using high-resolution smartphone data and sleep behavior in Danish adults. Sleep. Jun 13, 2022;45(6):zsac067. [doi: 10.1093/ sleep/zsac067] [Medline: 35298650]
47. Saeb S, Zhang M, Karr CJ, et al. Mobile phone sensor correlates of depressive symptom severity in daily-life behavior: an exploratory study. J Med Internet Res. Jul 15, 2015;17(7):e175. [doi: 10.2196/jmir.4273] [Medline: 26180009]
48. Opoku Asare K, Terhorst Y, Vega J, Peltonen E, Lagerspetz E, Ferreira D. Predicting depression from smartphone behavioral markers using machine learning methods, hyperparameter optimization, and feature importance analysis: exploratory study. JMIR Mhealth Uhealth. Jul 12, 2021;9(7):e26540. [doi: 10.2196/26540] [Medline: 34255713]
49. Aledavood T, Luong N, Baryshnikov I, et al. Multimodal digital phenotyping study in patients with major depressive episodes and healthy controls (mobile monitoring of mood): observational longitudinal study. JMIR Ment Health. Feb 21, 2025;12:e63622. [doi: 10.2196/63622] [Medline: 39984168]
50. Xu X, Chikersal P, Doryab A, et al. Leveraging routine behavior and contextually-filtered features for depression detection among college students. Proc ACM Interact Mob Wearable Ubiquitous Technol. Sep 9, 2019;3(3):1-33. [doi: 10.1145/3351274]
51. Sverdlov O, Curcic J, Hannesdottir K, et al. A study of novel exploratory tools, digital technologies, and central nervous system biomarkers to characterize unipolar depression. Front Psychiatry. 2021;12:640741. [doi: 10.3389/fpsyt.2021. 640741] [Medline: 34025472]
52. Chikersal P, Doryab A, Tumminia M, et al. Detecting depression and predicting its onset using longitudinal symptoms captured by passive sensing: a machine learning approach with robust feature selection. ACM Trans Comput Hum Interact. 2021;28(1):1-41. [doi: 10.1145/3422821]
53. Kumagai N, Tajika A, Hasegawa A, et al. Predicting recurrence of depression using lifelog data: an explanatory feasibility study with a panel VAR approach. BMC Psychiatry. Dec 11, 2019;19(1):391. [doi: 10.1186/s12888-019 2382-2] [Medline: 31829206]
54. Kim H, Lee S, Lee S, Hong S, Kang H, Kim N. Depression prediction by using ecological momentary assessment, Actiwatch data, and machine learning: observational study on older adults living alone. JMIR Mhealth Uhealth. Oct 16, 2019;7(10):e14149. [doi: 10.2196/14149] [Medline: 31621642]
55. Wang R, Chen F, Chen Z, et al. StudentLife: assessing mental health, academic performance and behavioral trends of college students using smartphones. Presented at: UbiComp ’14: Proceedings of the 2014 ACM International Joint Conference on Pervasive and Ubiquitous Computing; Sep 13-17, 2014; Seattle, Washington. [doi: 10.1145/2632048. 2632054]
56. Fadul R, Alfalahi H, Shehhi AA, Hadjileontiadis L. Depressive disorder remote detection through touchscreen typing behaviour. Annu Int Conf IEEE Eng Med Biol Soc. Jul 2023;2023:1-4. [doi: 10.1109/EMBC40787.2023.10340393] [Medline: 38082634]
57. Wadle LM, Ebner-Priemer UW, Foo JC, et al. Speech features as predictors of momentary depression severity in patients with depressive disorder undergoing sleep deprivation therapy: ambulatory assessment pilot study. JMIR Ment Health. Jan 18, 2024;11:e49222. [doi: 10.2196/49222] [Medline: 38236637]
58. Yamamoto M, Takamiya A, Sawada K, et al. Using speech recognition technology to investigate the association between timing-related speech features and depression severity. PLoS ONE. 2020;15(9):e0238726. [doi: 10.1371/ journal.pone.0238726] [Medline: 32915846]
59. Demiroglu C, Beşirli A, Ozkanca Y, Çelik S. Depression-level assessment from multi-lingual conversational speech data using acoustic and text features. J Audio Speech Music Proc. Dec 2020;2020(1):17. [doi: 10.1186/s13636-020-00182-4]
60. Wang Y, Liang L, Zhang Z, et al. Fast and accurate assessment of depression based on voice acoustic features: a cross sectional and longitudinal study. Front Psychiatry. 2023;14:1195276. [doi: 10.3389/fpsyt.2023.1195276]
61. Jiang Z, Seyedi S, Griner E, et al. Multimodal mental health digital biomarker analysis from remote interviews using facial, vocal, linguistic, and cardiovascular patterns. IEEE J Biomed Health Inform. Mar 2024;28(3):1680-1691. [doi: 10.1109/JBHI.2024.3352075] [Medline: 38198249]
62. Ben-Zeev D, Scherer EA, Wang R, Xie H, Campbell AT. Next-generation psychiatric assessment: using smartphone sensors to monitor behavior and mental health. Psychiatr Rehabil J. Sep 2015;38(3):218-226. [doi: 10.1037/prj0000130] [Medline: 25844912]
63. Zhou Y, Yao X, Han W, Wang Y, Li Z, Li Y. Distinguishing apathy and depression in older adults with mild cognitive impairment using text, audio, and video based on multiclass classification and shapely additive explanations. Int J Geriat Psychiatry. Nov 2022;37(11):2345-2356. [doi: 10.1002/gps.5827]
64. Zhao Q, Fan HZ, Li YL, et al. Vocal acoustic features as potential biomarkers for identifying/diagnosing depression: a cross-sectional study. Front Psychiatry. 2022;13:815678. [doi: 10.3389/fpsyt.2022.815678] [Medline: 35573349]
65. Ye J, Yu Y, Wang Q, et al. Multi-modal depression detection based on emotional audio and evaluation text. J Affect Disord. Dec 1, 2021;295:904-913. [doi: 10.1016/j.jad.2021.08.090] [Medline: 34706461]
66. Silva WJ, Lopes L, Galdino MKC, Almeida AA. Voice acoustic parameters as predictors of depression. J Voice. Jan 2024;38(1):77-85. [doi: 10.1016/j.jvoice.2021.06.018] [Medline: 34353686]
67. Auerbach RP, Srinivasan A, Kirshenbaum JS, Mann JJ, Shankman SA. Geolocation features differentiate healthy from remitted depressed adults. J Psychopathol Clin Sci. May 2022;131(4):341-349. [doi: 10.1037/abn0000742] [Medline: 35230855]
68. Dai R, Kannampallil T, Zhang J, Lv N, Ma J, Lu C. Multi-task learning for randomized controlled trials: a case study on predicting depression with wearable data. Proc ACM Interact Mob Wearable Ubiquitous Technol. 2022;6(2):1-23. [doi: 10.1145/3534591]
69. Farhan AA, Yue C, Morillo R, et al. Behavior vs. introspection: refining prediction of clinical depression via smartphone sensing data. Presented at: 2016 IEEE Wireless Health (WH); Oct 25-27, 2026; Bethesda, MD. [doi: 10.1109/WH.2016. 7764553]
70. Jacobson NC, Chung YJ. Passive sensing of prediction of moment-to-moment depressed mood among undergraduates with clinical levels of depression sample using smartphones. Sensors (Basel). Jun 24, 2020;20(12):3572. [doi: 10.3390/ s20123572] [Medline: 32599801]
71. Choi JG, Ko I, Han S. Depression level classification using machine learning classifiers based on actigraphy data. IEEE Access. 2021;9:116622-116646. [doi: 10.1109/ACCESS.2021.3105393]
72. Luik AI, Zuurbier LA, Hofman A, Van Someren EJW, Tiemeier H. Stability and fragmentation of the activity rhythm across the sleep-wake cycle: the importance of age, lifestyle, and mental health. Chronobiol Int. Dec 2013;30(10):1223-1230. [doi: 10.3109/07420528.2013.813528] [Medline: 23971909]
73. George SV, Kunkels YK, Booij S, Wichers M. Uncovering complexity details in actigraphy patterns to differentiate the depressed from the non-depressed. Sci Rep. Jun 29, 2021;11(1):13447. [doi: 10.1038/s41598-021-92890-w] [Medline: 34188115]
74. Vanderlind WM, Beevers CG, Sherman SM, et al. Sleep and sadness: exploring the relation among sleep, cognitive control, and depressive symptoms in young adults. Sleep Med. Jan 2014;15(1):144-149. [doi: 10.1016/j.sleep.2013.10. 006] [Medline: 24332565]
75. Ali FZ, Parsey RV, Lin S, Schwartz J, DeLorenzo C. Circadian rhythm biomarker from wearable device data is related to concurrent antidepressant treatment response. NPJ Digit Med. Apr 29, 2023;6(1):81. [doi: 10.1038/s41746-023 00827-6] [Medline: 37120493]
76. Maglione JE, Ancoli-Israel S, Peters KW, et al. Depressive symptoms and circadian activity rhythm disturbances in community-dwelling older women. Am J Geriatr Psychiatry. Apr 2014;22(4):349-361. [doi: 10.1016/j.jagp.2012.09.003] [Medline: 23567424]
77. Smagula SF, Ancoli-Israel S, Blackwell T, et al. Circadian rest-activity rhythms predict future increases in depressive symptoms among community-dwelling older men. Am J Geriatr Psychiatry. May 2015;23(5):495-505. [doi: 10.1016/j. jagp.2014.06.007] [Medline: 25066948]
78. Minaeva O, Riese H, Lamers F, Antypa N, Wichers M, Booij SH. Screening for depression in daily life: development and external validation of a prediction model based on actigraphy and experience sampling method. J Med Internet Res. Dec 1, 2020;22(12):e22634. [doi: 10.2196/22634] [Medline: 33258783]
